# Transcriptional Response to Chronic Long‐Access Fentanyl Self‐Administration in Rat Habenula and Amygdala

**DOI:** 10.1111/adb.70179

**Published:** 2026-07-14

**Authors:** Robin Magnard, Daianna Gonzalez‐Padilla, Ege A. Yalcinbas, Emma Chaloux‐Pinette, Nicholas J. Eagles, Michael S. Totty, Patricia H. Janak, Leonardo Collado‐Torres, Kristen R. Maynard

**Affiliations:** ^1^ Department of Psychological and Brain Sciences, Krieger School of Arts and Sciences Johns Hopkins University Baltimore Maryland USA; ^2^ Lieber Institute for Brain Development Johns Hopkins Medical Campus Baltimore Maryland USA; ^3^ Department of Psychiatry and Behavioral Sciences Johns Hopkins School of Medicine Baltimore Maryland USA; ^4^ The Solomon H. Snyder Department of Neuroscience Johns Hopkins School of Medicine Baltimore Maryland USA; ^5^ Medical Scientist Training Program Johns Hopkins University School of Medicine Baltimore Maryland USA; ^6^ Department of Biostatistics Johns Hopkins Bloomberg School of Public Health Baltimore Maryland USA; ^7^ Kavli Neuroscience Discovery Institute Johns Hopkins University Baltimore Maryland USA; ^8^ Center for Computational Biology Johns Hopkins University Baltimore Maryland USA

## Abstract

Fentanyl is a potent synthetic opioid associated with overdose. However, little is known about fentanyl‐induced molecular adaptations in the habenula and amygdala, two brain regions implicated in opioid use and withdrawal. We performed bulk RNA‐sequencing in the rat habenula and amygdala to identify transcriptomic changes associated with fentanyl intake. Male rats self‐administered intravenous saline or fentanyl over 22–24 days. Ninety minutes following the final session, brains were collected for transcriptomic profiling. In Hb, we identified 453 differentially expressed genes (DEGs) between saline and fentanyl rats, with upregulated genes associated with synaptic transmission and ionic conductance. In the amygdala, we identified 3041 fentanyl‐associated DEGs with upregulated genes implicated in metabolic and vesicular functions. Downregulated genes in both regions were enriched for extracellular matrix functions. Integration of DEGs with single‐cell RNA‐sequencing data from rodents and humans revealed that fentanyl DEGs were enriched in specific habenula and amygdala cell type markers. Furthermore, fentanyl downregulated DEGs in the amygdala were enriched in genes associated with the risk for substance use disorders. Together, we define how fentanyl intake alters transcriptional programs in the rat habenula and amygdala, and we link these changes to specific human cell types and risk genes for neuropsychiatric disorders and addiction.

## Graphical abstract

Chronic intravenous long‐access fentanyl self‐administration in rats produced transcriptional remodeling in the habenula and amygdala. Bulk RNA‐sequencing identified 453 habenula and 3,041 amygdala differentially expressed genes, revealing upregulation of synaptic process and ionic conductance genes in habenula, upregulation of metabolic and vesicular genes in amygdala, and shared downregulation of extracellular matrix genes. Integration with rodent and human single‐cell RNA‐seq datasets mapped fentanyl‐associated genes to specific neuronal, glial, and vascular cell types, while integration with GWAS revealed an overlap of amygdala downregulated genes with schizophrenia and substance use disorder risk genes.

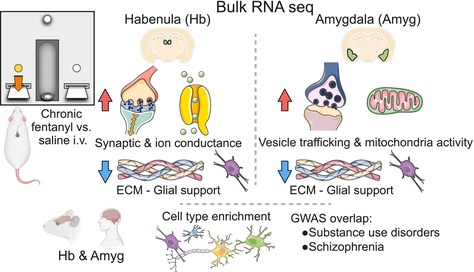

## 1 Introduction

Overdose deaths involving opioids have risen due to illicit use of synthetic opioids [1, 2, 3]. Fentanyl, a synthetic μ‐opioid receptor (MOR) agonist 50–100 times more potent than morphine or oxycodone [4], has disproportionately contributed to opioid‐related deaths [2, 5, 6]. Beyond potency, additional pharmacological properties distinguish fentanyl from other MOR agonists, including higher lipid solubility allowing rapid cell membrane penetration and receptor binding [4]. Thus, a deeper understanding of the molecular adaptations induced by fentanyl intake is needed to identify pathways that can be targeted to mitigate its effects.

Opioids cause long‐term molecular changes, especially in reward circuits involving the habenula (Hb) and amygdala (Amyg) [7, 8, 9]. The Hb is composed of two anatomically distinct structures, the medial habenula (MHb) and lateral habenula (LHb) [10]. While both regions are primarily glutamatergic, the MHb also co‐releases acetylcholine and substance P [11]. Functionally, the MHb mainly projects to the interpeduncular nucleus and contributes to withdrawal‐related negative affect [12], whereas the LHb acts as an anti‐reward centre encoding negative reward prediction errors through modulation of monoaminergic hubs, such as the ventral tegmental area (VTA) [13, 14]. The Hb has a high density of MOR [15, 16, 17], especially in the MHb [18], and optogenetic excitation of MOR‐expressing Hb neurons promotes aversion and avoidance [19]. Hb MOR‐expressing neurons also mediate aversive effects of naloxone‐precipitated withdrawal [20]. Despite the role of the Hb in opioid signalling it is unclear how opioids, including fentanyl, alter the transcriptional landscape of this key anti‐reward region.

The basolateral amygdala (BLA) and central amygdala (CeA) play a key role in emotional valence and affective behaviours [21, 22]. The BLA assigns value to environmental stimuli and associates the negative emotional state experienced during opioid withdrawal with contexts and cues [23, 24]. Accordingly, retrieval of morphine‐associated conditioned withdrawal memories requires BLA plasticity and coordinated activity within a BLA → prelimbic cortex loop [25]. The CeA mediates the expression of negative affective states of opioid withdrawal [21, 26]. Lesioning the CeA blocks morphine withdrawal‐induced conditioned place aversion [27]. A population of MOR‐expressing CeA neurons is activated during fentanyl withdrawal and drives aversive symptoms and negative reinforcement [28]. Furthermore, chronic fentanyl administration can induce hyperalgesia, and the CeA is implicated in this aversive state [29, 30]. Although the Amyg is important in mediating fentanyl‐induced withdrawal and negative affect, its molecular adaptations following chronic fentanyl self‐administration remain unexplored.

In rodents, acute or repeated injection of opioids induces gene expression dysregulation [31, 32, 33, 34, 35]. Similarly, chronic opioid self‐administration causes molecular changes in striatal and limbic circuits, including the nucleus accumbens (NAc), dorsal striatum (DS), VTA, prefrontal cortex and BLA, with many changes occurring dynamically across intake, abstinence, withdrawal and reinstatement phases [8, 36, 37, 38, 39, 40, 41, 42]. Altered gene expression in reward circuits after chronic long‐access fentanyl self‐administration has been reported [43, 44, 45], but these studies did not include Hb or Amyg. In postmortem human brain, limited studies have identified transcriptomic changes associated with opioid use disorder (OUD) in the DS, NAc and dorsolateral prefrontal cortex [46, 47]. In induced pluripotent stem cell (iPSC)‐derived models, single‐cell sequencing of forebrain organoids from patients with OUD showed that repeated exposure to different opioids leads to unique transcriptional responses [48]. Together, these studies highlight the importance of understanding fentanyl‐specific transcriptional programs across rodent and human Hb and Amyg.

Here, we performed bulk RNA‐sequencing (RNA‐seq) in the rat Hb and Amyg following chronic long‐access fentanyl self‐administration to identify shared and region‐specific molecular alterations. We identified differentially expressed genes (DEGs) between saline and fentanyl rats and integrated these data with single‐nucleus (sn) and single‐cell (sc) RNA‐seq data from rodent and human Hb and Amyg [49, 50, 51, 52] to identify cell types enriched in fentanyl DEGs. Finally, we investigated whether Hb and Amyg fentanyl DEGs are associated with risk for neuropsychiatric disorders, including OUD and substance use disorders (SUD) [53, 54, 55, 56, 57, 58].

## 2 Materials and Methods Summary

### 2.1 Experiment Summary and RNA‐Seq Data Generation

**FIGURE 1:**
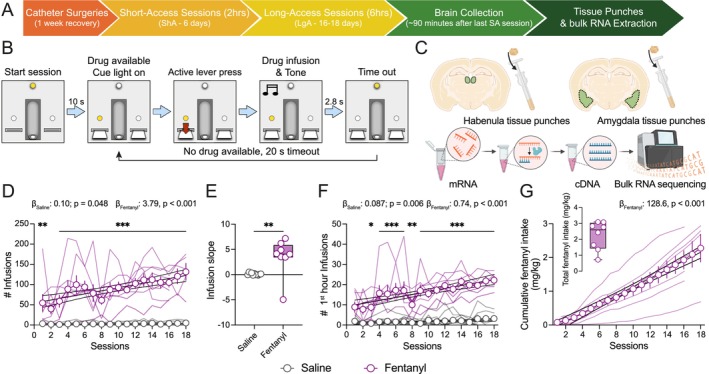
Escalation of fentanyl intake during chronic long‐access intravenous self‐administration. (A) Experimental timeline. Rats were implanted with jugular catheters for intravenous delivery of either saline or fentanyl. Rats first underwent six 2‐h acquisition sessions (Short‐Access, ShA), followed by 16–18 daily 6‐h sessions (Long‐Access, LgA). (B) Intravenous self‐administration task design. Fentanyl or saline infusion was contingent upon active lever press on a fixed‐ratio 1 (FR1) schedule of reinforcement. Drug or saline availability was signalled by cue light illumination and extinction of the house light. (C) Schematic of bilateral habenula and amygdala tissue collection for bulk RNA sequencing. (D) Mean number of infusions per LgA session for saline and fentanyl rats (mixed‐effects model [REML], substance effect: *F*_1,17_ = 80.47, *p* < 0.001; session effect: *F*_17,273_ = 5.98, *p* < 0.001; substance × session interaction: *F*_17,273_ = 5.41, *p* < 0.001; Šídák post hoc comparisons). (E) Individual rat infusion slopes across the LgA sessions (*t*‐test, substance effect *t*_17_ = 3.14, *p* = 0.006). (F) Mean number of infusions occurring within the first hour of each LgA session for saline and fentanyl rats (mixed‐effects model [REML], substance effect: *F*_1,17_ = 74.01, *p* < 0.001; session effect: *F*_17,273_ = 7.93, *p* < 0.001; substance × session interaction: *F*_17,273_ = 5.58, *p* < 0.001; Šídák post hoc comparisons). (G) Mean cumulative fentanyl intake (mg/kg) across LgA sessions. Insert: total overall amount of fentanyl intake per rat (mg/kg) during ShA and LgA sessions. Data shown as mean across rats ± SEM, superimposed with individual rat data points. Black lines represent linear regression with 95% confidence intervals. Boxes extend from the 25th–75th percentiles; lines within the boxes represent the median; whiskers indicate the minimum and maximum values. Saline: *n* = 11 rats; Fentanyl: *n* = 8 rats. * denotes *p* < 0.05; ** denotes *p* < 0.01; and *** denotes *p* < 0.001.

Adult male Sprague–Dawley rats (ENVIGO, Frederick, MD) were studied under a protocol approved by the Animal Care and Use Committee of Johns Hopkins University and conducted in accordance with the National Institutes of Health Guidelines for the Care and Use of Laboratory Animals. Jugular catheters were implanted as previously described [59], rats were trained for self‐administration of fentanyl citrate (Cayman Chemical) or saline in standard operant chambers housed inside sound‐attenuating chambers (Med Associates, St Albans, VT, USA) and behavioural data were tracked (Figures 1 and S1 and Tables S1–S3). After 22–24 training sessions, brains were collected, sectioned into ~2‐mm coronal slabs, and bilateral tissue punches were collected from the Hb and Amyg. Ribosomal RNA depletion and library preparation were performed and ~80 M paired‐end reads per sample were sequenced.

### 2.2 RNA‐Seq Data Processing and Quality Control

Read quality control, read alignment and gene expression quantification against the rat genome mRatBN7.2 was performed with *SPEAQeasy* [60]. Lowly expressed genes were filtered and normalization by sample library size and RNA composition was performed [61]. Sample QC metrics (Tables S3 and S4) were examined across brain regions and substances (Figure S2), RNA extraction batches (Figure S3) and number of total self‐administration sessions (Figure S4). Quality differed between brain regions; thus, Hb and Amyg samples were analysed separately in downstream analyses (Figure S2). Outlier identification of sample QC metrics (Figure S5) and Principal Component Analysis (Figure S6) revealed four Hb and four Amyg outlier samples (Figure S7), but manual inspection of all QC metrics considered for all these eight samples supported their inclusion for downstream differential gene expression (DGE) analysis (Figure S7, 16 708 genes across 33 samples).

### 2.3 Differential Gene Expression (DGE)

**FIGURE 2:**
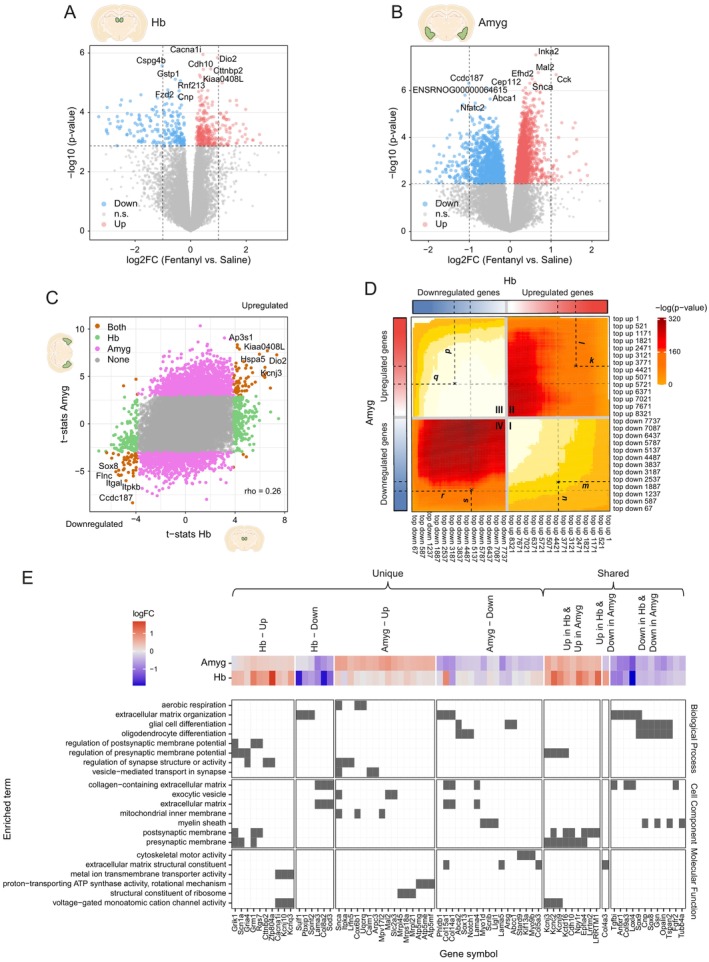
Transcriptional changes and gene ontology (GO) enrichment analysis in Hb and Amyg following chronic LgA fentanyl self‐administration. (A and B) Volcano plots showing up‐ and down‐regulated genes for fentanyl versus saline in Hb (A) and Amyg (B). Horizontal dashed line demarcates significance threshold at FDR = 0.05. Vertical dashed lines demarcate log_2_‐fold change values of −1 and 1. The top five most significantly up‐ and down‐regulated DEGs are labelled. (C) Substance (fentanyl vs. saline) *t*‐statistic correlation plot in Amyg versus Hb. Pink points are unique DEGs in Amyg. Green points are unique DEGs in Hb. Orange points are shared DEGs in Hb and Amyg. Positive *t*‐statistics indicate upregulation and negative *t*‐statistics downregulation after fentanyl self‐administration. (D) Heat map of one‐sided Fisher's exact test −log(*p*‐values) for the overlap between the top *k* up‐regulated genes in Hb and the top *l* up‐regulated genes in Amyg (quadrant II), the top *r* down‐regulated genes in Hb and top *s* down‐regulated genes in Amyg (quadrant IV), the top *m* up‐regulated genes in Hb and the top *n* down‐regulated genes in Amyg (quadrant I) and the top *q* down‐regulated genes in Hb and the top *p* up‐regulated genes in Amyg (quadrant III). (E) Biological processes, cellular components and molecular functions dysregulated by fentanyl in Hb and Amyg, as identified by Gene Ontology (GO) enrichment analysis of DEGs. Tile plot displays DEG (x‐axis) membership to a significantly enriched GO term as a filled tile. Key DEGs per term are shown, categorized by their unique or shared up‐ and down‐regulation in Hb and Amyg. Top heatmap shows DEG mean‐centred log_2_FC in Hb and Amyg. Related to Figures S9 and S10 and Tables S5, S6, S8 and S9.

DGE between fentanyl versus saline self‐administration and for behavioural traits among fentanyl‐administered rats was assessed separately in Hb and Amyg using *limma*‐*voom*. In each DGE model, differentially expressed genes (DEGs, FDR < 0.05) were identified after adjusting for batch effects and independent metrics contributing to gene expression variance (Figures 2 and S8).

### 2.4 Rank–Rank Hypergeometric Overlap Test

The overlap between the top *p* most significant (ranked by *p*‐value) up‐ or down‐regulated genes in one brain region and the top *q* most significant up‐ or down‐regulated genes in the other brain region was assessed across ranges of *p* and *q* top genes through one‐sided Fisher's exact tests, following its implementation in the *RRHO2* package [62] (Figure 2).

### 2.5 Functional Enrichment Analysis

Gene Ontology (GO) ontology terms and KEGG pathways affected by fentanyl self‐administration in Hb and Amyg were found by over‐representation analysis with *clusterProfiler* (Figures 2 and S10).

### 2.6 Cell‐Type Enrichment Analysis

**FIGURE 3:**
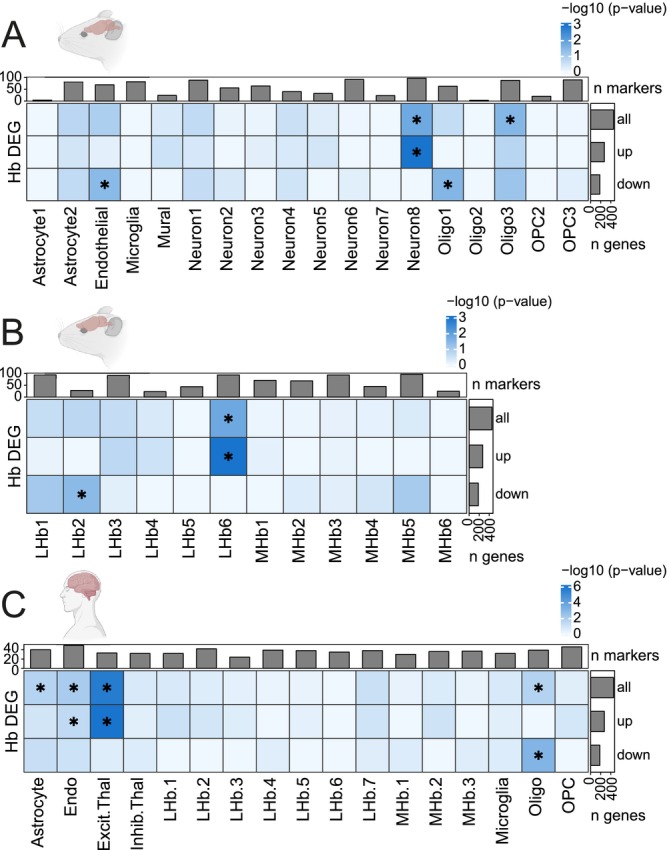
Enrichment of rat Hb fentanyl DEGs in specific human and rodent habenula cell types. (A and B) Enrichment of fentanyl versus saline DEGs (FDR < 0.05) found in rat Hb (y‐axis), among the rat orthologues of (A) the top 100 markers for broad‐level cell type clusters and (B) top 100 markers for neuronal subtypes previously identified in mouse habenula by Hashikawa et al. [50]. (C) Enrichment of rat fentanyl versus saline DEGs among the rat orthologues of the top 50 markers for broad‐level non‐neuronal and fine‐level neuronal subtypes we previously identified in human Hb/epithalamus in Yalcinbas et al. [49]. Note that mouse (Hashikawa et al. [50]) and human (Yalcinbas et al. [49]) Hb cell type names were not defined to match across datasets. Bar plots along the y‐axis depict the number of Hb DEGs that were upregulated, downregulated or all. Bar plots on top depict the number of rat orthologues used to define each cell cluster. The shade of blue indicates DEG enrichment *p*‐value for a cell type in the −log_10_ scale. * denotes *p* < 0.05. Related to Figures 2 and S11 and Tables S11 and S12.

**FIGURE 4:**
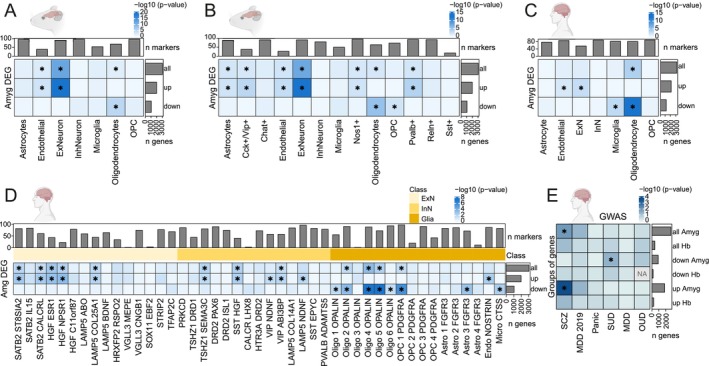
Enrichment of rat Amyg fentanyl DEGs in specific human and rodent amygdala cell types and association of Hb and Amyg rat DEGs with psychiatric disorders. Enrichment of fentanyl versus saline DEGs (FDR < 0.05) found in rat Amyg (y‐axis), among the top 100 markers for (A) broad‐level and (B) fine‐level cell type clusters identified in rat Amyg by Zhou et al. [51], as well as among the rat orthologues of the top 100 markers for (C) broad‐level and (D) fine‐level cell types identified in the human Amyg by Yu et al. [52]. Bar plots along the y‐axis depict the number of Amyg DEGs that were upregulated, downregulated, or all. Bar plots on top depict the number of marker genes used to define each cell cluster. The shade of blue indicates DEG enrichment *p*‐value for a cell type in the −log_10_ scale. * denotes *p* < 0.05. The light yellow, yellow and orange colours indicate the cell type class: excitatory neuron (ExN), inhibitory neuron (InN) or glia. (E) *MAGMA* gene‐set enrichment analysis of gene‐level Genome‐Wide Association Study (GWAS) signals in human orthologs of rat Hb and Amyg fentanyl versus saline DEGs. The y‐axis categorizes fentanyl versus saline DEGs into upregulated, downregulated or all, by brain region (Hb or Amyg). The x‐axis represents the five GWAS datasets that were probed. The shade of blue indicates enrichment *p*‐value in the −log_10_ scale. * denotes FDR‐adjusted *p* < 0.05. Given only one human gene whose rat ortholog was downregulated in Hb had OUD‐associated SNPs, *MAGMA* could not perform enrichment for this gene set (returned NA). SCZ, schizophrenia; MDD, major depressive disorder; SUD, substance use disorder; OUD, opioid use disorder. Related to Figure 2 and Tables S13 and S14.

The cell type specificity of DGE results in rat Hb and Amyg for fentanyl versus saline self‐administration was measured defining marker genes for fine and broad cell types in control rat Amyg [51], control human epithalamus [49] and Amyg [52] and control mouse Hb [50], with the *MeanRatio* method [63]. The sets of cell type marker genes in rat were assessed for their over‐representation among the rat DEGs in Hb and Amyg applying one‐sided Fisher's exact test (Figures 3 and 4).

### 2.7 Generalized Gene‐Set Analysis of GWAS Data

Associations of fentanyl self‐administration effects in Hb and Amyg with multiple psychiatric disorders were interrogated using *MAGMA* [64]. This method was provided with summary statistics from 5 GWAS datasets [53, 54, 55, 56, 57] to compute gene‐level associations with the GWAS disorders using the 1000 Genomes European Phase 3 panel [65] as reference. Human orthologs of rat Hb and Amyg fentanyl versus saline DEGs were obtained using *biomaRt* [66], and these were subsequently subjected to a competitive positive one‐sided gene‐set analysis to find associations at the gene‐set level according to the gene‐level associations (Figure 4).

For full methodological details, see supplemental methods.

## 3 Results

### 3.1 Chronic Long‐Access Fentanyl Self‐Administration Results in Escalation of Intake

A week after surgery, adult rats were trained to self‐administer saline or fentanyl in 6 short access sessions (ShA, 2 h) followed by 16–18 long‐access sessions (LgA, 6 h) (Figure 1A). Active lever presses resulted in infusion of 0.5 μg of fentanyl dissolved in saline, or saline alone (Figure 1B). Sixty to ninety minutes following the final LgA session, Hb and Amyg were collected for transcriptomic analysis (Figure 1C).

During the LgA phase, fentanyl rats showed higher engagement with the active versus inactive lever (Figure S1A,B), resulting in more fentanyl infusions and steeper infusion slopes compared to saline control (Figure 1D,E). This was consistent with the escalation ratio, in which we normalized the number of infusions obtained per rat across LgA sessions to the number of infusions from the first session (Figure S1C). Fentanyl escalation also remained evident when considering only the first hour of each LgA session (Figure 1F). On average, rats cumulated a total of 2.15 mg/kg of fentanyl across LgA sessions (Figure 1G). When combining both ShA and LgA phases, total fentanyl intake ranged from 0.719 to 3.089 mg/kg (Figure 1G insert), demonstrating a progressive escalation of fentanyl intake with repeated exposure (Figure S1D).

We next examined ‘futile’ lever presses, defined as non‐reinforced active lever presses made during the tone and timeout periods (Figure 1B). Compared to saline, fentanyl rats consistently made more lever presses during both periods (Figure S1E,F). As these presses are not reinforced, they may reflect increased motivation to obtain the drug with chronic exposure.

### 3.2 Shared and Unique Gene Expression Changes in Hb and Amyg Following Chronic Fentanyl Intake

To identify transcriptomic changes driven by escalating fentanyl self‐administration in Hb and Amyg, we performed bulk RNA‐seq from individual rat brains collected ~90 min after the final LgA session (Figure 1A). Following quality control analysis (Figures S2–S7), 16 Hb (8 saline; 8 fentanyl) and 17 Amyg (9 saline; 8 fentanyl) samples were retained. Hb and Amyg samples were analysed separately to assess correlations between biological, behavioural and technical variables, followed by gene expression variance partitioning to build parsimonious linear models for differential gene expression (DGE) analyses (Figure S8).

Region‐specific analyses between saline and fentanyl treated rats revealed 453 DEGs in the Hb (271 upregulated, 182 downregulated) and 3041 DEGs in the Amyg (1988 upregulated, 1053 downregulated) at FDR < 0.05 (Figures 2 and S9 and Tables S5 and S6). In the Hb, top upregulated genes, such as *Cdh10*, *Cttnbp2* and *Cacna1i*, are mainly involved in synaptic processes and calcium‐dependent signalling [67, 68, 69] (Figures 2A and S9). In contrast, in the Amyg, top upregulated genes, including *Mal2*, *Cck* and *Snca*, are linked to vesicle trafficking, mitochondrial organization and ATP synthesis [70, 71, 72] (Figures 2B and S9). Among the most significant Hb downregulated genes, *Cspg4b* contributes to oligodendrocyte precursor cell structure and myelination [73], *Gstp1* is central to oxidative stress defence and detoxification [74] and *Rnf213* participates in vascular homeostasis [75] (Figure 2A). In Amyg, the most significant downregulated genes included *Cep112*, involved in maintenance of centrosome integrity [76], *Abca1*, which mediates cholesterol transport and lipid homeostasis [77], and *Nfatc2*, which regulates calcium‐dependent transcription and neuroimmune signalling [78] (Figure 2B).

Transcriptional signatures were highly concordant between Hb and Amyg, as revealed by Rank‐Rank Hypergeometric Overlap (RRHO) test. This significance threshold‐free approach showed significant overlaps between the top most significantly upregulated genes in Hb and the top most significantly upregulated genes in Amyg and similarly for the top downregulated genes in both brain regions (Figure 2D). At the defined FDR < 0.05 threshold, a total of 106 DEGs were shared between Hb and Amyg, with all but six DEGs (*Dact3*, *Plcb4*, *Adarb2*, *Col4a3*, *Abhd17c* and *Ly6e*) regulated in the same direction (Table S7). *Col4a3* encoding the ⍺3 chain of type IV collagen, involved in ECM organization and vascular integrity [79], was oppositely regulated in Hb and Amyg (Table S7). Shared DEGs (Figure 2C and Table S7) also include the stress‐related receptors *Crhr2* (downregulated) and *Npy1r* (upregulated). Upregulation of the G protein–activated inwardly rectifying potassium (GIRK) channel subunits *Kcnj3* and *Kcnj9* was observed within the Hb–Amyg network [80].

Functional enrichment analyses using Gene Ontology (GO) terms and Kyoto Encyclopedia of Genes and Genomes (KEGG) showed region‐specific and shared biological signatures (Figures 2E and S10 and Tables S8 and S9). In the Hb, upregulated genes were primarily associated with synaptic transmission and ionic conductance, including regulators of postsynaptic membrane potential (*Grik1*, *Scn1a* and *Gria4*) and voltage‐gated cation channels (*Cacna1*, *Kcnj10* and *Kcnq3*, Figure 2E). In contrast, Amyg‐specific upregulated genes were enriched for mitochondrial metabolism (*Snca*, *Cox6b1*, *Uqcrq)* and vesicular functions (*Calm1* and *Arpc3*, Figure 2E). Shared upregulated genes across both regions converged on synaptic organization and excitability, including *Kcnc2*, *Kctd16*, *Cdh10* and *Epha4* (Figure 2E). In both regions, fentanyl was also associated with downregulation of ECM genes (*Sox9*, *Gsn* and *Col9a3*, Figure 2E and Tables S8 and S9).

KEGG pathway analysis revealed additional region‐specific functional specializations (Figure S10). In the Hb, upregulated genes were enriched for the KEGG term morphine addiction (*Adora1*, *Gabra4*, *Adcy8*). In contrast, Amyg showed upregulation of oxidative phosphorylation (*Atp6v1e1*, *Atp5mc2*, *Atp6v0e2*), Parkinson disease (*Snca*, *Calm1*, *Slc39a10*) and actin cytoskeleton genes (*Arpc3*, *Nckap1*, *Cfl1*), together with downregulation of other cytoskeletal regulators (*Vcl*, *C6*, *Wasf2*), suggesting a dynamic structural reorganization, coupled to elevated metabolic demand following fentanyl exposure. Finally, opposite regulation of *Col4a3* within ECM–receptor interaction and *Plcb4* across glutamatergic synapse and gastric acid secretion highlighted region‐specific transcriptional divergence in extracellular and excitatory signalling between the Hb and Amyg (Tables S8 and S9).

In addition, we examined transcriptional changes associated with behaviour‐related metrics among fentanyl rats with the first hour infusions slope of LgA sessions, total overall fentanyl intake across LgA sessions and fentanyl intake during the last LgA session (Tables S1, S2 and S10 and Figure S8). No significant gene‐behaviour relationship was observed in either the Hb or Amyg. This is likely due to limited statistical power and higher variability when subsetting to the eight fentanyl rats.

### 3.3 Enrichment of Genes Associated With Fentanyl Intake in Human and Rodent Hb and Amyg Cell Types

To infer cell types most impacted by fentanyl self‐administration in Hb and Amyg, we performed enrichment analyses leveraging 4 publicly available sc/snRNA‐seq datasets across rodent and human Amyg and Hb [49, 50, 51, 52]. We identified the top 100 or 50 marker genes for broad and fine‐level cell types in each dataset (Tables [Link], [Link], [Link], [Link]), obtained rat orthologs and assessed over‐representation among fentanyl versus saline DEGs in rat Hb and Amyg.

At the broad cell type level, upregulated fentanyl DEGs in rat Hb were enriched in a single mouse Hb neuron population (Neuron 8) [50], while downregulated DEGs were enriched in endothelial cells (Figure 3A and Table S11). At the fine cell type level, upregulated rat Hb fentanyl DEGs were enriched in a specific mouse LHb subpopulation (LHb.6) marked by *Kcnmb4*, *Fam101b*, *Chrm2*, *Sv2c* and *Gpr151* (Figure 3B and Table S11) [50]. We also observed enrichment of downregulated fentanyl DEGs in mouse LHb.2, marked by *Arpp21*, *Cacna2d1* and *Slc6a1*. We next evaluated whether rat fentanyl DEGs were enriched in orthologous Hb subpopulations in the human brain using our previously published human Hb snRNA‐seq dataset [49] (Figure 3C and Table S12). Upregulated and downregulated rat fentanyl DEGs registered to human astrocytes, oligodendrocytes and endothelial cells. We did not see enrichment in any neuronal subpopulations, likely due to limitations in power given the small number of Hb neurons in this human dataset. Interestingly, human Hb LHb.2 and LHb.7 show high expression of *KCNMB4, GPR151* and *CHRM2* (Figure S11A–C), suggesting these cell types may be conserved with mouse LHb.6, which showed an enrichment of upregulated fentanyl DEGs (Figure 3B and Table S11). Human LHb.2 and LHb.7 highly express *OPRM1* (Figure S11D), suggesting these may be fentanyl‐sensitive LHb populations conserved across species.

Next, we performed similar enrichment analyses of rat Amyg fentanyl‐associated DEGs in previously identified Amyg cell types from rat [51] and human [52] snRNA‐seq datasets. At the broad cell type level, upregulated rat fentanyl DEGs were enriched in endothelial cells and excitatory neurons, while downregulated fentanyl DEGs were enriched in oligodendrocytes (Figure 4A and Table S13). At the fine cell type level, upregulated fentanyl DEGs were still most strongly enriched in excitatory neurons, while downregulated DEGs remained enriched in oligodendrocytes and oligodendrocyte precursor cells (OPCs). However, we also observed enrichment of upregulated fentanyl DEGs in astrocytes and several inhibitory neuron populations, including *Cck*+/*Vip*+, *Nos1*+ and *Pvalb*+ (Figure 4B and Table S13). Notably, *Cck* is one of the top 5 upregulated genes following chronic fentanyl intake in Amyg (Figure 2). Enrichment analyses in human Amyg cell types largely agreed with findings in rodents. At the fine cell type level, upregulated rat fentanyl DEGs were enriched in Endo_NOSTRIN populations and several excitatory subpopulations in the BLA expressing *HGF*, *SATB2* and *LAMP5* (Figure 4C and Table S14). Similar to the rodent analysis, upregulated rat fentanyl DEGs were also enriched in several human inhibitory cell types, such as *SST*+ and *VIP*+ subpopulations and *TSHZ1*+ intercalated neurons. *TSHZ1*+ neurons receive input from VTA dopamine neurons and highly express dopamine receptor 1 (*DRD1*) and *OPRM1* [81, 82]. Downregulated rat fentanyl DEGs were enriched in several human glial cell types, including specific populations of oligodendrocytes, OPCs, astrocytes and microglia (Figure 4D and Table S14).

Finally, we asked whether fentanyl DEGs in Hb and Amyg overlapped with genes associated with risk for neuropsychiatric disorders as identified by GWAS [53, 54, 55, 56, 57, 58]. For Amyg, upregulated fentanyl DEGs were enriched in schizophrenia (SCZ)‐associated risk genes, while downregulated fentanyl DEGs were enriched in SUD‐associated risk genes. No enrichment was observed for fentanyl DEGs in Hb (Figure 4E). Taken together, we identify the enrichment of fentanyl‐associated genes in rodent and human Hb and Amyg cell types and demonstrate overlap of these genes with risk genes for SUD in Amyg.

## 4 Discussion

Here, we performed bulk RNA‐seq following chronic fentanyl self‐administration and identified unique and shared DEGs in the Hb and Amyg. We integrated fentanyl‐associated DEGs with sc/snRNA‐seq data and identified cell types enriched in fentanyl DEGs. Finally, we assessed the overlap of fentanyl DEGs with genes associated with risk for neuropsychiatric disorders and found enrichment of SUD risk genes among downregulated fentanyl DEGs in Amyg.

We observed both distinct and convergent transcriptional adaptations in the Hb and Amyg after fentanyl. In the Hb, synaptic transmission and ionic conductance genes were upregulated, suggesting heightened excitability. Downregulated Hb DEGs involved myelination, vascular regulation and oxidative stress defence, indicating weakened structural and metabolic support. In contrast, the Amyg showed enrichment for mitochondrial activity, ATP synthesis and vesicular pathways, consistent with increased energy demand and neurotransmitter release, alongside reduced expression of genes governing cytoskeletal organization, lipid metabolism and neuroimmune balance. In general, both regions exhibited upregulation of genes promoting synaptic organization and excitability and suppression of ECM and glial‐related genes. These findings align with postmortem human studies of individuals with OUD, which reveal disrupted synaptic plasticity, ECM and neuroimmune‐related functions in the PFC and NAc [46]. Additionally, neuroinflammatory and metabolic alterations across neuronal and glial populations were also observed in the DS of individuals who died from opioid overdose [47].

Our Amyg dataset also showed overlap with a dataset from mouse VTA after chronic fentanyl self‐administration [44], which reported downregulation of metabolic, mitochondrial and myelin‐related pathways and upregulation of neuronal excitability and synaptic plasticity genes. Focusing on Hb‐VTA overlap, 58 genes were significantly altered in both regions, of which a subset was concordantly regulated, while 24 DEGs were upregulated in Hb but downregulated in VTA. These included genes involved in synaptic organization, neuronal signalling and cytoskeletal regulation, metabolic and mitochondrial regulation and protein modification or stress responses. Given species differences and the distinct brain collection time points (24 h vs. 90 min), these comparisons should be interpreted cautiously. Yet, this opposite transcriptional profile supports region‐specific fentanyl effects, with enhanced structural plasticity and metabolic demand in the Hb, contrasting with a dampening in the VTA. These results align with LHb photometry recordings during oral fentanyl self‐administration [83], where increases in drug‐evoked LHb activity across 3 weeks of training suggest plasticity accompanying the transition from positive to negative reinforcement.

Fox et al. reported transcriptional changes in the NAc following oral fentanyl abstinence [84]. GO term enrichment analysis revealed a partial overlap with those identified in our Hb and Amyg dataset, including mitochondrial, ECM, cytoskeletal and synaptic processes. The convergence between these datasets is observed despite differences in species, brain regions and experimental paradigms.

To determine which cell types are impacted by chronic fentanyl self‐administration, we integrated our fentanyl DEGs with sn/scRNAseq data from rodent and human Hb and Amyg. Consistent with previous sc/snRNA‐seq studies that identified opioid‐related transcriptional changes in glial populations in the rodent NAc and Amyg [31, 35, 41], we found enrichment of our fentanyl DEGs in oligodendrocytes, astrocytes and endothelial cells in both Hb and Amyg. These findings were conserved across species and highlight that fentanyl impacts cell types critical for blood brain barrier (BBB) integrity and myelination. This is consistent with work showing that opioids alter BBB homeostasis, structure and integrity [85, 86, 87]. Furthermore, myelin pathology has been implicated in OUD [88, 89] and chronic methadone treatment of primary rat glia cultures increases oligodendrocyte apoptosis and reduces myelinating capacity [90]. Future functional studies should investigate how fentanyl intake dysregulates the neurovascular unit.

Beyond glial cells, we also observed enrichment of upregulated and downregulated rat Hb fentanyl DEGs in specific mouse LHb cell types identified by Hashikawa et al. [50]. In particular, upregulated DEGs were enriched in mouse LHb.6, which shares marker genes with two *OPRM1*‐expressing human Hb populations, LHb.2 and LHb.7 [49], suggesting possible cross‐species conservation of fentanyl‐sensitive *OPRM1*‐expressing LHb subpopulations. Notably, foot shock induces activity‐dependent gene expression in LHb.6 [50], implicating this population in the processing of painful noxious stimuli and providing further support for the functional relevance of this LHb cell type in opioid signalling. Unfortunately, direct enrichment analyses of rat Hb fentanyl DEGs in human Hb cell types did not yield any significant results, likely due to a lack of power resulting from the limited number of human Hb cells in our dataset.

In the Amyg, upregulated rat fentanyl DEGs were enriched in several excitatory and inhibitory populations, whereas downregulated DEGs were exclusively enriched in glial cell types, especially oligodendrocytes. We observed enrichment of upregulated rat fentanyl DEGs in multiple human fine level excitatory Amyg cell types, likely representing BLA excitatory populations. This is consistent with previous work showing that BLA glutamatergic neurons have increased intrinsic excitability and excitatory/inhibitory ratio in mice exposed to oral fentanyl consumption and withdrawal [91]. *VIP*+ interneurons and *TSHZ1* + intercalated neurons also showed enrichment in upregulated genes in both rodents and humans. There is mounting evidence for interactions between *VIP*+ interneurons and the opioid system, with *VIP*+ neurons showing *Oprm1* expression across many brain regions [92, 93]. *TSHZ1* + intercalated neurons are highly conserved across species [82], densely express *Oprm1* and act to inhibit BLA and CeA circuitry under basal conditions [94, 95]. However, *Oprm1* activation leads to inhibition of intercalated neurons, thus disinhibiting BLA and CeA reward circuitry underlying relapse of drug seeking behaviour [96]. Together, these findings point towards a conserved opioid‐driven disruption of glial support and inhibitory microcircuits that shifts Amyg function towards the disinhibition of neural circuits underlying relapse.

We acknowledge several limitations that motivate future investigations. First, molecular profiling was performed at one timepoint to capture transcriptomic changes following chronic fentanyl use while avoiding acute withdrawal effects. Future studies should examine changes across acquisition, withdrawal and relapse phases, particularly in the Hb given its role in opioid withdrawal [97, 98, 99]. Second, bulk RNA‐seq was performed on the entire Hb and Amyg rather than on their respective subregions. Given the distinct connectivity and functions of the MHb and LHb as well as BLA and CeA [7, 10, 22], subregions‐specific transcriptional adaptations to fentanyl volitional intake may have been masked by analysing these structures as a whole. Therefore, while bulk RNA‐seq is high throughput and cost‐effective, future investigations combining subregion sampling and higher resolution technologies such as snRNA‐seq will be needed to resolve subregions and cell type‐specific changes. Third, we acknowledge limitations in power that prevented the identification of DEGs associated with behavioural measures in fentanyl‐administering rats, including total fentanyl intake. Future experiments with larger numbers of rats should leverage more complex behavioural analyses using tools such as machine learning‐based behavioural tracking [100], for integration with transcriptomic data. Furthermore, given reported sex differences in humans and rodents in the context of opioids [43, 101, 102, 103], future studies should also include female rats as only males were assessed in the present study.

In summary, we report transcriptional programs associated with fentanyl intake in the rat Hb and Amyg and link fentanyl‐associated gene expression changes to specific cell types in the rodent and human brain. We provide evidence that genes altered by fentanyl in the rat Amyg overlap with genes implicated in genetic risk for neuropsychiatric disorders, including SUD. Taken together, these findings highlight cell types and molecular pathways for further functional follow‐up that could be targeted for potential therapeutic development.

## Author Contributions

Conceptualization: D.G‐P., E.A.Y., E.C‐P., P.H.J., L.C‐T., K.R.M. Software: D.G‐P., L.C‐T. Formal analysis: R.M., D.G‐P., E.A.Y., E.C‐P., N.J.E. Investigation: E.A.Y., E.C‐P., P.H.J., K.R.M. Resources: P.H.J., L.C‐T., K.R.M. Data curation: R.M., D.G‐P., E.A.Y., M.S.T. Writing – original draft: R.M., D.G‐P., E.A.Y., P.H.J., L.C‐T., K.R.M. Writing – review and editing: R.M., D.G‐P., E.A.Y., E.C‐P., P.H.J., L.C‐T., K.R.M. Visualization: R.M., D.G‐P., E.A.Y. Supervision: P.H.J., L.C‐T., K.R.M. Project administration: P.H.J., L.C‐T., K.R.M. Funding acquisition: P.H.J., L.C‐T., K.R.M. P.H.J., L.C‐T., and K.R.M. had full access to all the data in the study and take responsibility for the integrity of the data and the accuracy of the data analysis.

## Funding

This work was supported with funding from the Lieber Institute for Brain Development and National Institutes of Health (NIH US) grants T32MH015330 (Yalcinbas), R21DA060407 (Maynard) and R01DA035943 (Janak).

## Conflicts of Interest

The authors declare no conflicts of interest.

## Supporting information


**Data S1:** Supporting information.

**Figure S1:** Additional behaviour metrics from LgA sessions. Mean number of total (A) active (mixed‐effects model [REML], substance effect: *F*_1,17_ = 10.97, *p* = 0.0041; session effect: *F*_17,273_ = 1.44, *p* = 0.118; substance × session interaction: *F*_17,273_ = 1.4, *p* = 0.136; Šídák post hoc comparisons) and (B) inactive lever presses per LgA session for saline and fentanyl rats (mixed‐effects model [REML], substance effect: *F*_1,17_ = 0.010, *p* = 0.918; session effect: *F*_17,273_ = 1.26, *p* = 0.214; substance × session interaction: *F*_17,273_ = 1.19, *p* = 0.265). (C) Escalation ratio, an alternative metric to quantify infusion escalation across sessions. This ratio is calculated by normalizing each rat's LgA infusion counts relative to their infusion count on the first LgA session (mixed‐effects model [REML], substance effect: *F*_1,17_ = 8.77, *p* = 0.0088; session effect: *F*_17,273_ = 4.69, *p* < 0.001; substance × session interaction: *F*_17,273_ = 2.81, *p* < 0.001; Šídák post hoc comparisons). (D) Fentanyl intake (μg/kg) per LgA session. (E) Number of active lever presses performed during the 2.8 s infusion and tone presentation period (mixed‐effects model [REML], substance effect: *F*_1,17_ = 19.4, *p* < 0.001; session effect: *F*_17,273_ = 2.91, *p* < 0.001; substance × session interaction: *F*_17,273_ = 2.82, *p* < 0.001; Šídák post hoc comparisons). (F) Number of active lever presses performed during the 20‐s timeout period (mixed‐effects model [REML], substance effect: *F*_1,17_ = 3.06, *p* = 0.098; session effect: *F*_17,273_ = 1.41, *p* = 0.131; substance × session interaction: *F*_17,273_ = 1.41, *p* = 0.129). Data shown as mean across rats ± SEM. Black lines represent linear regression with 95% confidence intervals. Saline: *n* = 11 rats; fentanyl: *n* = 8 rats. * denotes *p* < 0.05; ** denotes *p* < 0.01; *** denotes *p* < 0.001.

**Figure S2:** Quality control metrics for Hb and Amyg samples. Comparison of the QC metrics examined in this study for habenula and amygdala fentanyl and saline samples. Note that different Illumina library preparation kits were used for each brain region, thus confounding brain region and kit differences, which motivated independent analyses for each brain region. See Table S3 for the description of these QC metrics.

**Figure S3:** Quality control metrics for samples across RNA extraction batches. Comparison of QC metrics of samples from the first (only Hb samples), second (only Amyg samples) and third batch for RNA extraction (additional Hb and Amyg samples). See Table S3 for the description of these QC metrics.

**Figure S4:** Quality control metrics for samples across total number of self‐administration sessions. Comparison of QC metrics for (Hb and Amyg) samples from rats who had 22 and 24 total (fentanyl or saline) self‐administration sessions. See Table S3 for the description of these QC metrics.

**Figure S5:** Low‐quality sample identification. Detection of low‐quality metrics for (A) Hb and (B) Amyg fentanyl (filled circles/squares) and saline (empty circles/squares) samples. QC metric outliers (in red) were identified as those being 3 median‐absolute‐deviations (MAD; dotted lines) away from the median (solid line). Only lower outliers were considered poor‐quality for all QC metrics except mitoRate, for which higher outliers were considered instead (indicated by arrows). Samples with outlier QC metrics are labelled and were subjected to further evaluation in downstream analyses (Figure S7). See Table S3 for the description of these QC metrics.

**Figure S6:** Principal component analysis. PC1 versus PC2 for gene expression in (A) Hb and (B) Amyg samples. Percentages of variance explained by each PC are indicated on the axes. Samples are shaped by RNA extraction batch and coloured by substance.

**Figure S7:** Manual sample quality examination based on PCA. PCx versus PCy (top) for (A) Hb and (B) Amyg samples. QC metrics outlier samples are labelled in red (see Figure S5); samples segregated from the rest in each PC plot, as well as fentanyl and saline samples closer to samples from the other substance group were considered PCA outlier samples and are labelled in purple. The percentage of variance explained by each PC is shown on axis labels. For both, QC metrics and PCA outlier samples, all their QC metrics were re‐examined (bottom box plots); different coloured squares indicate the different outlier samples. Samples in all plots are coloured by substance. See Table S3 for the description of these QC metrics.

**Figure S8:** Sample‐level covariate selection for DGE analysis. Gene expression variance partition analysis in (A) Hb (all rats), (B) Amyg (all rats), (C) Hb (fentanyl rats only) and (D) Amyg (fentanyl rats only). Top left: density plot for the percentages of variance explained in the expression of each gene by each sample‐level variable. Top right: canonical correlation between each pair of variables. Variables included in the models for DGE analyses (A–B for substance and C–D for rat behavioural traits) were selected based on their contributions to gene expression variance and correlations with other variables. Bottom: percentage of variance in the expression of each gene explained by each variable included in the DGE model, considering all other included variables in the model (x‐axis); variables are ordered by decreasing median percentage of variance explained. Related to Figure 2. See Table S3 for the description of these variables and QC metrics.

**Figure S9:** Top 5 differentially expressed genes in Hb and Amyg following chronic LgA fentanyl self‐administration. (A–B) Box plots showing the expression of the top five up‐ and down‐regulated DEGs for fentanyl versus saline in Hb (A) and Amyg (B). Gene expression is given in log_2_(CPM) after regressing out covariates. FDR‐adjusted *p*‐value and fold change (FC) are shown for each gene. Boxes extend from the 25th to 75th percentiles; lines within the boxes represent the median; whiskers indicate the minimum and maximum values, superimposed with individual rat data points. Fentanyl Hb *n* = 8; saline Hb *n* = 8; fentanyl Amyg *n* = 8; saline Amyg *n* = 9. Related to Figure 2.

**Figure S10:** Biological KEGG pathways dysregulated by chronic fentanyl self‐administration in Hb and Amyg. Tile plot displays DEG (x‐axis) membership to an enriched pathway as a filled tile. Key DEGs from each pathway are shown, categorized by their unique or shared up‐ and down‐regulation in Hb and Amyg. Top heatmap shows DEG mean‐centred log_2_FC in Hb and Amyg. Related to Figure 2 and Tables S8 and S9.

**Figure S11:** Expression of *KCNMB4*, *GPR151, CHRM2* and *OPRM1* in human habenula cell types. (A–D) Violin plots showing expression of (A) *KCNMB4*, (B) *GPR151*, (C) *CHRM2* and (D) *OPRM1* in human Hb cell types from Yalcinbas et al. [49]. *Kcnmb4*, *Gpr151* and *Chrm2* mark the mouse LHb.6 subpopulation identified by Hashikawa et al. [50], which we found to be enriched in our rat upregulated Hb fentanyl DEGs. These genes are highly expressed in human LHb.2 and LHb.7 subpopulations, which also express *OPRM1*. This suggests that fentanyl‐sensitive mouse LHb.6 may be conserved with these *OPRM1*‐expressing human LHb.2 and LHb.7 neuronal populations. Related to Figure 3.

**Table S1:** Behavioural raw data per LgA self‐administration session per rat. Session‐by‐session raw behavioural data pertaining to operant (lever press) behaviours and infusions from each rat across all long‐access self‐administration sessions. See Table S3 for the description of these variables.

**Table S2:** Behavioural rat data in LgA sessions. Individual rat data for behavioural covariates relating to fentanyl intake and intake escalation across long‐access self‐administration sessions. See Table S3 for the description of these variables.

**Table S3:** Dictionary of sample variables. Description of sample/rat variables analysed throughout the study. Related to Tables S1, S2 and S4.

**Table S4:** Sample metadata and QC metrics. Sample level variables analysed, including data regarding rat self‐administration sessions and sample batches for RNA extraction, library preparation and sequencing, as well as quality control metrics. See Table S3 for the description of these variables.

**Table S5:** DEGs for substance in Hb. Metadata, *limma* DE statistics and Ensembl gene annotation for DEGs obtained for fentanyl versus saline in Hb. See *limma* (114) documentation for these statistics definitions. Related to Figure 2 and Tables S8 and S10.

**Table S6:** DEGs for substance in Amyg. Metadata, *limma* DE statistics and Ensembl gene annotation for DEGs obtained for fentanyl versus saline in Amyg. See *limma* (114) documentation for these statistics definitions. Related to Figure 2 and Tables S9 and S10.

**Table S7:** Common DEGs for substance in habenula and amygdala. Metadata, region‐specific *limma* DE statistics and Ensembl gene annotation for overlapping DEGs for fentanyl versus saline in habenula and amygdala. See *limma* (114) documentation for these statistics definitions. Related to Figure 2 and Tables S5 and S6.

**Table S8:** Functional enrichment results for substance DEGs in Hb. GO terms for biological processes (BP), molecular functions (MF), cellular components (CC) and KEGG pathways that are significantly enriched in up‐ and down‐regulated DEGs for fentanyl versus saline in Hb. Provided are the ID and description of each significant term/pathway, the number and fraction of up/down‐regulated DEGs annotated to each term (Count and GeneRatio, respectively) and the list of such genes (geneID), the fraction of genes in universe annotated to each term (BgRatio), fold of enrichment, *p*‐value and FDR‐corrected *p*‐value. Related to Figures 2 and S10 and Table S5.

**Table S9:** Functional enrichment results for substance DEGs in Amyg. Same as Table S8 but for up‐ and down‐regulated DEGs for fentanyl versus saline in Amyg. Related to Figures 2 and S10 and Table S6.

**Table S10:** Results for all DGE analyses and genes in Hb and Amyg. Gene‐level metadata and *limma* DE statistics of each gene for substance and rat behaviour DGE analyses (fentanyl vs. saline, first hour infusion slope, total intake and last session intake) in Hb and Amyg. See *limma* (114) documentation for these statistics definitions. Related to Figure 2. This table includes all the data from Tables S5–S7.

**Table S11:** Top 100 *MeanRatio* marker genes per cell type in mouse Hb. For all cell subpopulations and Hb neuronal subpopulations in the Hb complex of control mice obtained in Hashikawa et al. [50], the top 100 most specific marker genes for each based on the *MeanRatio* method are reported. See *DeconvoBuddies* [63] documentation for column description. Cell_type_resolution column corresponds to the resolution of the cell subpopulation for which the gene is a marker. Related to Figure 3.

**Table S12:** Top 50 *MeanRatio* marker genes per cell type in human Hb. For broad and fine cell types in the human Hb‐enriched epithalamus of neurotypical control donors obtained in Yalcinbas et al. [49], the top 50 most specific marker genes for each based on the *MeanRatio* method are reported. See *DeconvoBuddies* [63] documentation for column description. Cell_type_resolution column corresponds to the resolution of the cell type for which the gene is a marker. Related to Figure 3.

**Table S13:** Top 100 *MeanRatio* marker genes per cell type in rat Amyg. For main cell types and inhibitory neuronal subtypes in the Amyg of control rats obtained in Zhou et al. [51], the top 100 most specific marker genes for each based on the *MeanRatio* method are reported. See *DeconvoBuddies* [63] documentation for column description. Cell_type_resolution column corresponds to the resolution of the cell type for which the gene is a marker. Related to Figure 4.

**Table S14:** Top 100 *MeanRatio* marker genes per cell type in human Amyg. For broad and fine cell types in the human Amyg of neurotypical control donors obtained in Yu et al. [52], the top 100 most specific marker genes for each based on the *MeanRatio* method are reported. See *DeconvoBuddies* [63] documentation for column description. Cell_type_resolution column corresponds to the resolution of the cell type for which the gene is a marker. Related to Figure 4.

## Data Availability

The source FASTQ files are publicly available from the NCBI Sequence Read Archive BioProject PRJNA1179901. All analysis code is available at https://github.com/LieberInstitute/fentanyl_rat_hb_amy [104].
