# Supplementary material for: Transcriptional Response to Chronic Long‐Access Fentanyl Self‐Administration in Rat Habenula and Amygdala
Source: Addict Biol. 2026 Jul 14;31(7):e70179. doi: 10.1111/adb.70179 (PMC13366401; doi:10.1111/adb.70179)
Supplement: Supplementary file 1 — Data S1: Supporting information. [file ADB-31-e70179-s012.docx]

# Supplementary Methods

## Rats

## Adult male Sprague-Dawley rats (ENVIGO, Frederick, MD) were used in all experiments. Three fentanyl rats were excluded during self-administration due to loss of catheter patency, resulting in a final sample of n = 11 saline and n = 8 fentanyl rats analyzed in this study. Rats were single-housed in a temperature and humidity-controlled vivarium on a normal light cycle (12/12 hr. light/dark cycle, lights ON at 7 am) with ad libitum access to food and water. The protocol was approved by the Animal Care and Use Committee of Johns Hopkins University and was conducted in accordance with the National Institutes of Health Guidelines for the Care and Use of Laboratory Animals.

##

## Drugs

Fentanyl Citrate (Cayman Chemical) was diluted in 0.9% sterile saline at 10 ug/mL. Brevital sodium (Henry Schein) was diluted to 10 mg/mL with 0.9% sterile saline.

## Surgeries

Adult male rats, weighing 250-300 grams at the time of surgery, were anesthetized with isoflurane (3-5% induction, 1-2% maintenance) and administered the analgesic rimadyl (5 mg/kg, SC, Zoetis) and the antibiotic cefazolin (70 mg/kg, SC, West-Ward) prior to catheter implantation. A silastic jugular catheter, constructed as described previously [(59)](https://sciwheel.com/work/citation?ids=3174299&pre=&suf=&sa=0), was threaded under the skin from a subcutaneous base in the mid-scapular region and inserted into the right jugular vein [(59)](https://sciwheel.com/work/citation?ids=3174299&pre=&suf=&sa=0). Catheters were flushed daily with ~0.1 mL of sterile saline solution containing 10 mg/mL gentamicin sulfate (VetOne) and 100 IU/mL Heparin (Sagent). Once behavioral training began, catheters were flushed before and after self-administration (and daily on rest days), and were assessed daily for blood return. In the event that blood return was absent, rats were administered 0.1 mL of brevital sodium (10 mg/mL in sterile saline, Henry Schein). If the rat did not become ataxic within 10 seconds, the catheter was considered not patent and the rat was removed from the study.

## Self-administration training

After recovery, rats were trained in standard Med Associates operant chambers housed inside sound-attenuating chambers (Med Associates, St Albans, VT, USA). Operant chambers were fitted with two retractable levers (active and inactive), stimulus lights above each lever, speakers, and a house light. Boxes were controlled using Med-PC IV software (Med Associates, St Albans, VT, USA).

Rats were trained to lever-press on a fixed-ratio 1 schedule, initially in short-access 2-hour sessions, and then progressing to long-access 6-hour sessions (6 days per week). Both levers were extended for the entire session duration. The stimulus light above the active lever indicated drug (or saline) availability. For fentanyl rats, active lever presses resulted in infusion of 0.5 µg fentanyl dissolved in 50 µl sterile saline delivered over 2.8 seconds [(105)](https://sciwheel.com/work/citation?ids=10476141&pre=&suf=&sa=0), while saline rats received an equivalent volume of sterile saline. Infusion was accompanied by a 2.8 second tone, followed by an additional 20 second timeout during which the stimulus light and house light were extinguished; active lever responses during this 22.8 sec interval were not reinforced. After 20 seconds elapsed, the house light and the stimulus light were re-illuminated to indicate availability of drug (or saline). Training occurred for 22-24 sessions, after which the brains were collected. Behavioral data for all the rats included in this study are provided ([**Figure 1**](#fig_behavior), [**Figure S1**](#sfi_behav_metrics), [**Table S1**](#sta_SA_sessions_behavioral_data_raw)**,** [**Table S2**](#sta_Rat_behavioral_covariates), [**Table S3**](#sta_sample_variables_dictionary)). The escalation ratio, used as an additional metric of infusion escalation across sessions, was calculated by normalizing each rat’s LgA infusion counts relative to its infusion count on the first LgA session.

## Behavioral analysis

Behavioral data were analyzed using mixed-effects models with restricted maximum likelihood (REML) estimation to account for repeated measures across sessions and unequal numbers of observations per subject. For all analyses, session was treated as a within-subject factor and substance (fentanyl vs. saline) as a between-subject factor. Post hoc comparisons were performed using Šídák correction for multiple comparisons.

***Brain tissue extraction and RNA processing***

Rats were lightly anesthetized with isoflurane and rapidly decapitated 60-90 minutes after the last long-access self-administration session, and extracted brains were fresh frozen in isopentane and stored at −80 °C. For tissue punching, brains were sectioned into ~2 mm coronal slabs using a rat brain matrix (Stainless Steel Alto Coronal, 1.0 mm matrix, Small Rat 175-300gm), and bilateral tissue punches were collected from the Hb (#39443001RM, Leica Biosystems, nominal diameter 1.25 mm) and Amyg (nominal diameter 1.25 mm) on petri dishes placed in a dry ice bucket. Punched tissue was ejected into tubes sitting in dry ice, and sample tubes were stored at −80 °C until ready for RNA processing. Total RNA was isolated by using TRIzol Reagent homogenization (#15596018, Invitrogen) and chloroform layer separation. RNA was then purified using an RNeasy Micro (#74004, Qiagen) kit with an RNase-Free DNase step (Mat. No. 1023460, Qiagen) according to manufacturer’s instructions. RNA concentration and purity were measured using a NanoDrop Eight (ThermoScientific). RNA quality control assays were performed on the Agilent 2100 Bioanalyzer, and the RNA integrity number for all samples ranged from 7.2 to 8.6 (mean ±SEM = 7.9 ± 0.06).

## Library construction and bulk RNA sequencing (RNA-seq)

Total RNA was extracted from habenula and amygdala tissue samples using the Qiagen RNeasy Micro Kit (Cat. No.: 74004). Paired-end strand-specific sequencing libraries were prepared for 33 samples (16 habenula, 17 amygdala) from 100 ng total RNA input for amygdala samples, and 10 ng total RNA input from low-yield habenula samples. For amygdala samples, TruSeq Stranded Total RNA Library Preparation kit with Ribo-Zero H/M/R Gold ribosomal RNA depletion was used (min. 0.5 ug of total RNA needed per sample). For low-yield habenula samples, Illumina Stranded Total RNA Prep Library Preparation kit with Ribo-Zero Plus was used (~1 ng to 1000 ng of total RNA needed per sample). For quality control, synthetic External RNA Controls Consortium (ERCC) RNA Mix 1 (Thermo Fisher Scientific) was spiked into each sample. Libraries were sequenced on an Illumina NovaSeq 6000 S4 (152 bp PE) producing ~80 million (median 102,396,054, mean 98,533,714) 152 bp paired-end reads per sample.

## RNA-seq data processing

### Gene expression quantification

*SPEAQeasy* pipeline version 87ba0b4 [(60)](https://sciwheel.com/work/citation?ids=10988515&pre=&suf=&sa=0), a *Nextflow* v20.01.0 [(106)](https://sciwheel.com/work/citation?ids=3552253&pre=&suf=&sa=0) workflow for *HISAT2* v2.2.1 [(107)](https://sciwheel.com/work/citation?ids=7266361&pre=&suf=&sa=0), was used with default settings to assess the quality of the sequencing reads and quantify the gene expression in the samples using the rat genome assembly mRatBN7.2 from Ensembl release 109 [(108,109)](https://sciwheel.com/work/citation?ids=11724063,13870406&pre=&pre=&suf=&suf=&sa=0,0). A *RangedSummarizedExperiment* R object [(110)](https://sciwheel.com/work/citation?ids=111791&pre=&suf=&sa=0) with gene counts for 30,452 genes across 33 samples was built by *SPEAQeasy*; this object included sample quality metrics that were used for exploratory analyses (**Supplementary Methods: Exploratory Data Analysis**).

### Filtering of lowly-expressed genes

Lowly-expressed genes were filtered using filterByExpr() from *edgeR* v3.43.7 [(111)](https://sciwheel.com/work/citation?ids=673952&pre=&suf=&sa=0) in which only genes with at least 15 total reads across all samples and with 10 or more counts in at least *n* samples are retained, where *n* is defined as 70% the size of the smallest sample group. After this step, 16,708 genes (54.86%) were retained for downstream analyses.

### Count normalization

Raw expression counts of the genes in the 33 samples (8 fentanyl and 8 saline Hb samples, and 8 fentanyl and 9 saline Amyg samples) were normalized by trimmed mean of M-values (TMM) [(61)](https://sciwheel.com/work/citation?ids=148215&pre=&suf=&sa=0) using calcNormFactors() from *edgeR* v3.43.7 [(111)](https://sciwheel.com/work/citation?ids=673952&pre=&suf=&sa=0) to compute normalization factors for library size rescaling. *edgeR* cpm() [(111)](https://sciwheel.com/work/citation?ids=673952&pre=&suf=&sa=0) was subsequently used to obtain counts per million (CPM) in a logarithmic scale: approximately log_2_(CPM+0.5).

## Exploratory Data Analysis (EDA)

### Sample Quality Control Analysis (QCA)

Sample-level gene-based quality control (QC) metrics computed by *SPEAQeasy* [(60)](https://sciwheel.com/work/citation?ids=10988515&pre=&suf=&sa=0) on raw counts before gene filtering and count normalization steps ([**Table S3**](#sta_sample_variables_dictionary), [**Table S4**](#sta_sample_metadata_and_QCmetrics)), were compared across the different brain regions, substances, preparation batches, and rat administration sessions. Hb samples presented lower yields of RNA compared to Amyg samples, which in turn decreased their library sizes, number of detected genes, and read mapping rates ([**Figure S2**](#sfi_QC_hab_amyg)). The third RNA extraction batch was performed on additional samples and resulted in more comparable yields with the second higher-yield Amyg batch. Hb and Amyg samples from the third RNA extraction batch presented good RNA amounts and library sizes, and higher mapping rates than their counterparts in the first and second batches, respectively ([**Figure S3**](#sfi_QC_RNA_extraction_batch)). No association was observed between sample quality metrics and total number of fentanyl self-administration sessions ([**Figure S4**](#sfi_QC_total_num_fentanyl_sessions)). Hb and Amyg samples were analyzed separately in subsequent steps.

Low-quality samples were defined through the identification of outlier QC metrics with isOutlier() from *scater* v1.30.1 [(112)](https://sciwheel.com/work/citation?ids=3436659&pre=&suf=&sa=0), which takes as outliers those values that are 3 median-absolute-deviations (MAD) away from the median. For Hb, one of the 16 samples was detected as an outlier for the number of detected genes ([**Figure S5**](#sfi_lowQC_sample_detection)**A**) and for Amyg, 3 of the 17 samples were detected as outliers for mitochondrial mapping rate, concordant mapping rate, or number of detected genes ([**Figure S5**](#sfi_lowQC_sample_detection)**B**); two of the three Amyg outliers were from the third RNA extraction batch. However, these four samples had no additional outlier QC metrics ([**Figure S5**](#sfi_lowQC_sample_detection)) and were not removed but examined further in principal component (PC) plots (**Supplementary Methods: Sample-level gene expression variation and manual QC inspection**). Sample QC metrics are described in [**Table S3**](#sta_sample_variables_dictionary).

### Sample-level gene expression variation and manual QC inspection

Sources of gene expression variation between samples were explored with Principal Component Analysis (PCA) on log-normalized counts of expressed genes. RNA extraction batch appeared as a major driver of gene expression variability in both Hb and Amyg ([**Figure S6**](#sfi_PCA_plots)), and substance had a greater contribution among Amyg samples ([**Figure S6**](#sfi_PCA_plots)**B**). The four previous outlier samples in QC metrics ([**Figure S5**](#sfi_lowQC_sample_detection)) were not outliers on PC plots, although other samples were detected as atypical by PCA ([**Figure S7**](#sfi_PCA_and_QC_boxplots)). Both QC metrics and PCA outlier samples were subjected to manual inspection of all their QC metrics, finding only attenuated differences in the quality of these samples compared to the non-outlier ones, as well as high-quality metrics for these samples relative to the global metrics estimates ([**Figure S7**](#sfi_PCA_and_QC_boxplots)). All Hb and Amyg samples were retained for posterior analyses.

### Gene-level expression variation and covariate selection for DGE

To explore the contributions of sample-level variables on gene expression variation and guide variable selection to model gene expression for DGE analysis, we first computed the percentage of variance of gene expression explained by each covariate individually with getVarianceExplained() from *scater* v1.30.1 [(112)](https://sciwheel.com/work/citation?ids=3436659&pre=&suf=&sa=0). We implemented this analysis taking all Hb and Amyg samples separately ([**Figure S8**](#sfi_covariate_selection_DGE)) and then subsetting to fentanyl-administered samples from each brain region ([**Figure S8**](#sfi_covariate_selection_DGE)), as additional DGE analyses were performed on fentanyl-administered samples only (see further below and **Supplementary Methods: Differential Gene Expression analysis**).

Second, for the same sample groups, we performed pairwise Canonical Correlation Analysis (CCA) with canCorPairs() of *variancePartition* v1.32.5 [(113)](https://sciwheel.com/work/citation?ids=3280148&pre=&suf=&sa=0) to identify pairs of correlated variables ([**Figure S8**](#sfi_covariate_selection_DGE)). To remove redundant and minority contributing variables and to avoid unmasking true drivers of variation, models for DGE between fentanyl vs. saline administration and for rat behavior in Hb and Amyg were defined by discarding:

i) variables highly correlated with “substance” (fentanyl vs. saline; [**Figure S8**](#sfi_covariate_selection_DGE)) or “behavioral covariates” (i.e. the slope of fentanyl infusions across each LgA self-administration session 1st hour, total fentanyl intake across LgA self-administration sessions, and last LgA self-administration session fentanyl intake; [**Figure S8**](#sfi_covariate_selection_DGE)), given these were not true confounders;

ii) variables highly correlated with the RNA extraction batch previously shown to affect samples’ quality metrics ([**Figure S3**](#sfi_QC_RNA_extraction_batch)) and explain high percentages of expression variation in several genes ([**Figure S8**](#sfi_covariate_selection_DGE));

iii) variables highly correlated with the total number of fentanyl sessions and this variable itself, as it didn’t impact on sample QC metrics ([**Figure S4**](#sfi_QC_total_num_fentanyl_sessions)) and had minor contributions on gene expression differences ([**Figure S8**](#sfi_covariate_selection_DGE)), and

iv) for other pairs of correlated variables, we only kept the one with the highest percentages of gene expression variance explained obtained with getVarianceExplained() ([**Figure S8**](#sfi_covariate_selection_DGE)).

Then, the fraction of variation in the expression of each gene attributable to each included variable was assessed with a variance partition analysis using fitExtractVarPartModel() from *variancePartition* [(113)](https://sciwheel.com/work/citation?ids=3280148&pre=&suf=&sa=0), jointly accounting for the contributions of the rest of selected variables to confirm their impacts on gene expression and suitability for DGE ([**Figure S8**](#sfi_covariate_selection_DGE)). Sample variables are defined in [**Table S3**](#sta_sample_variables_dictionary).

## Differential Gene Expression (DGE) analysis

We assessed DGE for substance and behavior under the empirical Bayes framework of *limma-voom* v3.58.1 [(114)](https://sciwheel.com/work/citation?ids=148089&pre=&suf=&sa=0) pipeline, fitting a linear model to the expression of each gene including as covariates the sample variables that were not correlated between them and that explained high percentages of global gene expression variance (**Supplementary Methods: Exploratory Data Analysis**). The gene-wise *p*-values of the resulting moderated *t*-statistics were adjusted for multiple testing using the Benjamini and Hochberg’s (BH) procedure that controls the false discovery rate (FDR) [(115)](https://sciwheel.com/work/citation?ids=6279401&pre=&suf=&sa=0). Genes with FDR adjusted *p*-values below 0.05 were considered as DEGs.

The following were the specific DGE analyses performed and the covariates included to model gene expression in each.

### DGE for substance (fentanyl vs. saline) in Hb and Amyg samples

Differential expression between fentanyl vs. saline administration ($Substance$) was tested for each gene $i$ by modeling its gene expression ($y_{ij}$) across the $j$ samples in Hb as:

$$y_{ij}=\beta_{0i}+\beta_{1i}Substance_{j} +\beta_{2i}Batch_{-}RNA_{-}extraction_{j} +\beta_{3i}concordMapRate_{j}+\beta_{4i}RIN_{j} + \varepsilon_{ij}$$

And in Amyg:

$$y_{ij}=\beta_{0i}+\beta_{1i}Substance_{j} +\beta_{2i}Batch_{-}RNA_{-}extraction_{j} +\beta_{3i}Batch_{-}li{b_{-}prep}_{j}+\beta_{4i}overallMapRate_{j}+\beta_{5i}RIN_{j} + \varepsilon_{ij}$$

Where the $\beta_{i}$’s correspond to the estimated model coefficients of the included covariates for the $i$-th gene and $\varepsilon_{ij}$ the observational-level error term.

Similarly, DGE for behavioral covariates of the rats that self-administered fentanyl was assessed under the following models in each brain region. These behavioral covariates were: the slope of fentanyl intake in each session first hour ($First\_hour_{-}infusion_{-}slope$), total fentanyl intake ($Total_{-}Intake$), and last session fentanyl intake ($Last_{-}Session_{-}Intake$). Covariates are defined in [**Table S3**](#sta_sample_variables_dictionary).

### DGE for 1st hour infusion slope in Hb and Amyg fentanyl samples

- In Hb:

$$y_{ij}=\beta_{0i}+\beta_{1i}First\_hour_{-}infusion_{-}slope_{j} +\beta_{2i}RIN_{j} +\beta_{3i}RNA_{-}concentration_{j}+\beta_{4i}mitoRate_{j} + \varepsilon_{ij}$$

- In Amyg:

$$y_{ij}=\beta_{0i}+\beta_{1i}First\_hour_{-}infusion_{-}slope_{j} +\beta_{2i}RIN_{j} +\beta_{3i}mitoRate_{j} + \varepsilon_{ij}$$

### DGE for total drug intake in Hb and Amyg fentanyl samples

- In Hb:

$$y_{ij}=\beta_{0i}+\beta_{1i}Total_{-}Intake_{j} +\beta_{2i}RIN_{j} +\beta_{3i}RNA_{-}concentration_{j}+\beta_{4i}overallMapRate_{j} + \varepsilon_{ij}$$

- In Amyg:

$$y_{ij}=\beta_{0i}+\beta_{1i}Total_{-}Intake_{j} +\beta_{2i}RIN_{j} +\beta_{3i}mitoRate_{j} + \varepsilon_{ij}$$

### DGE for last session intake in Hb and Amyg fentanyl samples

- In Hb:

$$y_{ij}=\beta_{0i}+\beta_{1i}Last_{-}Session_{-}Intake_{j} +\beta_{2i}RIN_{j} +\beta_{3i}RNA_{-}concentration_{j}+\beta_{4i}mitoRate_{j} + \varepsilon_{ij}$$

- In Amyg:

$$y_{ij}=\beta_{0i}+\beta_{1i}Last_{-}Session_{-}Intake_{j} +\beta_{2i}RIN_{j} +\beta_{3i}totalAssignedGene_{j} +\beta_{4i}concordMapRate_{j}+\varepsilon_{ij}$$

## Cross-region enrichment analysis

Concordance in the gene expression effects of fentanyl across Hb and Amyg was examined through a Rank-Rank Hypergeometric Overlap analysis (RRHO), ranking genes by *p*-value for DGE and effect directionality, as implemented in *RRHO2* package v1.0 [(62)](https://sciwheel.com/work/citation?ids=5484838&pre=&suf=&sa=0). This method evaluated the significance of the overlap between the top *p* most significant up/down-regulated genes in Hb and the top *q* most significant up/down-regulated genes in Amyg. By testing enrichment across all values *p*,*q* = 1, …, *N*, where *N* is the number of genes tested for DGE in both Hb and Amyg, this method allows comparison of fentanyl’s transcriptional signatures between regions across the entire transcriptome, without restricting enrichment testing to DEGs defined by *p*-value thresholding.

## Functional enrichment analysis

Gene sets annotated in Gene Ontology (GO) [(116)](https://sciwheel.com/work/citation?ids=963677&pre=&suf=&sa=0) terms and Kyoto Encyclopedia of Genes and Genomes (KEGG) [(117)](https://sciwheel.com/work/citation?ids=718914&pre=&suf=&sa=0) pathways that were significantly overrepresented among our Hb and Amyg DEGs for substance were found with hypergeometric tests implemented in *clusterProfiler* v4.10.0 [(118)](https://sciwheel.com/work/citation?ids=1509330&pre=&suf=&sa=0) using compareCluster(). The complete set of expressed genes that were assessed for DGE and with available Entrez gene IDs (n=14,066 genes) was taken as the background gene set. The obtained gene set *p*-values were FDR-adjusted [(115)](https://sciwheel.com/work/citation?ids=6279401&pre=&suf=&sa=0).

## Cell type enrichment analysis

Marker genes for 1) main cell types and and inhibitory neuronal subtypes in control rat Amyg [(52)](https://sciwheel.com/work/citation?ids=15523346&pre=&suf=&sa=0), 2) cell types at fine and broad resolutions in the human Hb-enriched epithalamus [(50)](https://sciwheel.com/work/citation?ids=18372257&pre=&suf=&sa=0) and human Amyg [(51)](https://sciwheel.com/work/citation?ids=14404156&pre=&suf=&sa=0) of neurotypical control donors donors, and 3) all and the Hb neuronal cell subpopulations in the Hb complex of control mice [(49)](https://sciwheel.com/work/citation?ids=8601460&pre=&suf=&sa=0) were obtained using normalized and filtered sn/scRNA-seq data. Markers were found implementing the *MeanRatio* method of *DeconvoBuddies* v0.99.0 [(63)](https://sciwheel.com/work/citation?ids=17727289&pre=&suf=&sa=0). Cell types with less than 10 cells were discarded from marker finding analysis.

Briefly, *MeanRatio* defines as cell type markers those genes with the greatest mean expression in the target cell type compared to any other cell type, computing for each gene the ratio between the mean expression in the target cell type, and the highest mean expression among the non-target cell types (i.e. the *MeanRatio*) [(63)](https://sciwheel.com/work/citation?ids=17727289&pre=&suf=&sa=0). The top 100 or 50 (human Hb [(50)](https://sciwheel.com/work/citation?ids=18372257&pre=&suf=&sa=0)) marker genes with *MeanRatios* >1 were used ([**Table S11**](#sta_MeanRatio_markers_top100_hab_mouse), [**Table S12**](#sta_MeanRatio_markers_top50_hab_human), [**Table S13**](#sta_MeanRatio_markers_top100_amy_rat), [**Table S14**](#sta_MeanRatio_markers_top100_amy_human)).

Then, rat orthologs of human and mouse cell type marker genes were obtained using *biomaRt* v2.56.1 [(66)](https://sciwheel.com/work/citation?ids=252618&pre=&suf=&sa=0) under the GRCh38 and GRCm39 genome versions for human and mouse, respectively. The sets of cell type-specific markers in rat were assessed for their enrichment among all, up-, and down-regulated fentanyl vs. saline DEGs in rat Hb and Amyg based on the one-sided Fisher’s exact test. Expressed genes assessed for DGE were considered the background gene set (n=16,708 genes).

## Generalized Gene-Set Analysis of GWAS data

*MAGMA* v1.10 [(64)](https://sciwheel.com/work/citation?ids=1234481&pre=&suf=&sa=0) was run to assess the joint association of genes in each set of all, up-, and down-regulated Hb and Amyg DEGs with multiple SUDs and psychiatric disorders. Briefly, *MAGMA* was provided as input the summary statistics of genome-wide human Single Nucleotide Polymorphisms (SNPs) from six GWASes: schizophrenia (SCZ) [(58)](https://sciwheel.com/work/citation?ids=12782344&pre=&suf=&sa=0), Panic Disorder (PD) [(57)](https://sciwheel.com/work/citation?ids=7796030&pre=&suf=&sa=0), Opioid Use Disorder (OUD) [(54)](https://sciwheel.com/work/citation?ids=15008448&pre=&suf=&sa=0), Substance Use Disorder (SUD) [(53)](https://sciwheel.com/work/citation?ids=14593338&pre=&suf=&sa=0), and Major Depressive Disorder (MDD) [(55,56)](https://sciwheel.com/work/citation?ids=6375671,11142583&pre=&pre=&suf=&suf=&sa=0,0) .

For each GWAS, autosomal SNPs were first mapped onto human genes based on the same human genome reference build used in each study (either GRCh37 or GRCh38; hg19 or hg38). For the gene-level analysis SNP *p*-values were used to compute gene-level *p*-values for their association with the phenotype through the SNP-wise mean Z-statistics method. Given that most of the ancestry composition of all examined GWASes was of European Ancestry, the 1000 Genomes European Phase 3 panel [(65)](https://sciwheel.com/work/citation?ids=790619&pre=&suf=&sa=0) was used as the reference dataset to account for linkage disequilibrium between SNPs.

For gene-set analysis, the human orthologs of rat Hb and Amyg DEGs from each set (all, up-, and down-regulated) were first retrieved from Ensembl release 112 (mRatBN7.2) [(119)](https://sciwheel.com/work/citation?ids=17292735&pre=&suf=&sa=0) using *biomaRt* v2.61.1 [(66)](https://sciwheel.com/work/citation?ids=252618&pre=&suf=&sa=0). Then a competitive positive one-sided gene-set analysis was implemented on such sets of human orthologs to assess their association with the GWAS phenotype based on gene-level associations.

## Cross-study DEG comparison

To compare transcriptional signatures across studies, differentially expressed gene (DE) lists were obtained from the supplementary materials of previously published studies [(44)](https://sciwheel.com/work/citation?ids=16560965&pre=&suf=&sa=0). Gene-level overlap was determined by direct matching (intersection) of gene symbols between datasets. Additionally, we ensured significance criteria (adjusted p-value < 0.05 in each dataset).

## Software

All the analysis code is available at <https://github.com/LieberInstitute/fentanyl_rat_hb_amy> [(104)](https://sciwheel.com/work/citation?ids=18392537&pre=&suf=&sa=0). Analyses were performed using R versions 4.3.2 to 4.4.0 [(120)](https://sciwheel.com/work/citation?ids=15967786&pre=&suf=&sa=0) with Bioconductor versions 3.17 to 3.19 [(110)](https://sciwheel.com/work/citation?ids=111791&pre=&suf=&sa=0). Visualizations were made using *ggplot2* v3.4.4 and v3.5.1 [(121)](https://sciwheel.com/work/citation?ids=12688379&pre=&suf=&sa=0).

# Supplementary Figures


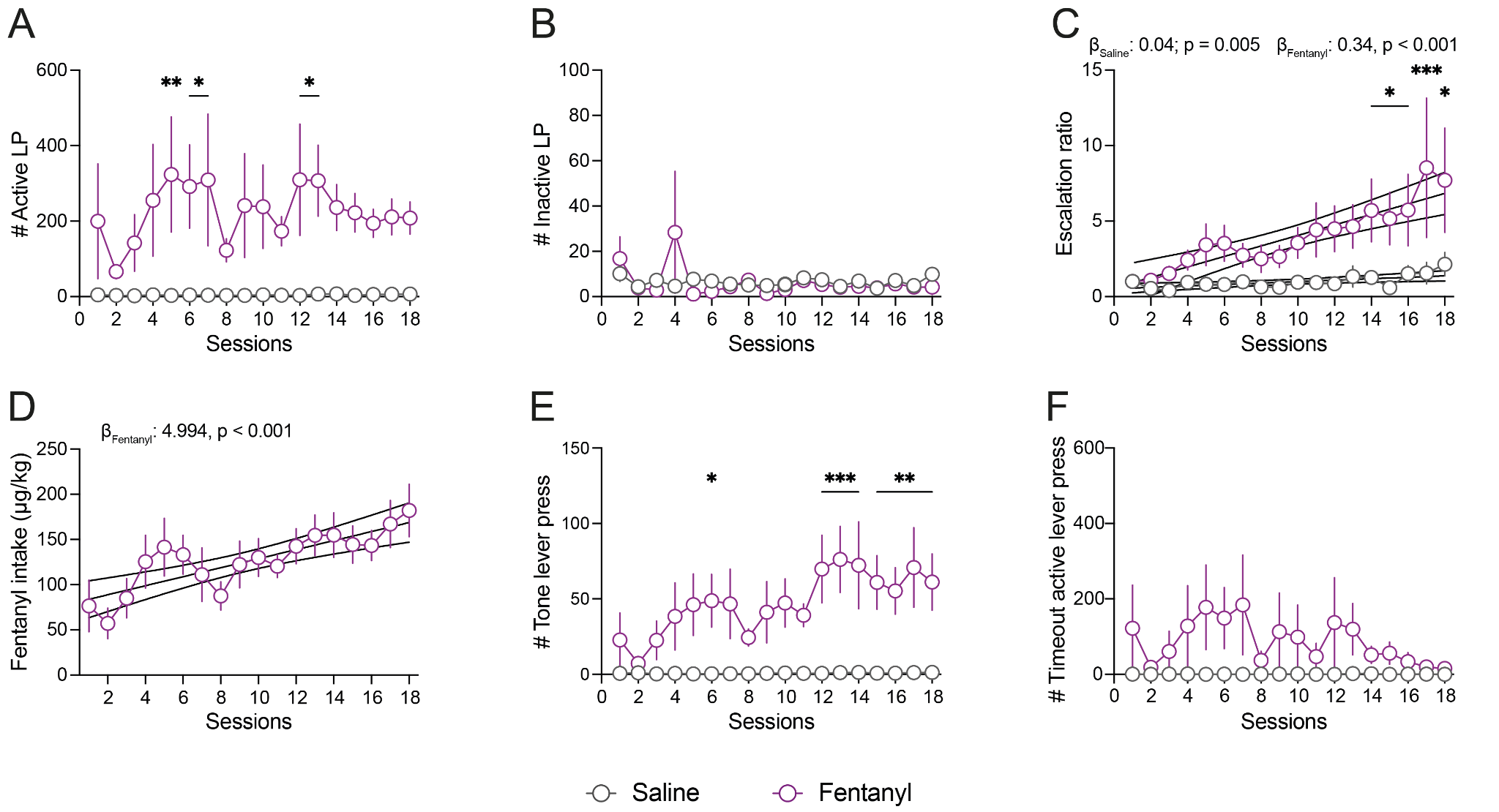


[**Figure S1**](#sfigu_behav_metrics)**:** **Additional behavior metrics from LgA sessions.** Mean number of total (**A**) active (mixed-effects model (REML), substance effect: F_1,17_ = 10.97, p = 0.0041; session effect: F_17,273_ = 1.44, p = 0.118; substance x session interaction: F_17,273_ = 1.4, p = 0.136; Šídák post hoc comparisons) and (**B**) inactive lever presses per LgA session for saline and fentanyl rats (mixed-effects model (REML), substance effect: F_1,17_ = 0.010, p = 0.918; session effect: F_17,273_= 1.26, p = 0.214; substance x session interaction: F_17,273_ = 1.19, p = 0.265). (**C**) Escalation ratio, an alternative metric to quantify infusion escalation across sessions. This ratio is calculated by normalizing each rat’s LgA infusion counts relative to their infusion count on the first LgA session (mixed-effects model (REML), substance effect: F_1,17_ = 8.77, p = 0.0088; session effect: F_17,273_ = 4.69, p < 0.001; substance x session interaction: F_17,273_ = 2.81, p < 0.001; Šídák post hoc comparisons). (**D**) Fentanyl intake (µg/kg) per LgA session. (**E**) Number of active lever presses performed during the 2.8 second infusion and tone presentation period (mixed-effects model (REML), substance effect: F_1,17_ = 19.4, p < 0.001; session effect: F_17,273_ = 2.91, p < 0.001; substance x session interaction: F_17,273_ = 2.82, p < 0.001; Šídák post hoc comparisons). (**F**) Number of active lever presses performed during the 20 second timeout period (mixed-effects model (REML), substance effect: F_1,17_ = 3.06, p = 0.098; session effect: F_17,273_ = 1.41, p = 0.131; substance x session interaction: F_17,273_ = 1.41, p = 0.129). Data shown as mean across rats ± SEM. Black lines represent linear regression with 95% confidence intervals. Saline: n = 11 rats; Fentanyl: n = 8 rats. * denotes p < 0.05; ** denotes p < 0.01; *** denotes p < 0.001.


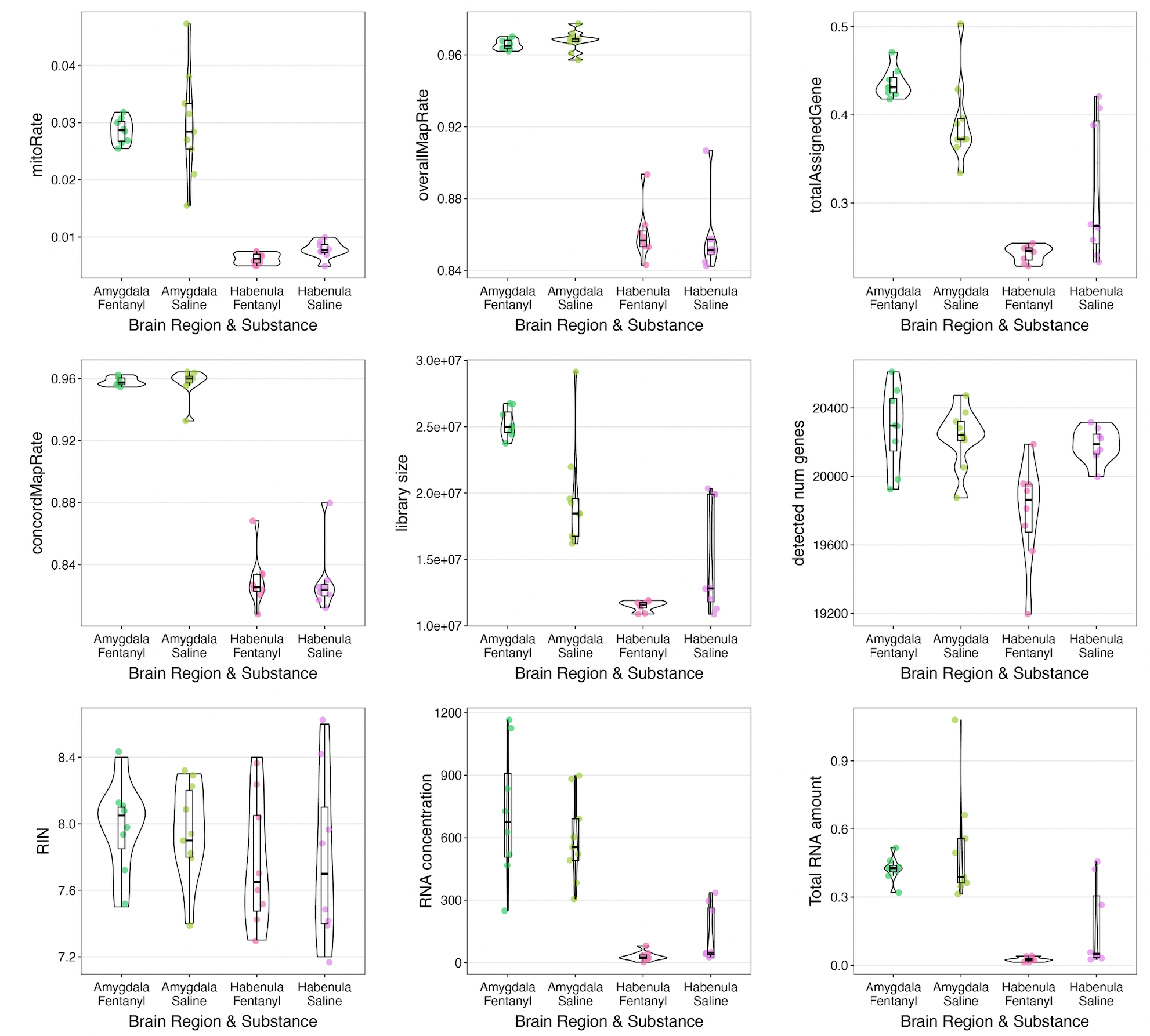


[**Figure S2**](#sfigu_QC_hab_amyg)**: Quality control metrics for Hb and Amyg samples.** Comparison of the QC metrics examined in this study for habenula and amygdala fentanyl and saline samples. Note that different Illumina library preparation kits were used for each brain region, thus confounding brain region and kit differences, which motivated independent analyses for each brain region. See [**Table S3**](#sta_sample_variables_dictionary) for the description of these QC metrics.


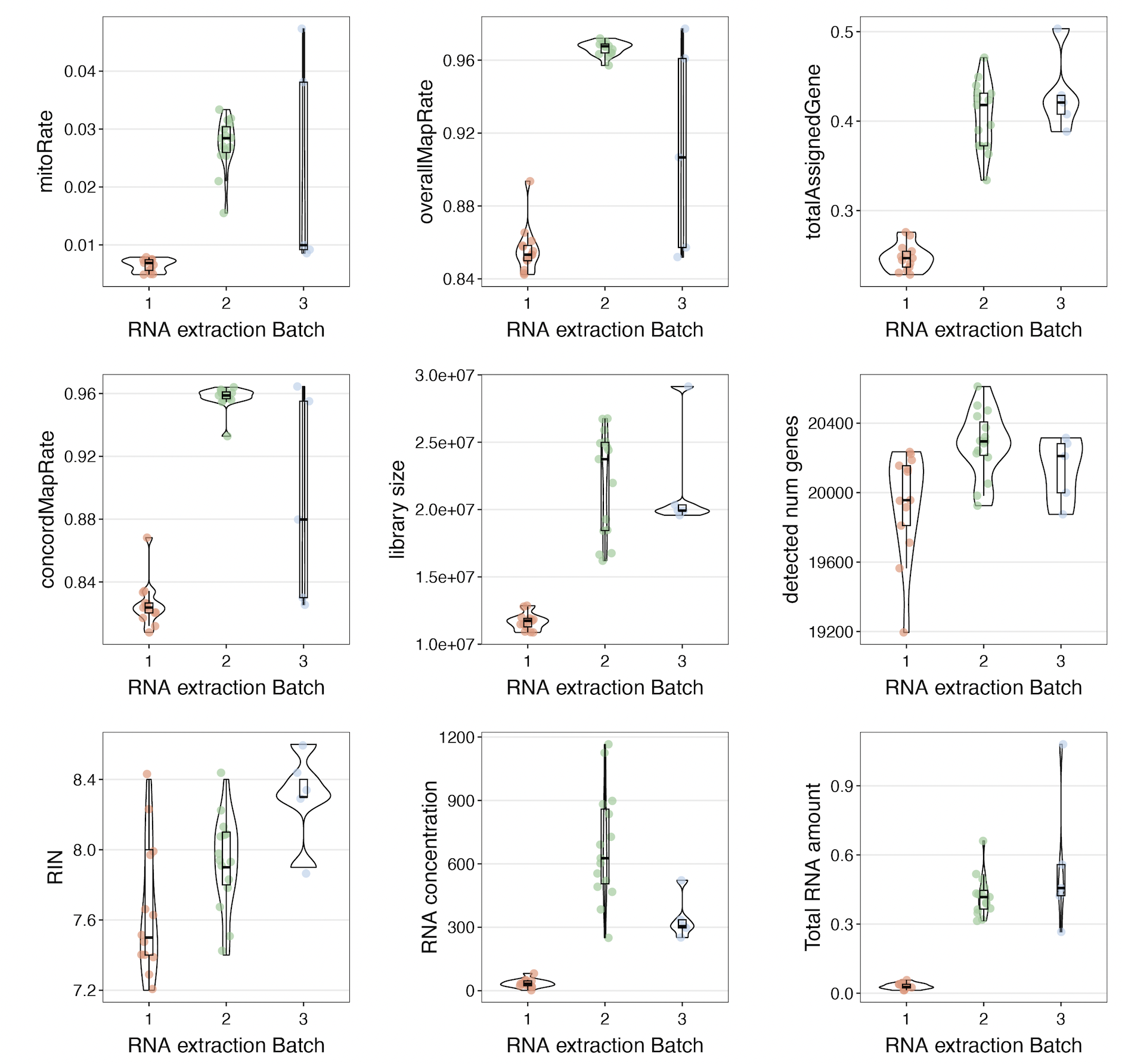


[**Figure S3**](#sfigu_QC_RNA_extraction_batch)**: Quality control metrics for samples across RNA extraction batches.** Comparison of QC metrics of samples from the first (only Hb samples), second (only Amyg samples), and third batch for RNA extraction (additional Hb and Amyg samples). See [**Table S3**](#sta_sample_variables_dictionary) for the description of these QC metrics.


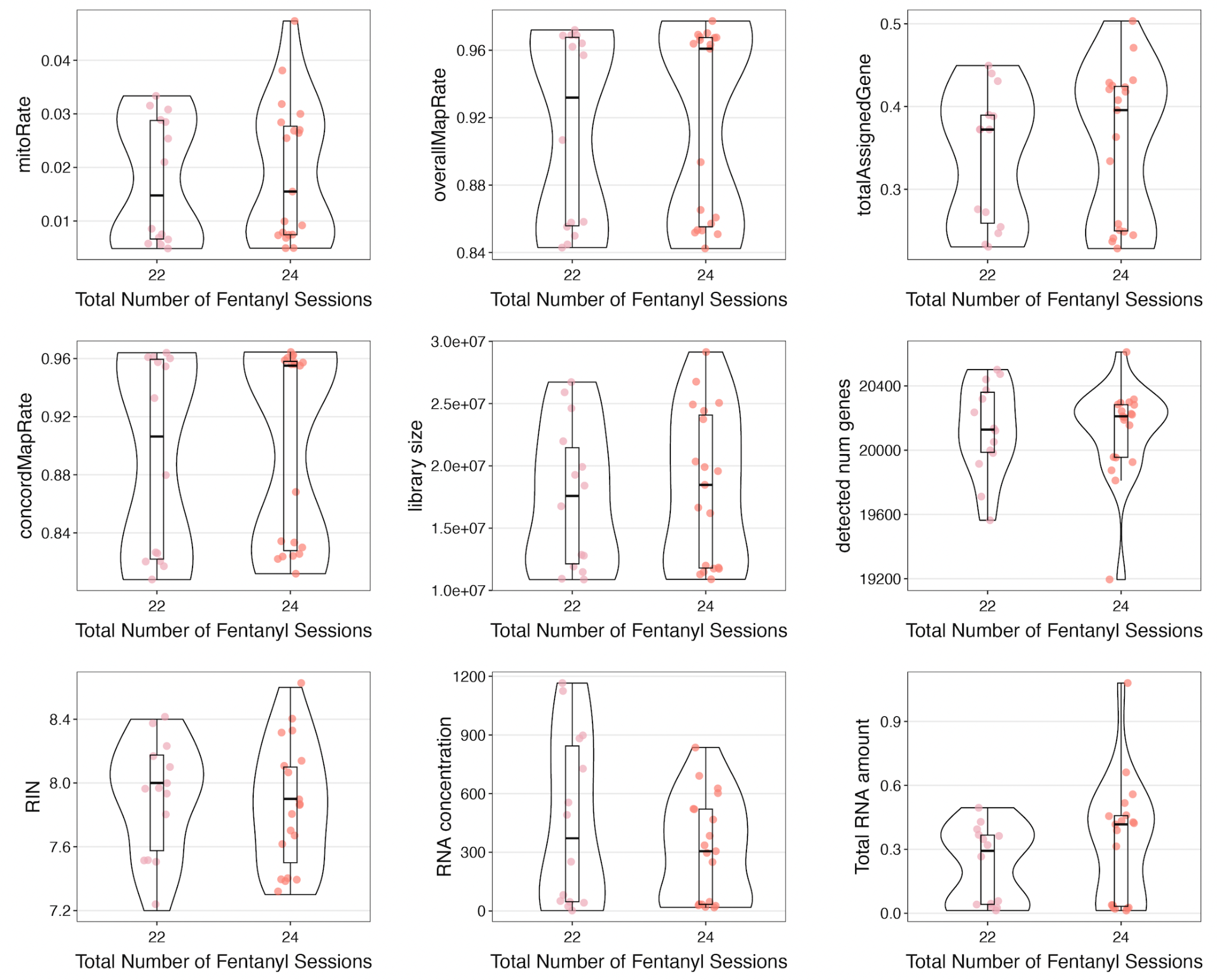


[**Figure S4**](#sfigu_QC_total_num_fentanyl_sessions)**: Quality control metrics for samples across total number of self-administration sessions.** Comparison of QC metrics for (Hb and Amyg) samples from rats who had 22 and 24 total (fentanyl or saline) self-administration sessions. See [**Table S3**](#sta_sample_variables_dictionary) for the description of these QC metrics.


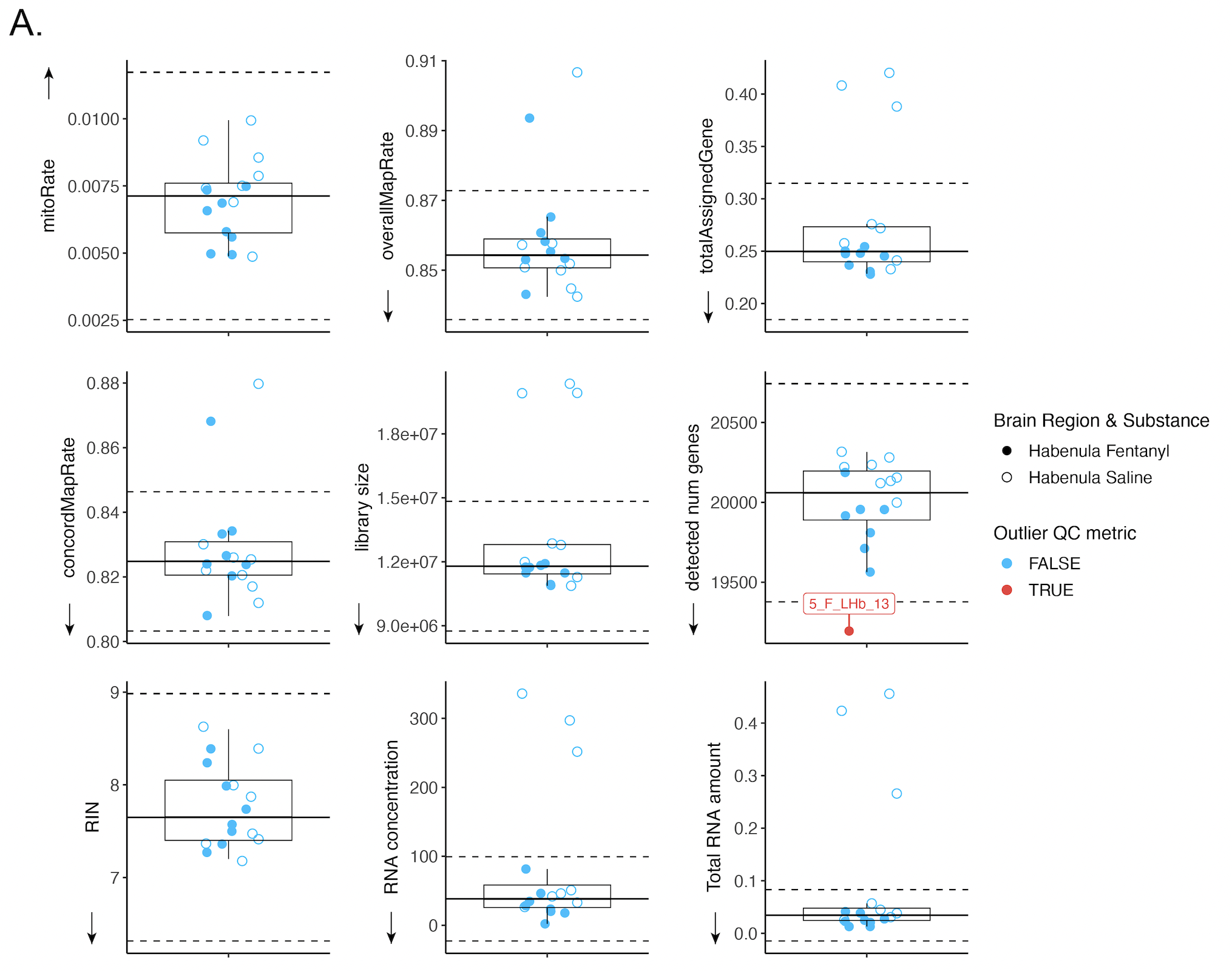


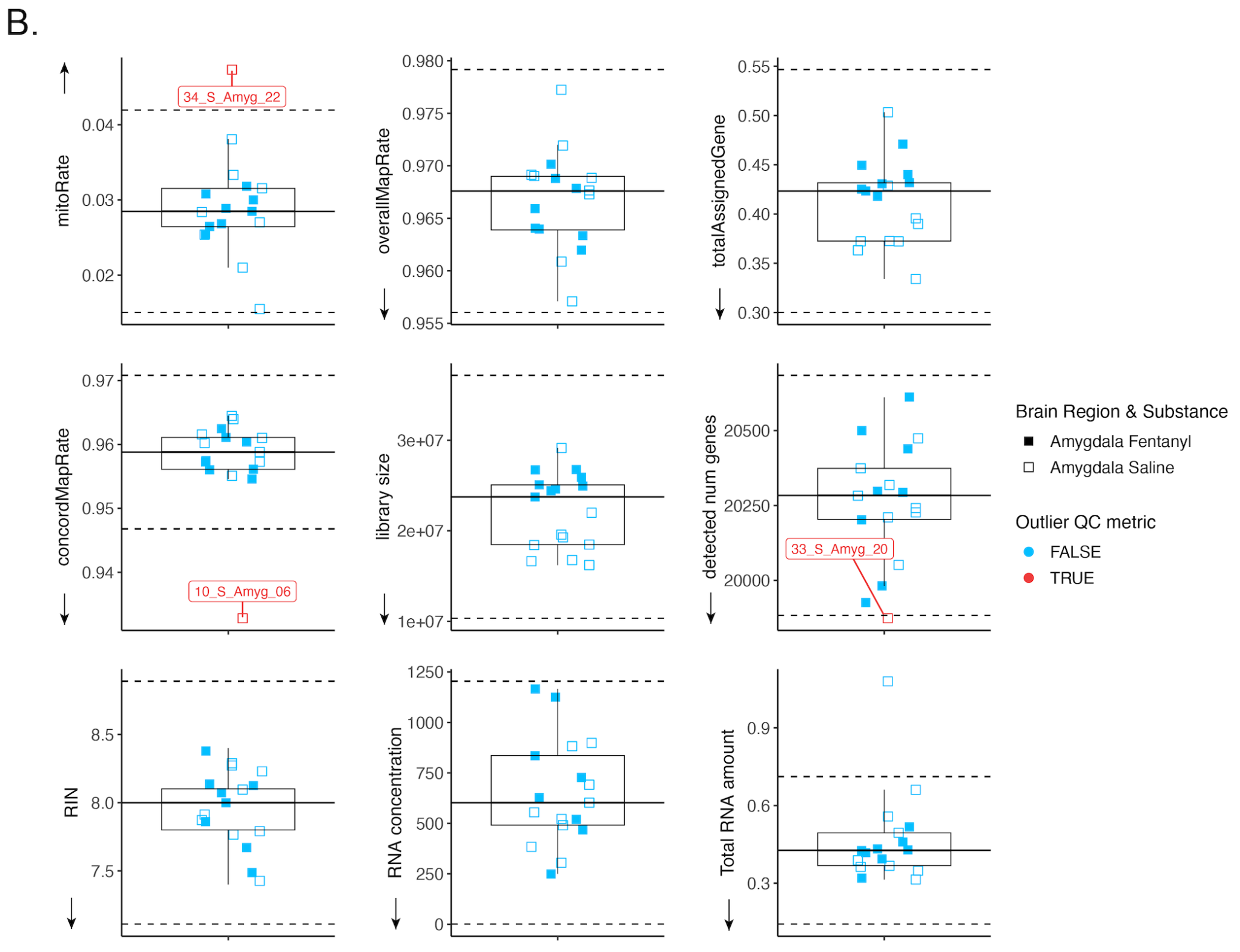


[**Figure S5**](#sfigu_lowQC_sample_detection)**: Low-quality sample identification.** Detection of low-quality metrics for (**A**) Hb and (**B**) Amyg fentanyl (filled circles/squares) and saline (empty circles/squares) samples. QC metric outliers (in red) were identified as those being 3 median-absolute-deviations (MAD; dotted lines) away from the median (solid line). Only lower outliers were considered poor-quality for all QC metrics except mitoRate, for which higher outliers were considered instead (indicated by arrows). Samples with outlier QC metrics are labeled and were subjected to further evaluation in downstream analyses ([**Figure S7**](#sfi_PCA_and_QC_boxplots)). See [**Table S3**](#sta_sample_variables_dictionary) for the description of these QC metrics.


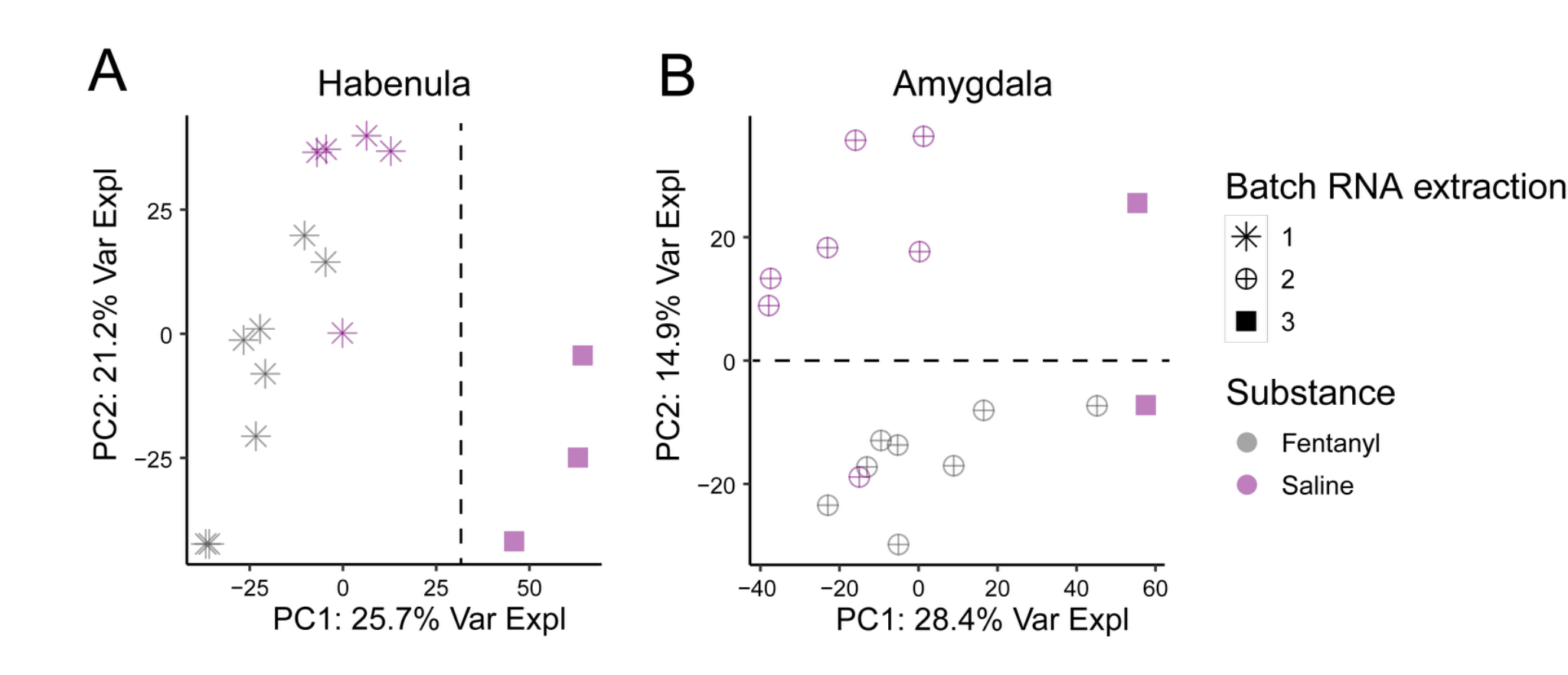


[**Figure S6**](#sfigu_PCA_plots)**: Principal Component Analysis.** PC1 vs. PC2 for gene expression in (**A**) Hb and (**B**) Amyg samples. Percentages of variance explained by each PC are indicated on the axes. Samples are shaped by RNA extraction batch and colored by substance.


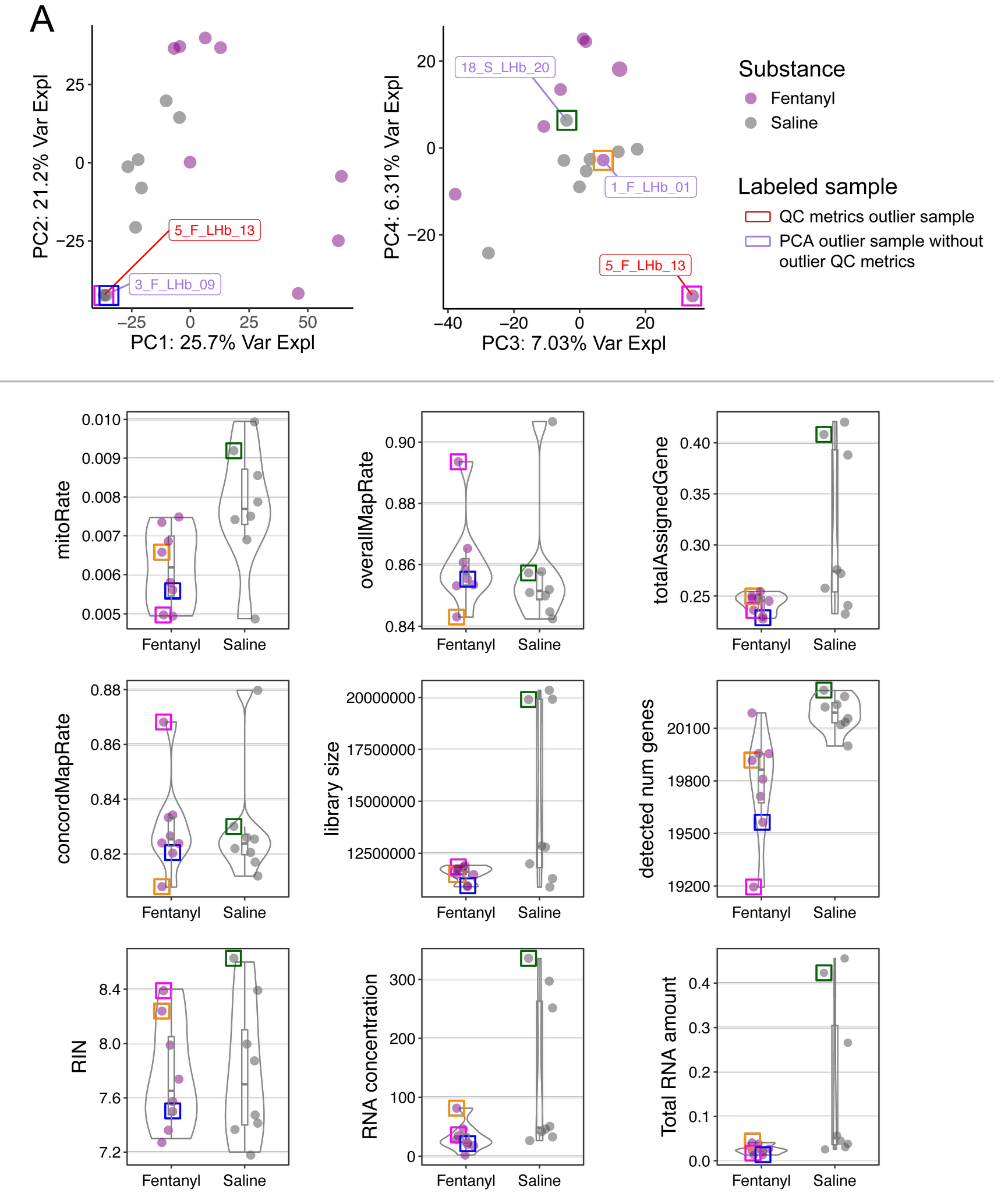


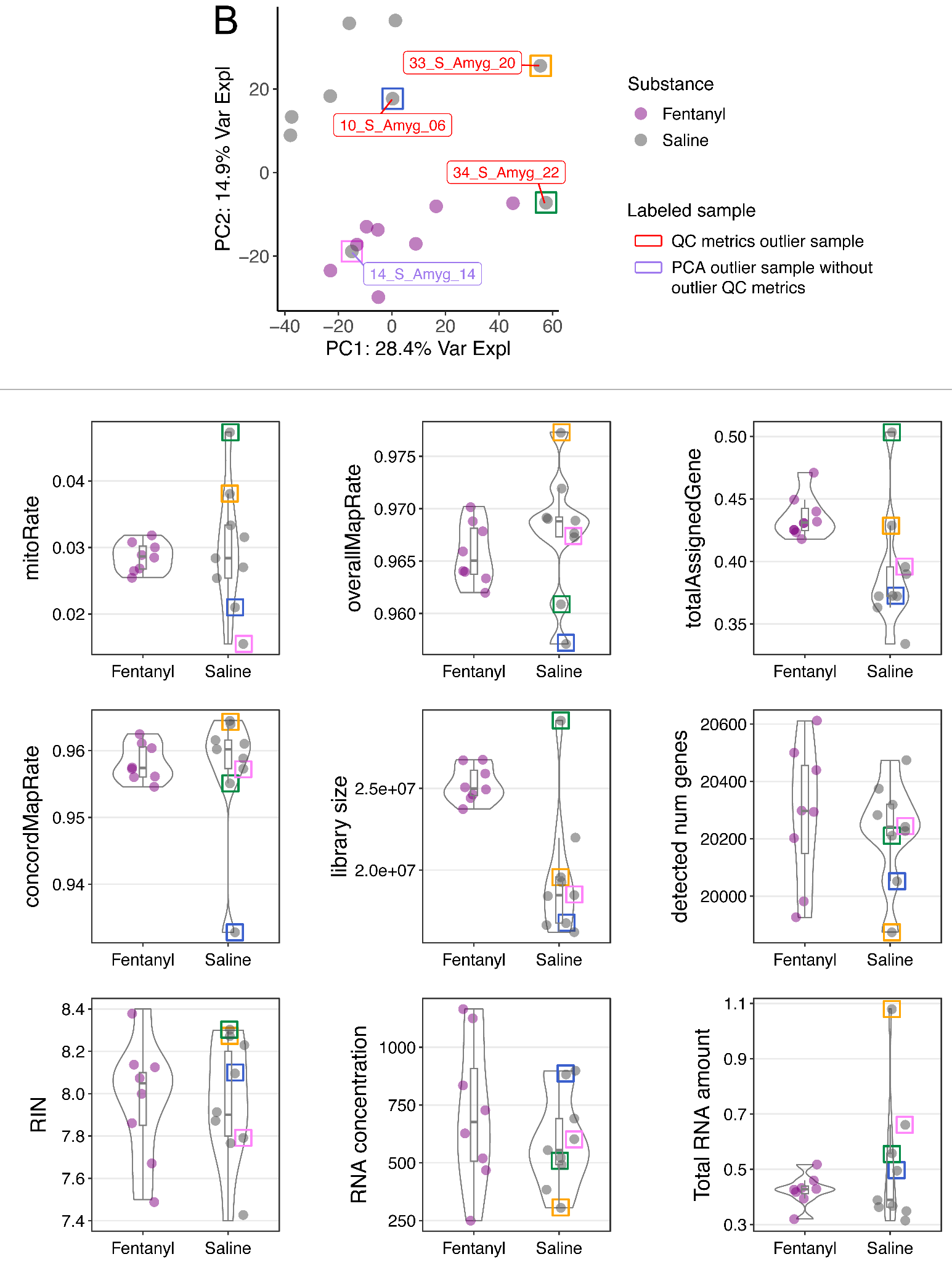


[**Figure S7**](#sfigu_PCA_and_QC_boxplots)**: Manual sample quality examination based on PCA.** PCx vs. PCy (top) for (**A**) Hb and (**B**) Amyg samples. QC metrics outlier samples are labeled in red (see [**Figure S5**](#sfi_lowQC_sample_detection)); samples segregated from the rest in each PC plot, as well as fentanyl and saline samples closer to samples from the other substance group were considered PCA outlier samples and are labeled in purple. The percentage of variance explained by each PC is shown on axis labels. For both, QC metrics and PCA outlier samples, all their QC metrics were reexamined (bottom box plots); different colored squares indicate the different outlier samples. Samples in all plots are colored by substance. See [**Table S3**](#sta_sample_variables_dictionary) for the description of these QC metrics.


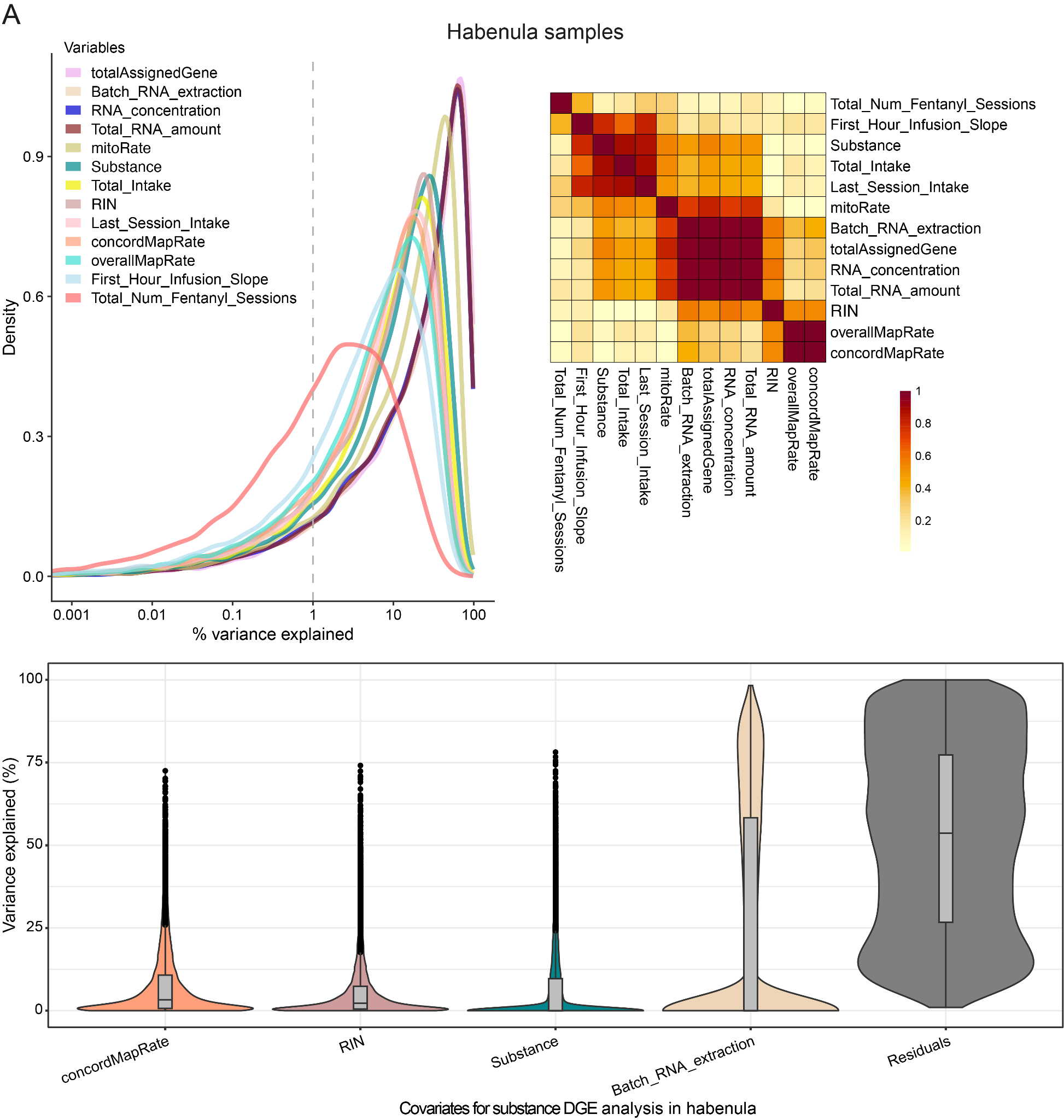


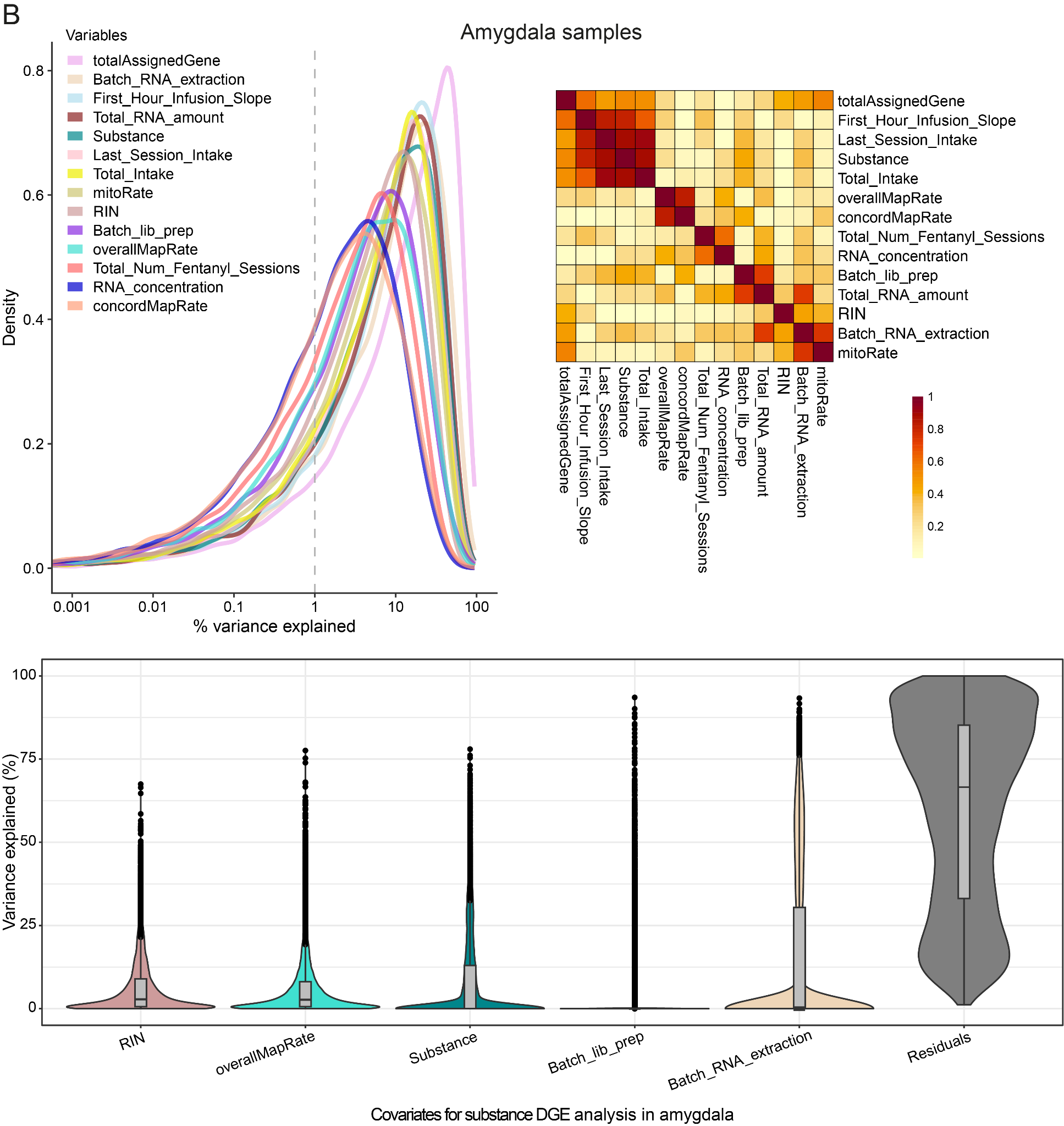


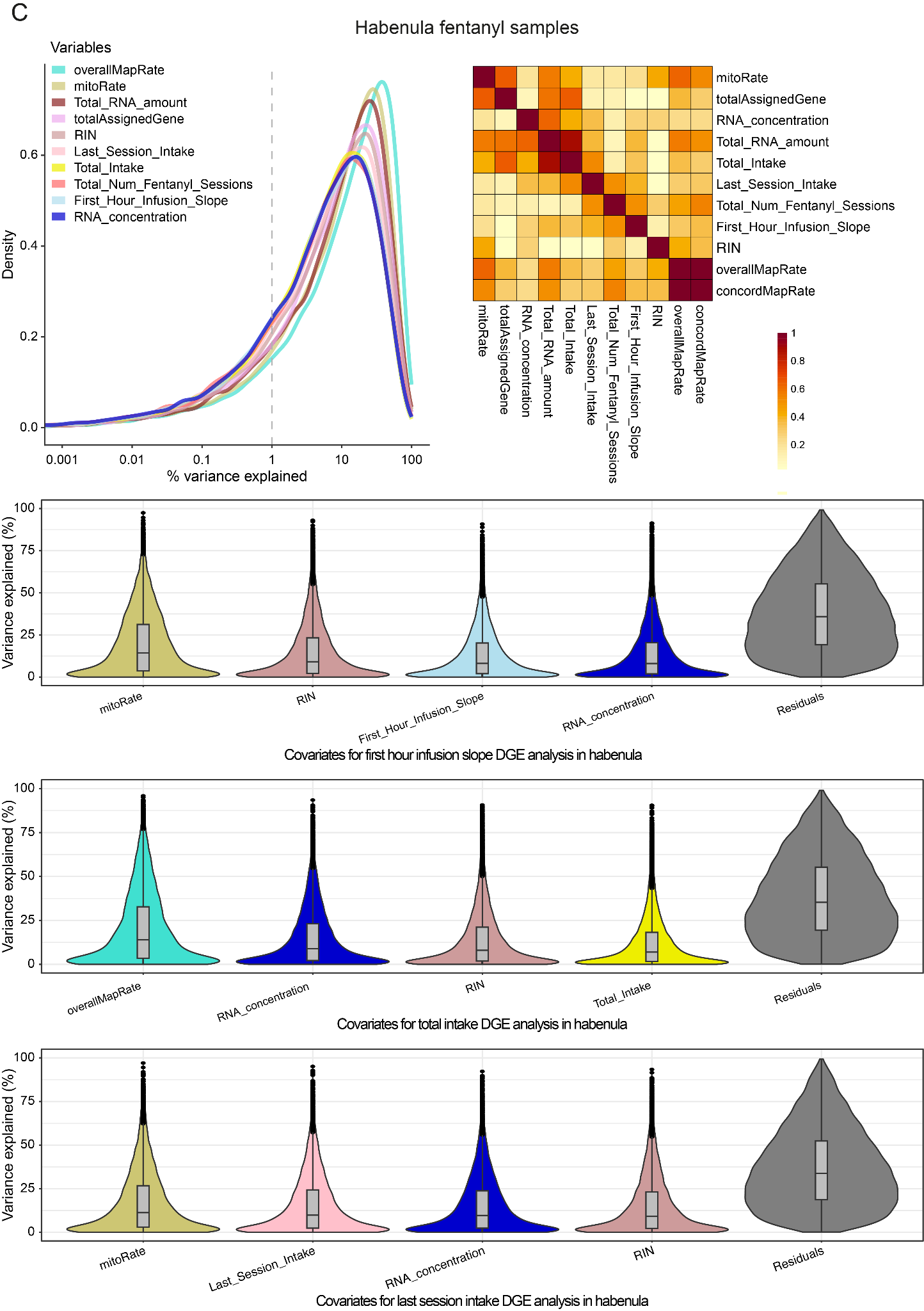


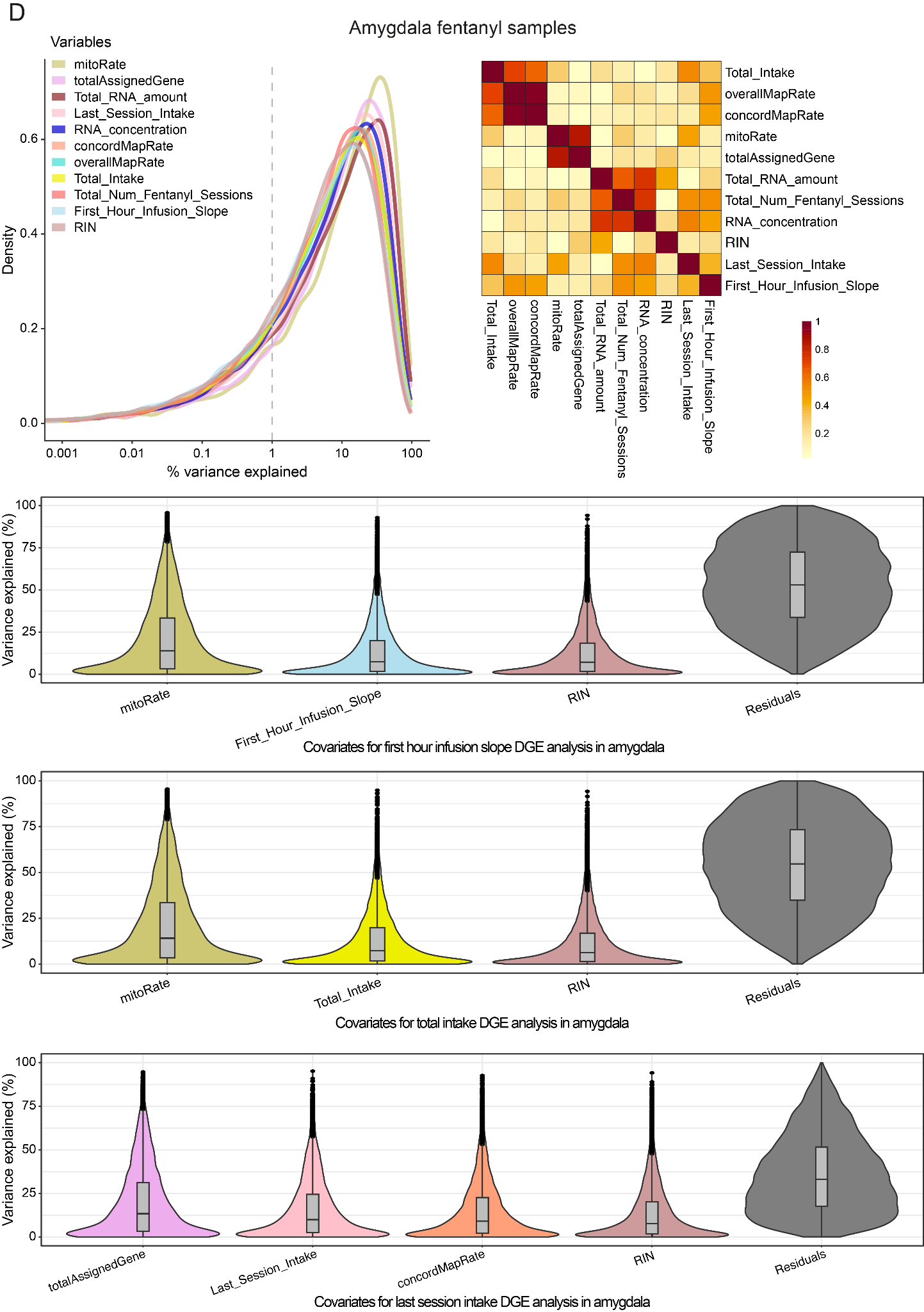


[**Figure S8**](#sfigu_covariate_selection_DGE)**: Sample-level covariate selection for DGE analysis.** Gene expression variance partition analysis in **A.** Hb (all rats), **B.** Amyg (all rats), **C.** Hb (fentanyl rats only), and **D.** Amyg (fentanyl rats only). Top left: density plot for the percentages of variance explained in the expression of each gene by each sample-level variable. Top right: canonical correlation between each pair of variables. Variables included in the models for DGE analyses (A-B. for substance, and C-D. for rat behavioral traits) were selected based on their contributions to gene expression variance and correlations with other variables. Bottom: percentage of variance in the expression of each gene explained by each variable included in the DGE model, considering all other included variables in the model (x-axis); variables are ordered by decreasing median percentage of variance explained. Related to [**Figure 2**](#fig_DEG). See [**Table S3**](#sta_sample_variables_dictionary) for the description of these variables and QC metrics.

#
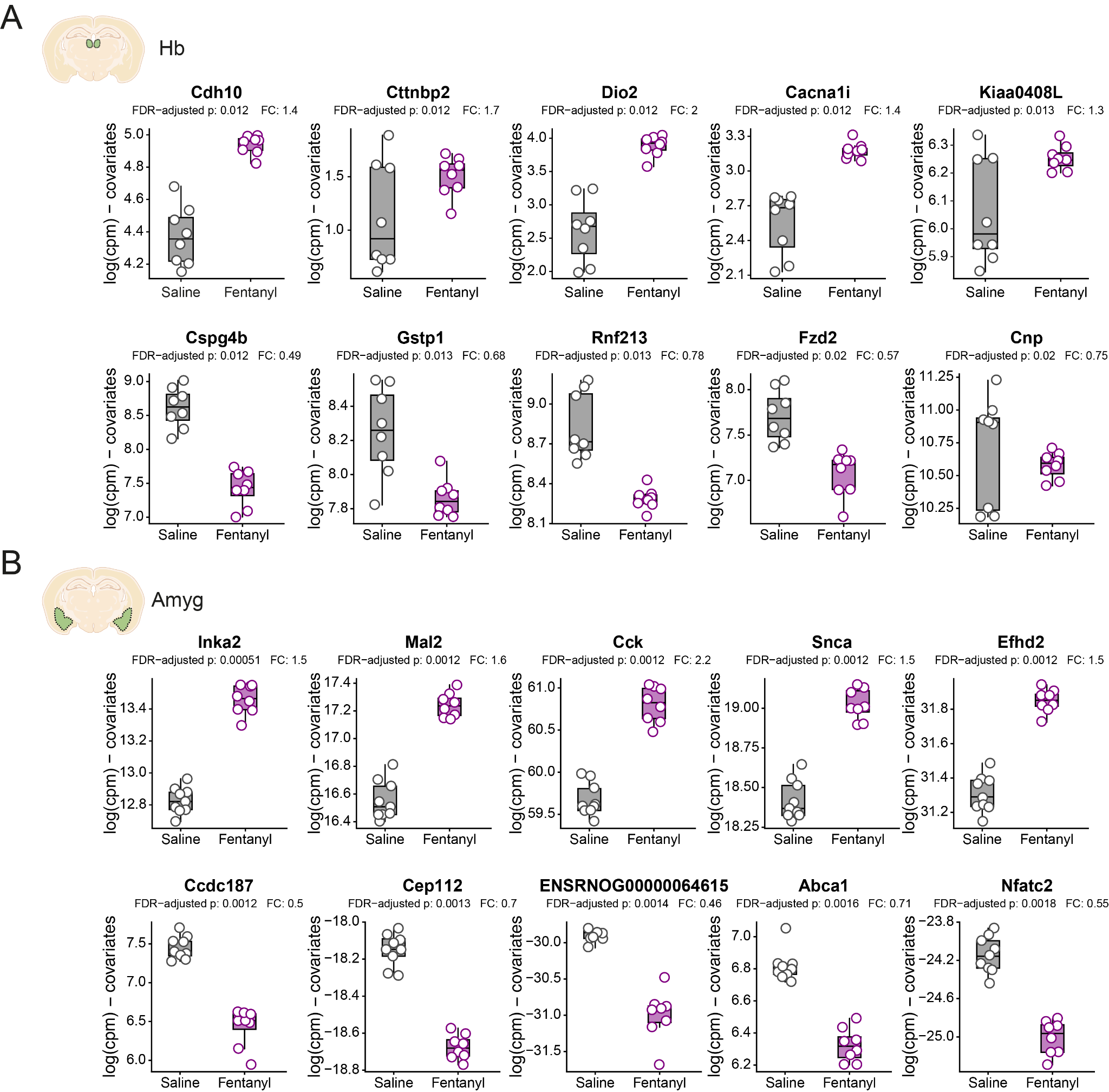


[**Figure S9**](#sfigu_DEG_boxplots)**: Top 5 differentially expressed genes in Hb and Amyg following chronic LgA fentanyl self-administration.** (**A-B**) Box plots showing the expression of the top five up- and down-regulated DEGs for fentanyl vs. saline in Hb (**A**) and Amyg (**B**). Gene expression is given in log_2_(CPM) after regressing out covariates. FDR-adjusted p-value and fold change (FC) are shown for each gene. Boxes extend from the 25^th^ to 75^th^ percentiles; lines within the boxes represent the median; whiskers indicate the minimum and maximum values, superimposed with individual rat data points. Fentanyl Hb n = 8; Saline Hb n = 8; Fentanyl Amyg n = 8; Saline Amyg n = 9. Related to [**Figure 2**](#fig_DEG).


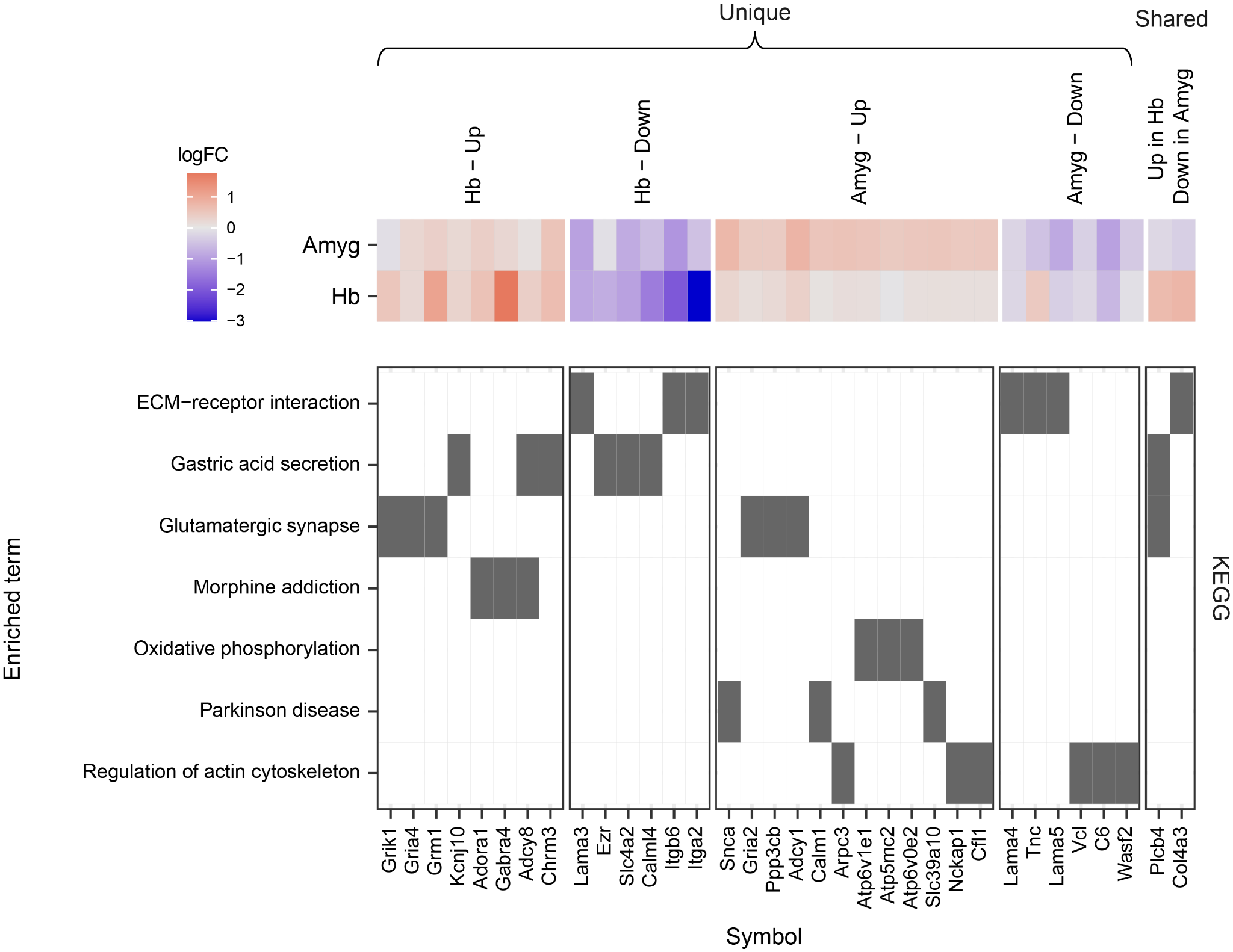


[**Figure S10**](#sfigu_GO_KEGG)**:** **Biological KEGG pathways dysregulated by chronic fentanyl self-administration in Hb and Amyg.** Tile plot displays DEG (x-axis) membership to an enriched pathway as a filled tile. Key DEGs from each pathway are shown, categorized by their unique or shared up- and down-regulation in Hb and Amyg. Top heatmap shows DEG mean-centered log_2_FC in Hb and Amyg. Related to [**Figure 2**](#fig_DEG), [**Table S8**](#sta_GO_KEGG_results_hab), [**Table S9**](#sta_GO_KEGG_results_amy).


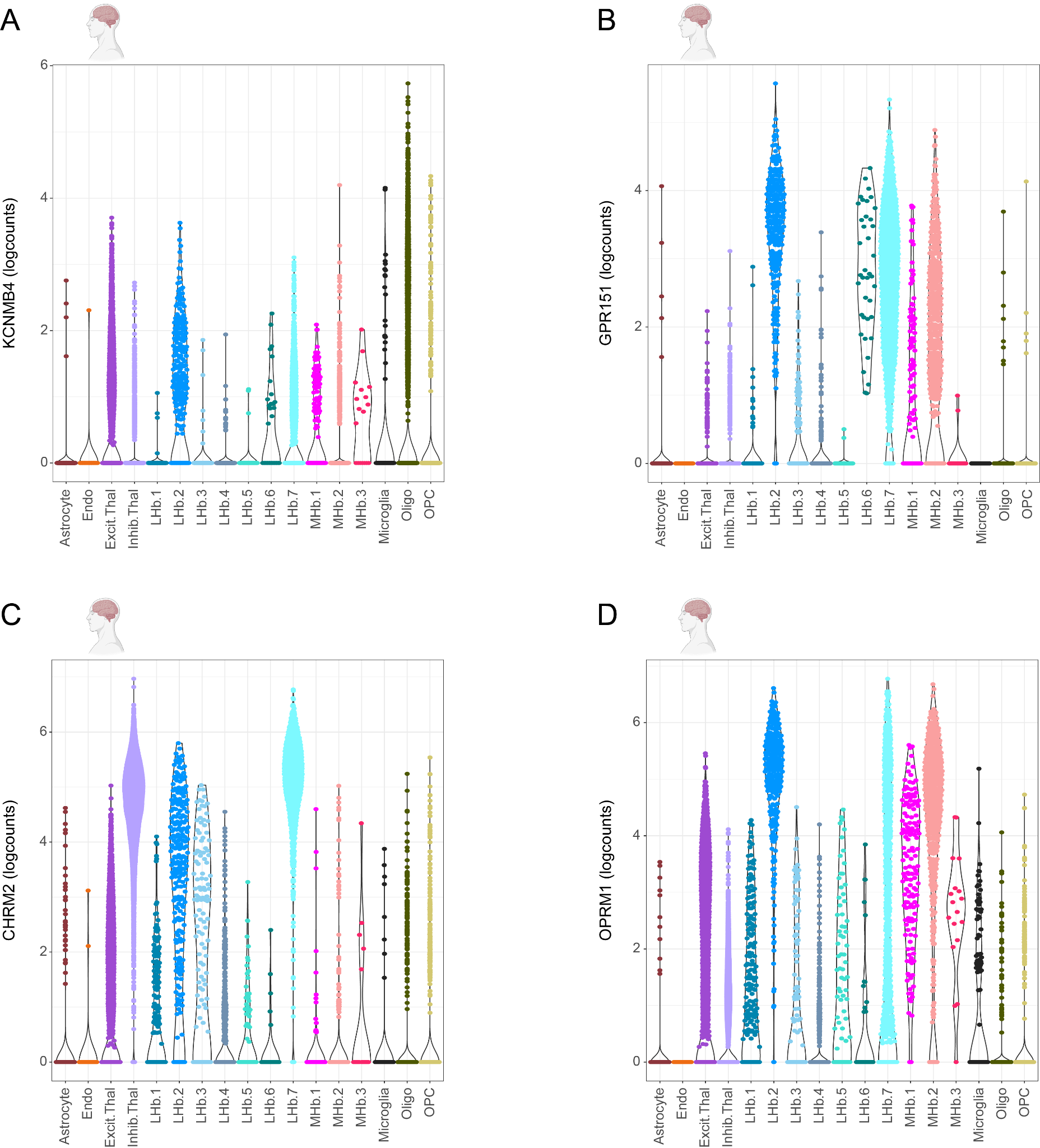


[**Figure S11**](#sfigu_habenulaPilot_iSEE)**:** **Expression of *KCNMB4*, *GPR151, CHRM2* and *OPRM1* in human habenula cell types. (A-D)** Violin plots showing expression of (**A**) *KCNMB4*, (**B**) *GPR151*, (**C**) *CHRM2*, and (**D**) *OPRM1* in human Hb cell types from Yalcinbas et al., 2025 [(50)](https://sciwheel.com/work/citation?ids=18372257&pre=&suf=&sa=0). *Kcnmb4*, *Gpr151*, and *Chrm2* mark the mouse LHb.6 subpopulation identified by Hashikawa et. al. [(49)](https://sciwheel.com/work/citation?ids=8601460&pre=&suf=&sa=0), which we found to be enriched in our rat upregulated Hb fentanyl DEGs. These genes are highly expressed in human LHb.2 and LHb.7 subpopulations, which also express *OPRM1*. This suggests that fentanyl-sensitive mouse LHb.6 may be conserved with these *OPRM1*-expressing human LHb.2 and LHb.7 neuronal populations. Related to [**Figure 3**](#fig_mouse_human_markers).

# Supplementary Tables

[**Table S1**](#stabl_SA_sessions_behavioral_data_raw)**: Behavioral raw data per LgA self-administration session per rat.** Session-by-session raw behavioral data pertaining to operant (lever press) behaviors and infusions from each rat across all long-access self-administration sessions. See [**Table S3**](#sta_sample_variables_dictionary) for the description of these variables.

[**Table S2**](#stabl_Rat_behavioral_covariates)**: Behavioral rat data in LgA sessions.** Individual rat data for behavioral covariates relating to fentanyl intake and intake escalation across long-access self-administration sessions. See [**Table S3**](#sta_sample_variables_dictionary) for the description of these variables.

[**Table S3**](#stabl_sample_variables_dictionary)**: Dictionary of sample variables.** Description of sample/rat variables analyzed throughout the study. Related to [**Table S1**](#sta_SA_sessions_behavioral_data_raw), [**Table S2**](#sta_Rat_behavioral_covariates), and [**Table S4**](#sta_sample_metadata_and_QCmetrics).

[**Table S4**](#stabl_sample_metadata_and_QCmetrics)**: Sample metadata and QC metrics.** Sample level variables analyzed, including data regarding rat self-administration sessions and sample batches for RNA extraction, library preparation, and sequencing, as well as quality control metrics. See [**Table S3**](#sta_sample_variables_dictionary) for the description of these variables.

[**Table S5**](#stabl_de_genes_Substance_habenula)**: DEGs for substance in Hb.** Metadata, *limma* DE statistics, and Ensembl gene annotation for DEGs obtained for fentanyl vs. saline in Hb. See *limma* [(114)](https://sciwheel.com/work/citation?ids=148089&pre=&suf=&sa=0) documentation for these statistics definitions. Related to [**Figure 2**](#fig_DEG)**,** [**Table S8**](#sta_GO_KEGG_results_hab), [**Table S10**](#sta_DEAs_results_all_genes_hab_amyg).

[**Table S6**](#stabl_de_genes_Substance_amygdala)**: DEGs for substance in Amyg.** Metadata, *limma* DE statistics, and Ensembl gene annotation for DEGs obtained for fentanyl vs. saline in Amyg. See *limma* [(114)](https://sciwheel.com/work/citation?ids=148089&pre=&suf=&sa=0) documentation for these statistics definitions. Related to [**Figure 2**](#fig_DEG)**,** [**Table S9**](#sta_GO_KEGG_results_amy)**,** [**Table S10**](#sta_DEAs_results_all_genes_hab_amyg).

[**Table S7**](#stabl_de_genes_common_Substance_hab_amy)**. Common DEGs for substance in habenula and amygdala.** Metadata, region-specific *limma* DE statistics, and Ensembl gene annotation for overlapping DEGs for fentanyl vs. saline in habenula and amygdala. See *limma* [(114)](https://sciwheel.com/work/citation?ids=148089&pre=&suf=&sa=0) documentation for these statistics definitions. Related to [**Figure 2**](#fig_DEG)**,** [**Table S5**](#sta_de_genes_Substance_habenula)**,** [**Table S6**](#sta_de_genes_Substance_amygdala).

[**Table S8**](#stabl_GO_KEGG_results_hab)**.** **Functional enrichment results for substance DEGs in Hb.**

GO terms for biological processes (BP), molecular functions (MF), cellular components (CC), and KEGG pathways that are significantly enriched in up- and down-regulated DEGs for fentanyl vs. saline in Hb. Provided are the ID and description of each significant term/pathway, the number and fraction of up/down-regulated DEGs annotated to each term (Count and GeneRatio, respectively), as well as the list of such genes (geneID), the fraction of genes in universe annotated to each term (BgRatio), fold of enrichment, *p*-value, and FDR-corrected *p*-value. Related to [**Figure 2**](#fig_DEG), [**Figure S10**](#sfi_GO_KEGG), [**Table S5**](#sta_de_genes_Substance_habenula).

[**Table S9**](#stabl_GO_KEGG_results_amy)**.** **Functional enrichment results for substance DEGs in Amyg.** Same as [**Table S8**](#sta_GO_KEGG_results_hab) but for up- and down-regulated DEGs for fentanyl vs. saline in Amyg. Related to [**Figure 2**](#fig_DEG), [**Figure S10**](#sfi_GO_KEGG), [**Table S6**](#sta_de_genes_Substance_amygdala).

[**Table S10**](#stabl_DEAs_results_all_genes_hab_amyg)**: Results for all DGE analyses and genes in Hb and Amyg.** Gene-level metadata and *limma* DE statistics of each gene for substance and rat behavior DGE analyses (fentanyl vs. saline, first hour infusion slope, total intake, and last session intake) in Hb and Amyg. See *limma* [(114)](https://sciwheel.com/work/citation?ids=148089&pre=&suf=&sa=0) documentation for these statistics definitions. Related to [**Figure 2**](#fig_DEG). This table includes all the data from [**Table S5**](#sta_de_genes_Substance_habenula), [**Table S6**](#sta_de_genes_Substance_amygdala), and [**Table S7**](#sta_de_genes_common_Substance_hab_amy).

[**Table S11**](#stabl_MeanRatio_markers_top100_hab_mouse)**: Top 100 *MeanRatio* marker genes per cell type in mouse Hb.** For all cell subpopulations and Hb neuronal subpopulations in the Hb complex of control mice obtained in Hashikawa et al., 2020 [(49)](https://sciwheel.com/work/citation?ids=8601460&pre=&suf=&sa=0), the top 100 most specific marker genes for each based on the *MeanRatio* method, are reported. See *DeconvoBuddies* [(63)](https://sciwheel.com/work/citation?ids=17727289&pre=&suf=&sa=0) documentation for column description. Cell_type_resolution column corresponds to the resolution of the cell subpopulation for which the gene is a marker. Related to [**Figure 3**](#fig_mouse_human_markers).

[**Table S12**](#stabl_MeanRatio_markers_top50_hab_human)**: Top 50 *MeanRatio* marker genes per cell type in human Hb.** For broad and fine cell types in the human Hb-enriched epithalamus of neurotypical control donors obtained in Yalcinbas et al., 2025 [(50)](https://sciwheel.com/work/citation?ids=18372257&pre=&suf=&sa=0), the top 50 most specific marker genes for each based on the *MeanRatio* method, are reported. See *DeconvoBuddies* [(63)](https://sciwheel.com/work/citation?ids=17727289&pre=&suf=&sa=0) documentation for column description. Cell_type_resolution column corresponds to the resolution of the cell type for which the gene is a marker. Related to [**Figure 3**](#fig_mouse_human_markers).

[**Table S13**](#stabl_MeanRatio_markers_top100_amy_rat)**: Top 100 *MeanRatio* marker genes per cell type in rat Amyg.** For main cell types and inhibitory neuronal subtypes in the Amyg of control rats obtained in Zhou et al., 2023 [(52)](https://sciwheel.com/work/citation?ids=15523346&pre=&suf=&sa=0), the top 100 most specific marker genes for each based on the *MeanRatio* method, are reported. See *DeconvoBuddies* [(63)](https://sciwheel.com/work/citation?ids=17727289&pre=&suf=&sa=0) documentation for column description. Cell_type_resolution column corresponds to the resolution of the cell type for which the gene is a marker. Related to [**Figure 4**](#fig_MAGMA).

[**Table S14**](#stabl_MeanRatio_markers_top100_amy_human)**: Top 100 *MeanRatio* marker genes per cell type in human Amyg.** For broad and fine cell types in the human Amyg of neurotypical control donors obtained in Yu et al., 2023 [(51)](https://sciwheel.com/work/citation?ids=14404156&pre=&suf=&sa=0), the top 100 most specific marker genes for each based on the *MeanRatio* method, are reported. See *DeconvoBuddies* [(63)](https://sciwheel.com/work/citation?ids=17727289&pre=&suf=&sa=0) documentation for column description. Cell_type_resolution column corresponds to the resolution of the cell type for which the gene is a marker. Related to [**Figure 4**](#fig_MAGMA).

#

# Supplementary References

[105. Fragale JE, James MH, Aston-Jones G. Intermittent self-administration of fentanyl induces a multifaceted addiction state associated with persistent changes in the orexin system. Addict Biol. 2021 May;26(3):e12946.](https://sciwheel.com/work/bibliography/10476141)

[106. Di Tommaso P, Chatzou M, Floden EW, Barja PP, Palumbo E, Notredame C. Nextflow enables reproducible computational workflows. Nat Biotechnol. 2017 Apr 11;35(4):316–9.](https://sciwheel.com/work/bibliography/3552253)

[107. Kim D, Paggi JM, Park C, Bennett C, Salzberg SL. Graph-based genome alignment and genotyping with HISAT2 and HISAT-genotype. Nat Biotechnol. 2019 Aug 2;37(8):907–15.](https://sciwheel.com/work/bibliography/7266361)

[108. Howe K, Dwinell M, Shimoyama M, Corton C, Betteridge E, Dove A, et al. The genome sequence of the Norway rat, Rattus norvegicus Berkenhout 1769. Wellcome Open Res. 2021 May 18;6:118.](https://sciwheel.com/work/bibliography/11724063)

[109. Martin FJ, Amode MR, Aneja A, Austine-Orimoloye O, Azov AG, Barnes I, et al. Ensembl 2023. Nucleic Acids Res. 2023 Jan 6;51(D1):D933–41.](https://sciwheel.com/work/bibliography/13870406)

[110. Huber W, Carey VJ, Gentleman R, Anders S, Carlson M, Carvalho BS, et al. Orchestrating high-throughput genomic analysis with Bioconductor. Nat Methods. 2015 Feb;12(2):115–21.](https://sciwheel.com/work/bibliography/111791)

[111. Robinson MD, McCarthy DJ, Smyth GK. edgeR: a Bioconductor package for differential expression analysis of digital gene expression data. Bioinformatics. 2010 Jan 1;26(1):139–40.](https://sciwheel.com/work/bibliography/673952)

[112. McCarthy DJ, Campbell KR, Lun ATL, Wills QF. Scater: pre-processing, quality control, normalization and visualization of single-cell RNA-seq data in R. Bioinformatics. 2017 Apr 15;33(8):1179–86.](https://sciwheel.com/work/bibliography/3436659)

[113. Hoffman GE, Schadt EE. variancePartition: interpreting drivers of variation in complex gene expression studies. BMC Bioinformatics. 2016 Nov 25;17(1):483.](https://sciwheel.com/work/bibliography/3280148)

[114. Ritchie ME, Phipson B, Wu D, Hu Y, Law CW, Shi W, et al. limma powers differential expression analyses for RNA-sequencing and microarray studies. Nucleic Acids Res. 2015 Apr 20;43(7):e47.](https://sciwheel.com/work/bibliography/148089)

[115. Benjamini Y, Hochberg Y. Controlling the false discovery rate: a practical and powerful approach to multiple testing. Journal of the Royal Statistical Society: Series B (Methodological). 1995 Jan;57(1):289–300.](https://sciwheel.com/work/bibliography/6279401)

[116. Gene Ontology Consortium. Gene Ontology Consortium: going forward. Nucleic Acids Res. 2015 Jan;43(Database issue):D1049-56.](https://sciwheel.com/work/bibliography/963677)

[117. Kanehisa M, Goto S. KEGG: Kyoto encyclopedia of genes and genomes. Nucleic Acids Res. 2000 Jan 1;28(1):27–30.](https://sciwheel.com/work/bibliography/718914)

[118. Yu G, Wang L-G, Han Y, He Q-Y. clusterProfiler: an R package for comparing biological themes among gene clusters. OMICS. 2012 May;16(5):284–7.](https://sciwheel.com/work/bibliography/1509330)

[119. Dyer SC, Austine-Orimoloye O, Azov AG, Barba M, Barnes I, Barrera-Enriquez VP, et al. Ensembl 2025. Nucleic Acids Res. 2025 Jan 6;53(D1):D948–57.](https://sciwheel.com/work/bibliography/17292735)

[120. Team RC. R: A Language and Environment for Statistical Computing. Vienna, Austria: CRAN; 2025.](https://sciwheel.com/work/bibliography/15967786)

[121. Wickham H. ggplot2: Elegant Graphics for Data Analysis (Use R!). 2nd ed. Cham: Springer; 2016.](https://sciwheel.com/work/bibliography/12688379)
