# Supplementary figures and images for: Transcriptional Response to Chronic Long‐Access Fentanyl Self‐Administration in Rat Habenula and Amygdala
Source: Addict Biol. 2026 Jul 14;31(7):e70179. doi: 10.1111/adb.70179 (PMC13366401; doi:10.1111/adb.70179)

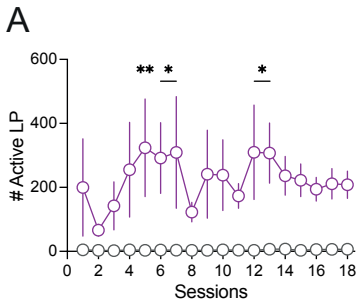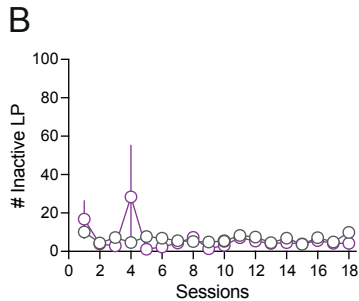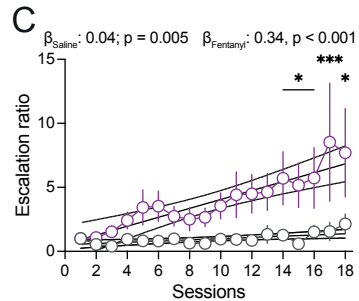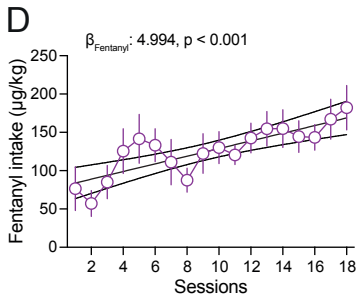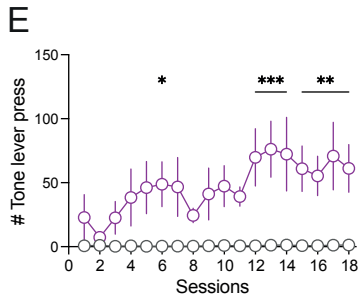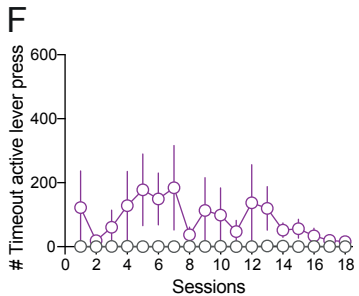

○ Saline ○ Fentanyl

Supplement: Supplementary file 2 — Figure S1: Additional behaviour metrics from LgA sessions. Mean number of total (A) active (mixed‐effects model [REML], substance effect: F 1,17 = 10.97, p = 0.0041; session effect: F 17,273 = 1.44, p = 0.118; substance × session interaction: F 17,273 = 1.4, p = 0.136; Šídák post hoc comparisons) and (B) inactive lever presses per LgA session for saline and fentanyl rats (mixed‐effects model [REML], substance effect: F 1,17 = 0.010, p = 0.918; session effect: F 17,273 = 1.26, p = 0.214; substance × session interaction: F 17,273 = 1.19, p = 0.265). (C) Escalation ratio, an alternative metric to quantify infusion escalation across sessions. This ratio is calculated by normalizing each rat's LgA infusion counts relative to their infusion count on the first LgA session (mixed‐effects model [REML], substance effect: F 1,17 = 8.77, p = 0.0088; session effect: F 17,273 = 4.69, p < 0.001; substance × session interaction: F 17,273 = 2.81, p < 0.001; Šídák post hoc comparisons). (D) Fentanyl intake (μg/kg) per LgA session. (E) Number of active lever presses performed during the 2.8 s infusion and tone presentation period (mixed‐effects model [REML], substance effect: F 1,17 = 19.4, p < 0.001; session effect: F 17,273 = 2.91, p < 0.001; substance × session interaction: F 17,273 = 2.82, p < 0.001; Šídák post hoc comparisons). (F) Number of active lever presses performed during the 20‐s timeout period (mixed‐effects model [REML], substance effect: F 1,17 = 3.06, p = 0.098; session effect: F 17,273 = 1.41, p = 0.131; substance × session interaction: F 17,273 = 1.41, p = 0.129). Data shown as mean across rats ± SEM. Black lines represent linear regression with 95% confidence intervals. Saline: n = 11 rats; fentanyl: n = 8 rats. * denotes p < 0.05; ** denotes p < 0.01; *** denotes p < 0.001. [file ADB-31-e70179-s010.pdf]

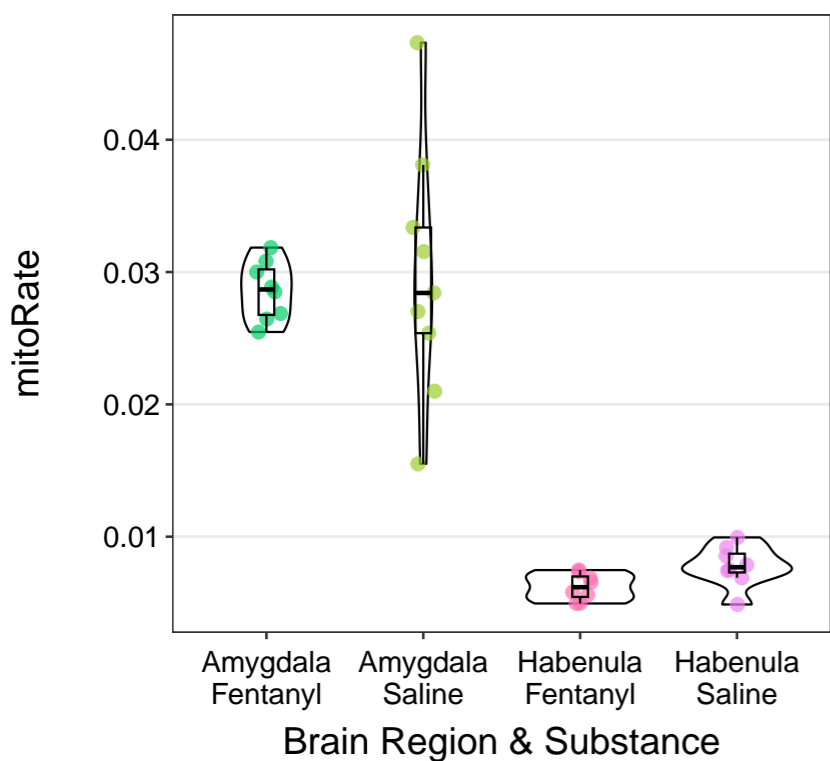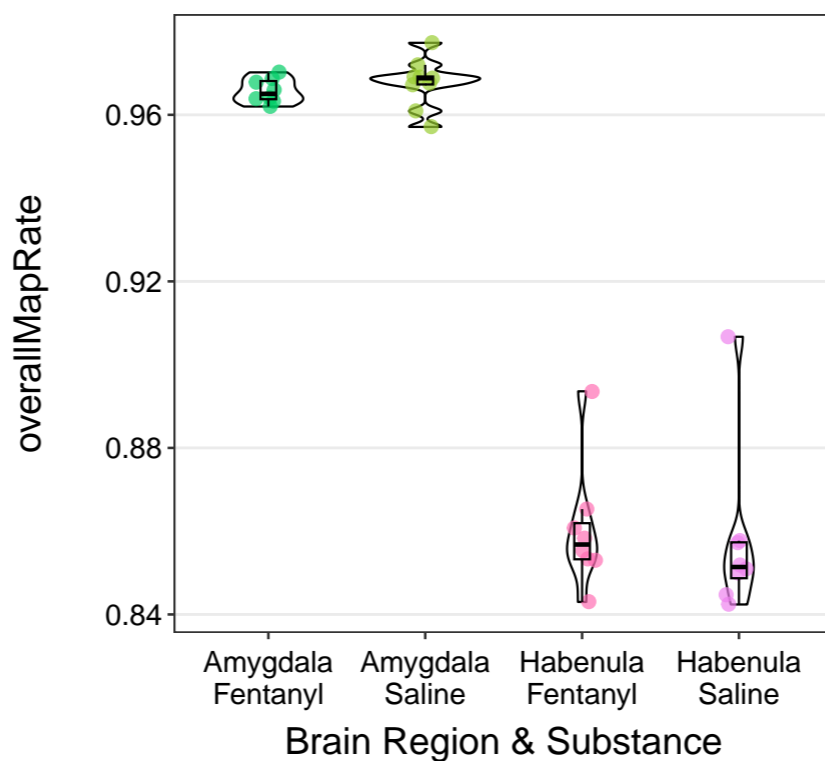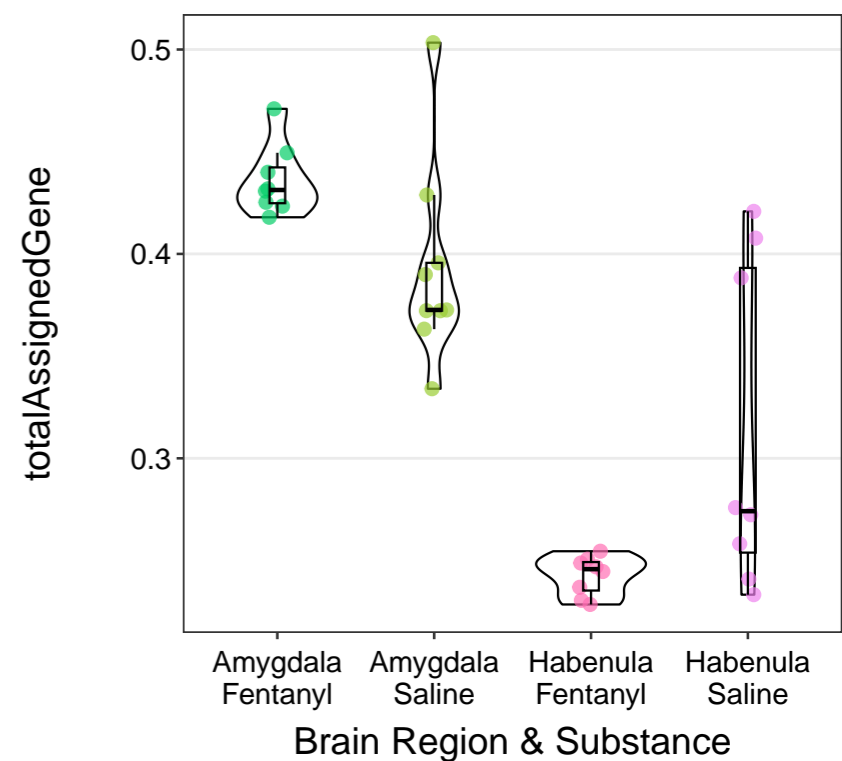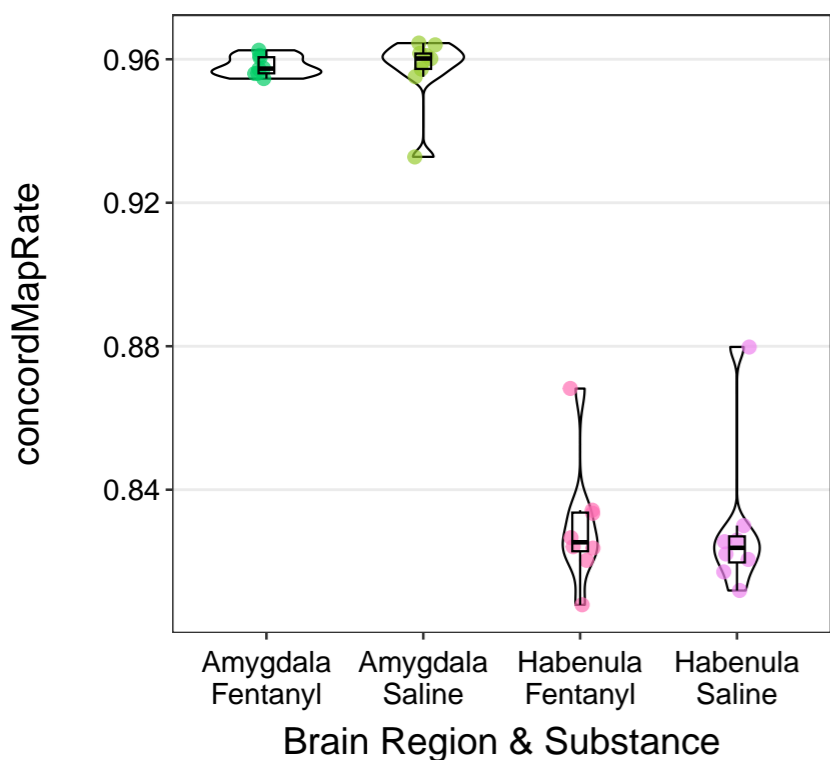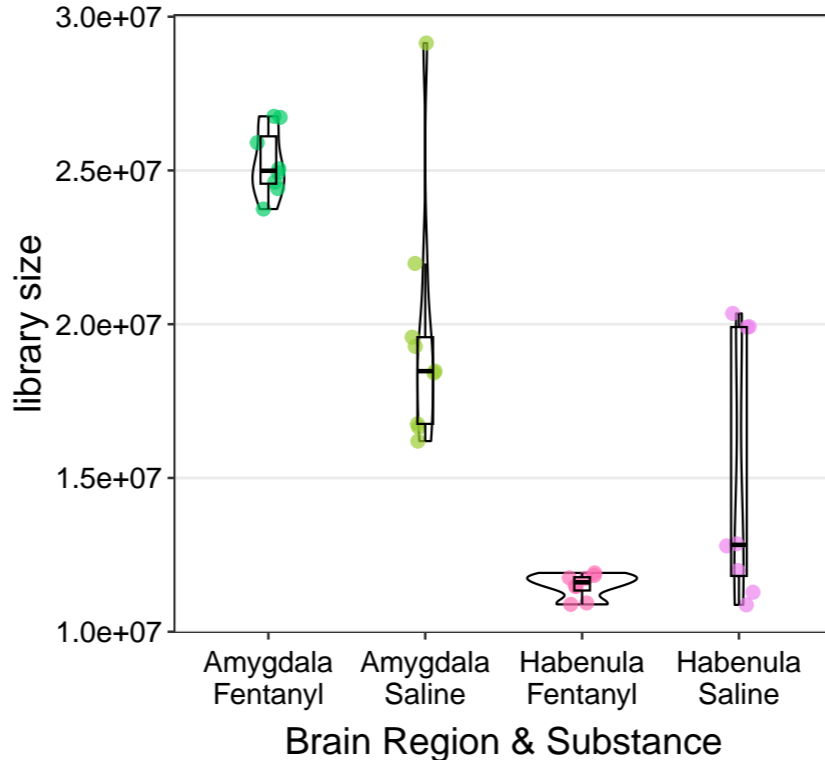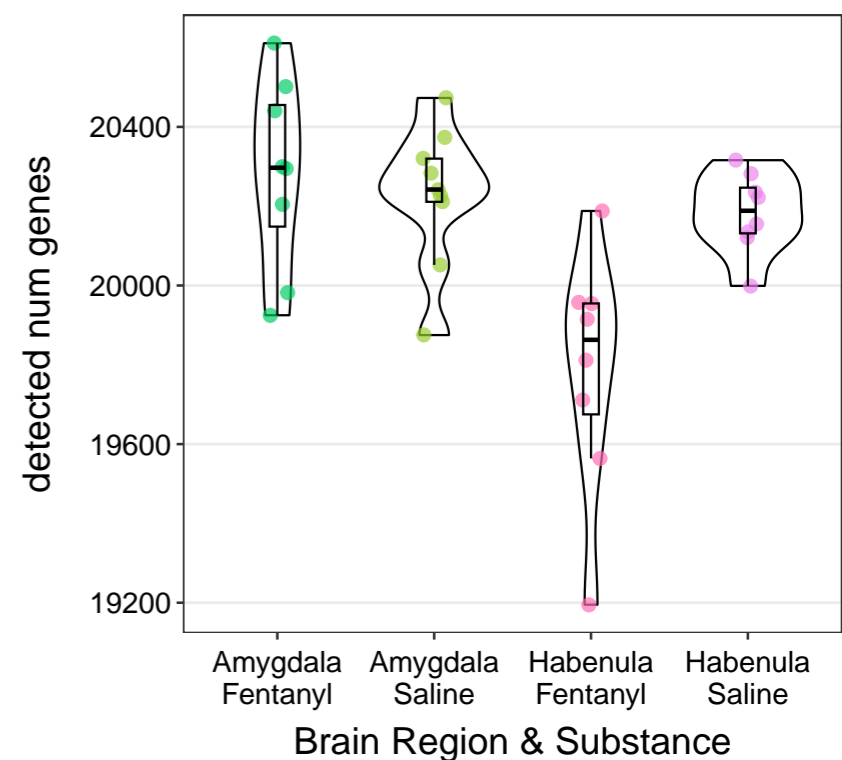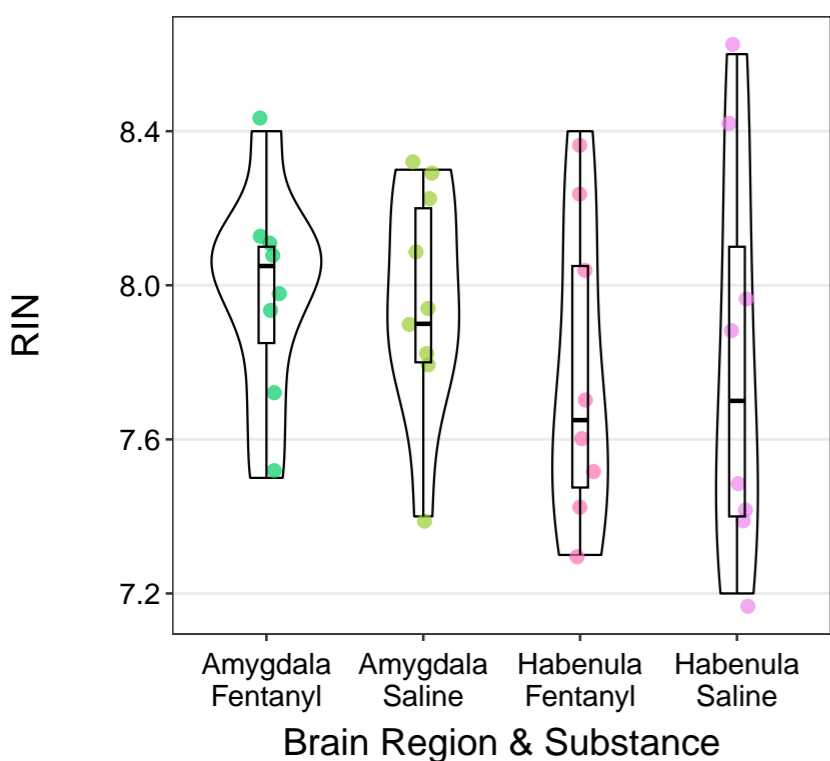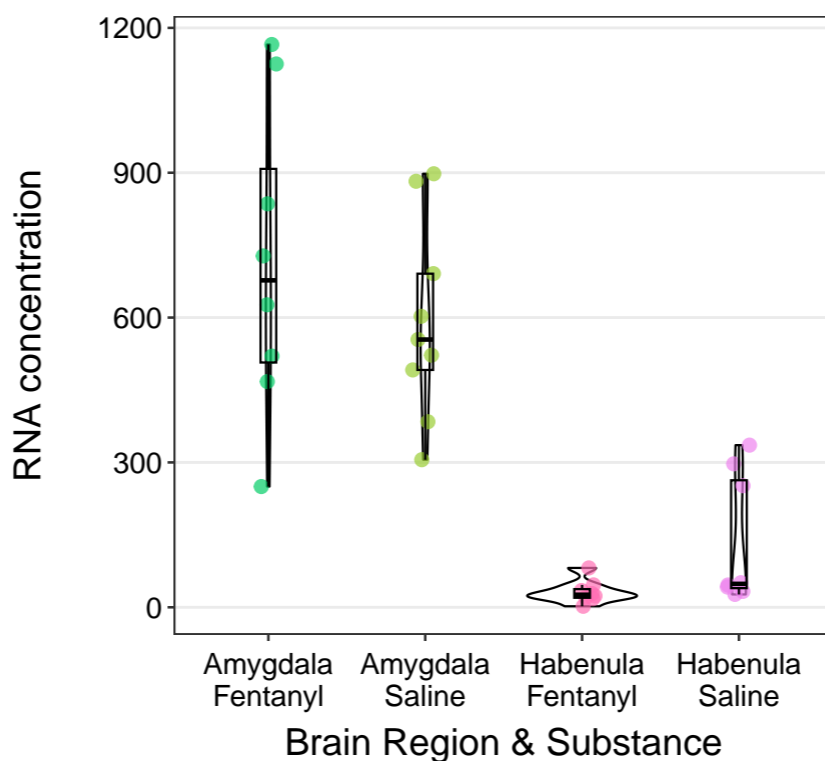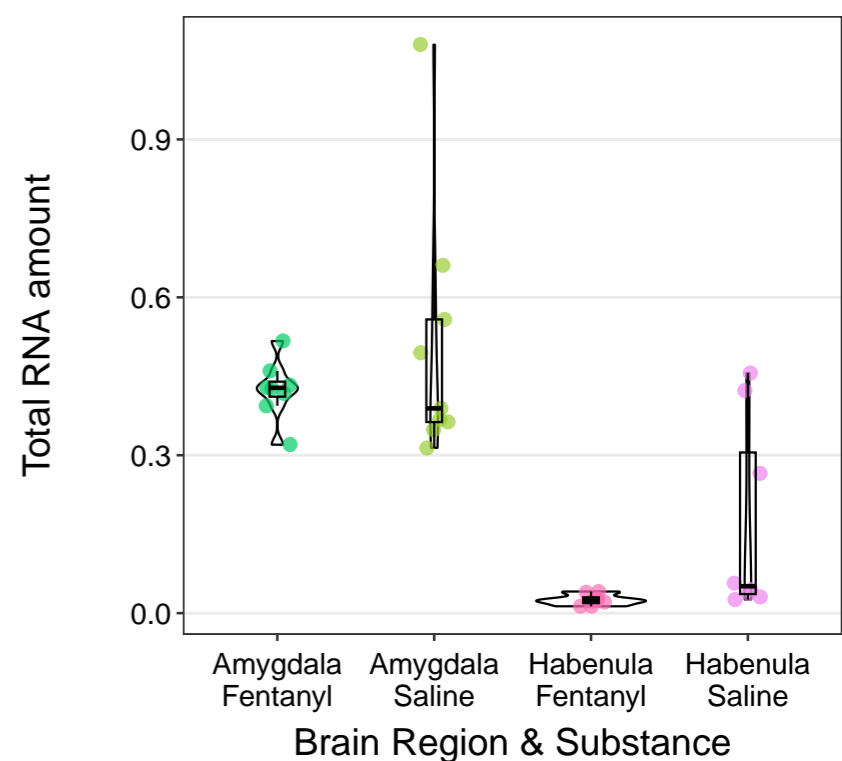

Supplement: Supplementary file 3 — Figure S2: Quality control metrics for Hb and Amyg samples. Comparison of the QC metrics examined in this study for habenula and amygdala fentanyl and saline samples. Note that different Illumina library preparation kits were used for each brain region, thus confounding brain region and kit differences, which motivated independent analyses for each brain region. See Table S3 for the description of these QC metrics. [file ADB-31-e70179-s021.pdf]

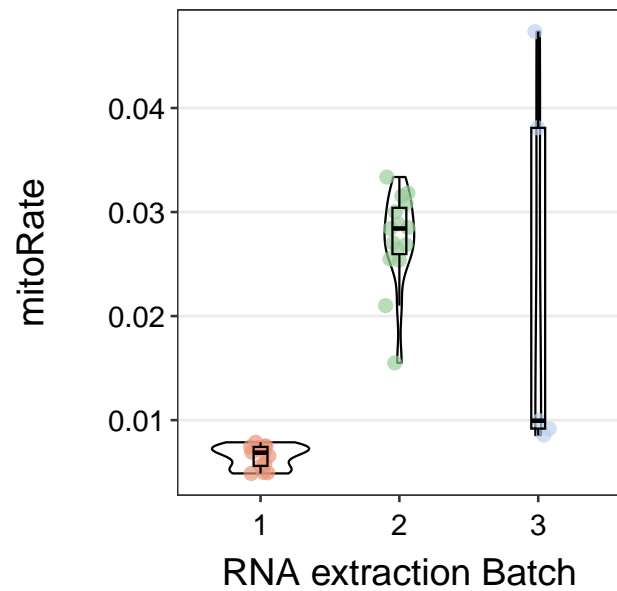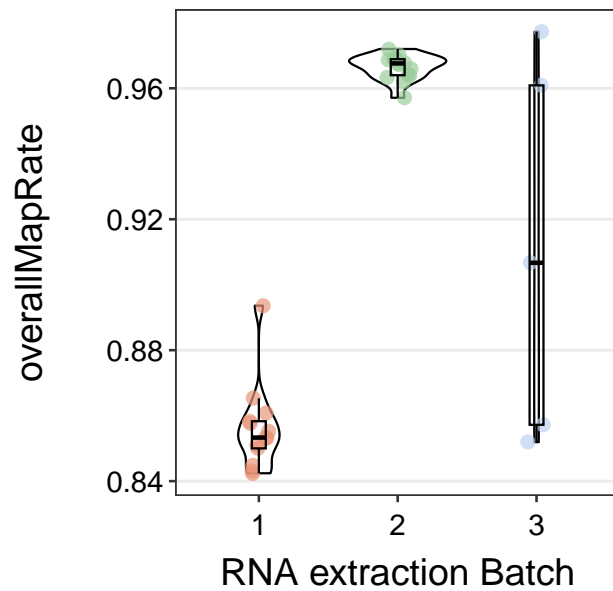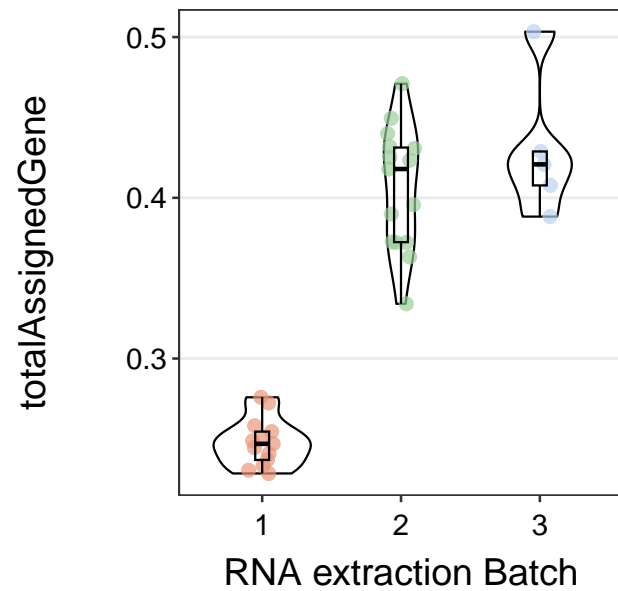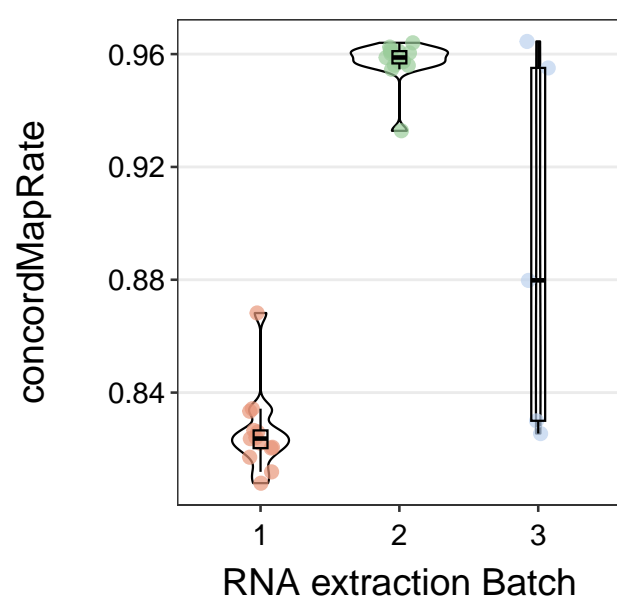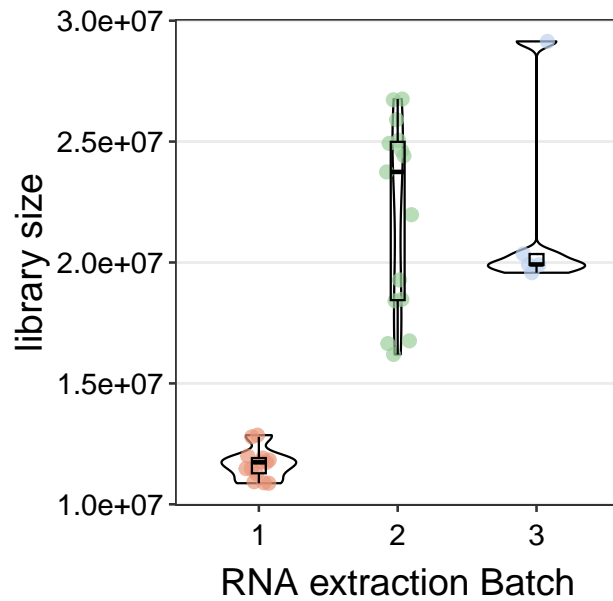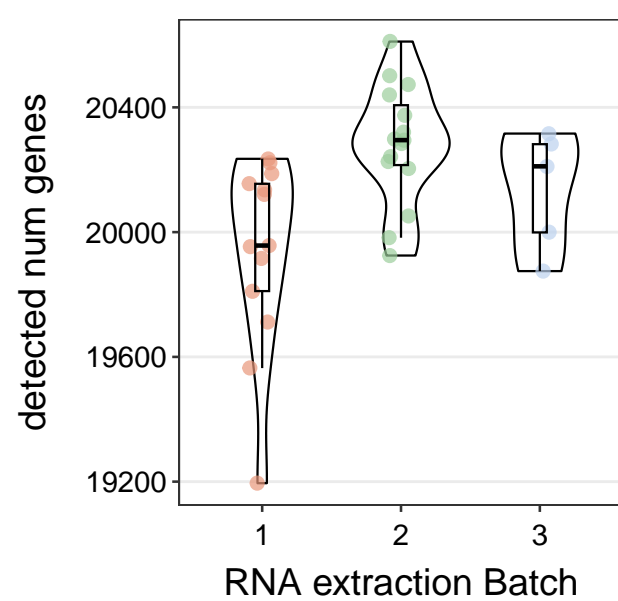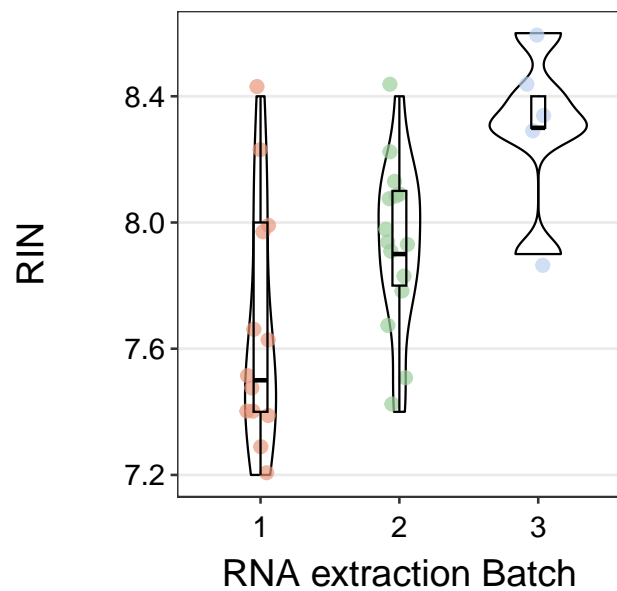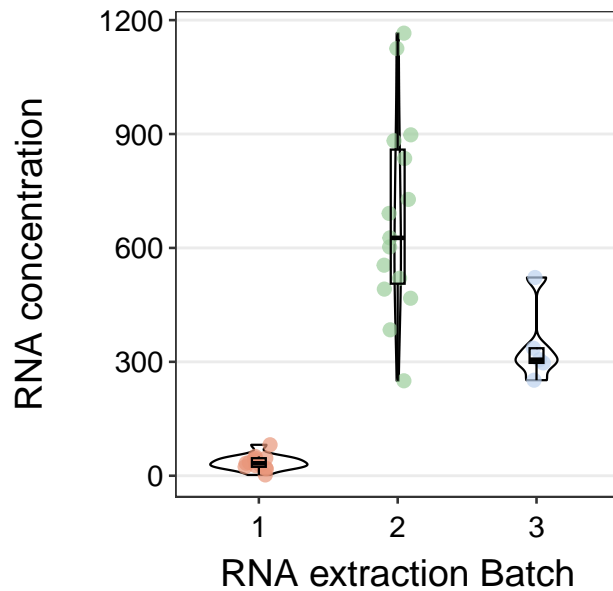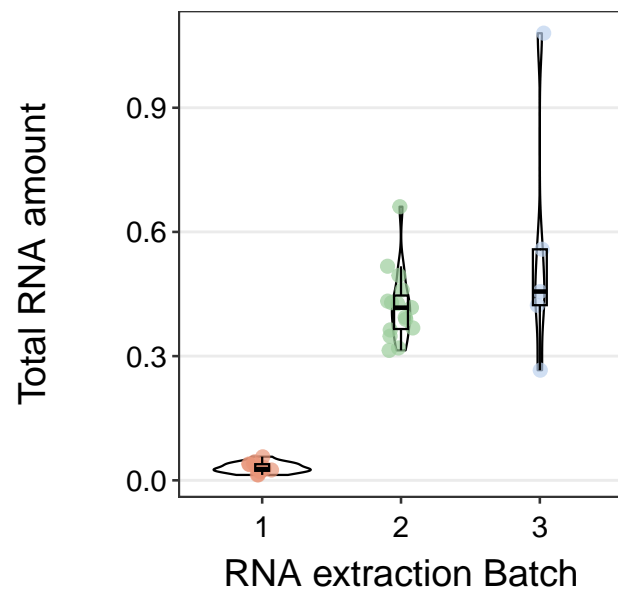

Supplement: Supplementary file 4 — Figure S3: Quality control metrics for samples across RNA extraction batches. Comparison of QC metrics of samples from the first (only Hb samples), second (only Amyg samples) and third batch for RNA extraction (additional Hb and Amyg samples). See Table S3 for the description of these QC metrics. [file ADB-31-e70179-s026.pdf]

A

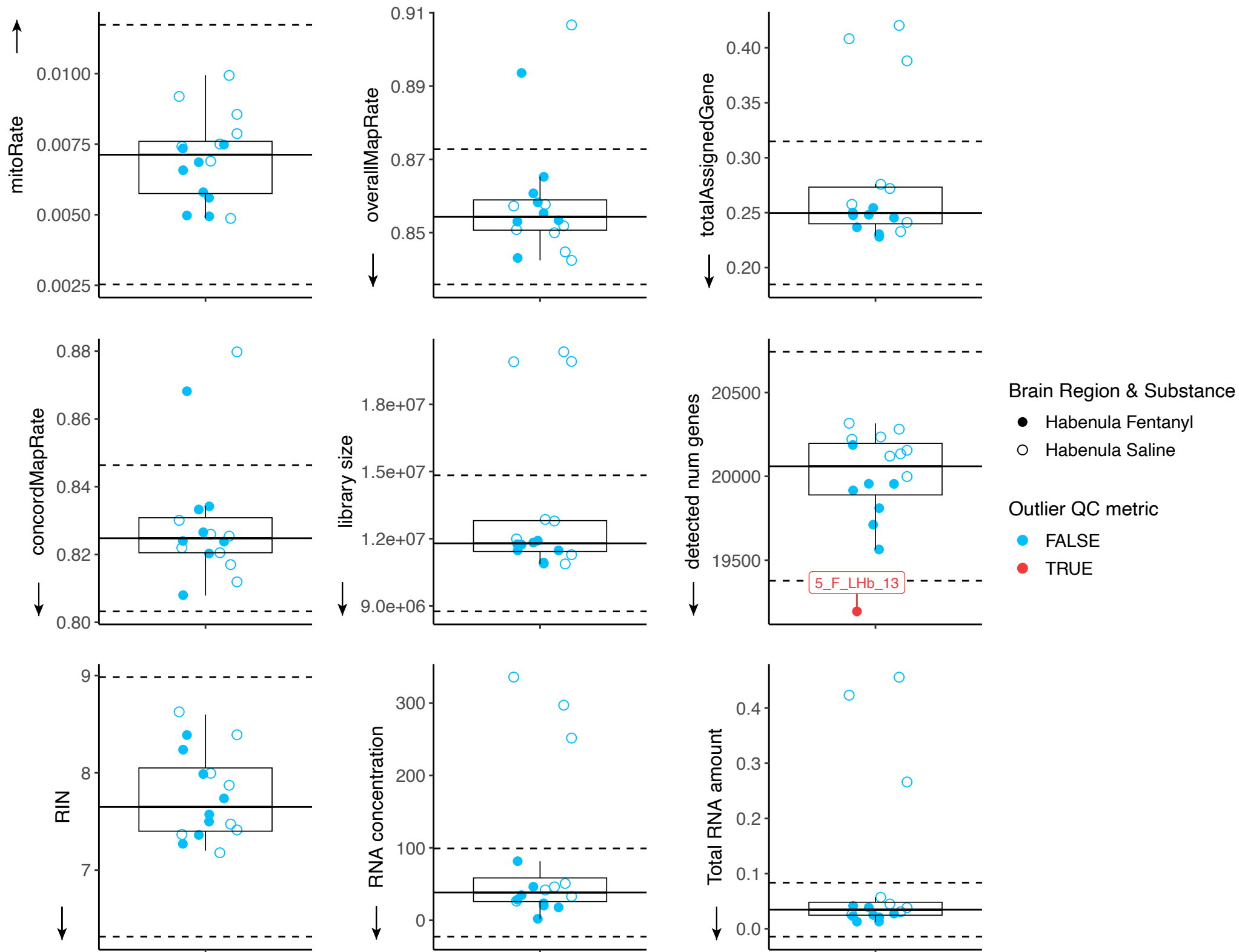

B

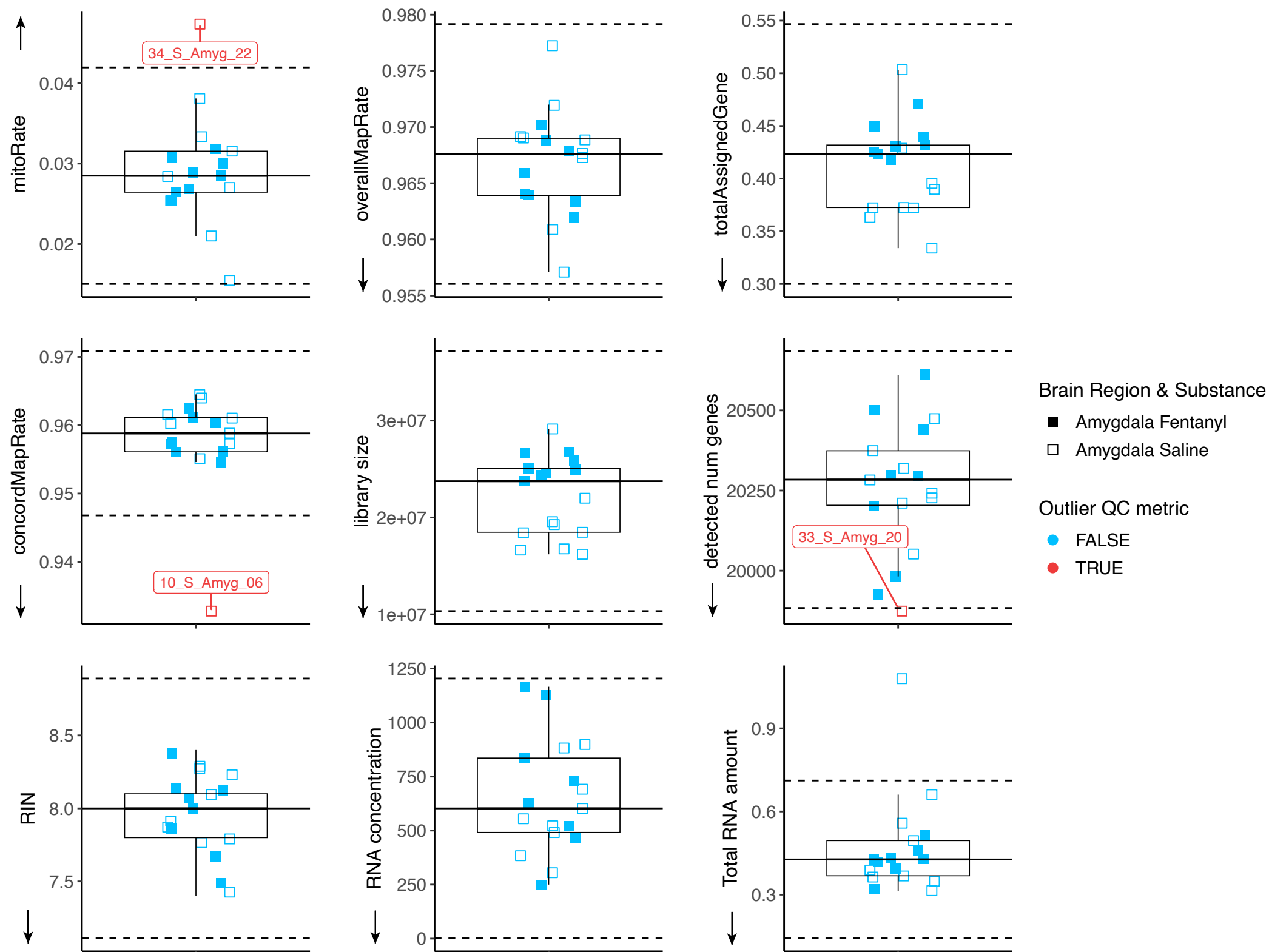

Supplement: Supplementary file 6 — Figure S5: Low‐quality sample identification. Detection of low‐quality metrics for (A) Hb and (B) Amyg fentanyl (filled circles/squares) and saline (empty circles/squares) samples. QC metric outliers (in red) were identified as those being 3 median‐absolute‐deviations (MAD; dotted lines) away from the median (solid line). Only lower outliers were considered poor‐quality for all QC metrics except mitoRate, for which higher outliers were considered instead (indicated by arrows). Samples with outlier QC metrics are labelled and were subjected to further evaluation in downstream analyses (Figure S7). See Table S3 for the description of these QC metrics. [file ADB-31-e70179-s019.pdf]

**A**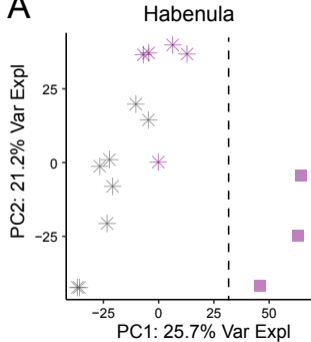**B**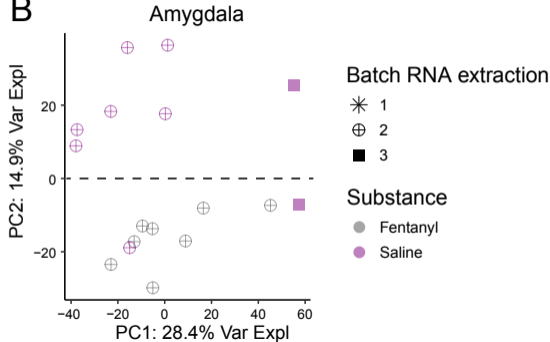

Supplement: Supplementary file 7 — Figure S6: Principal component analysis. PC1 versus PC2 for gene expression in (A) Hb and (B) Amyg samples. Percentages of variance explained by each PC are indicated on the axes. Samples are shaped by RNA extraction batch and coloured by substance. [file ADB-31-e70179-s006.pdf]

A

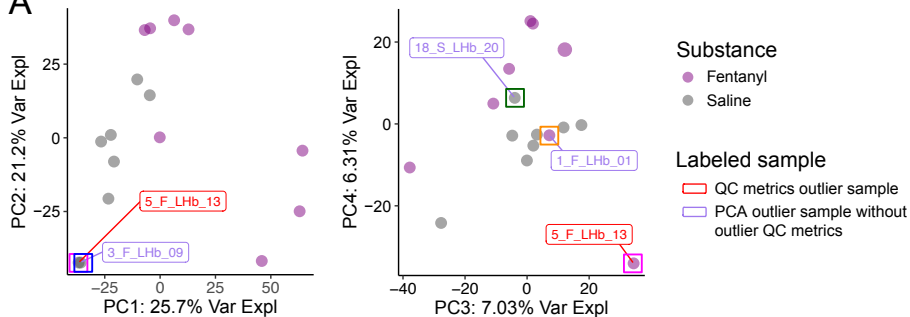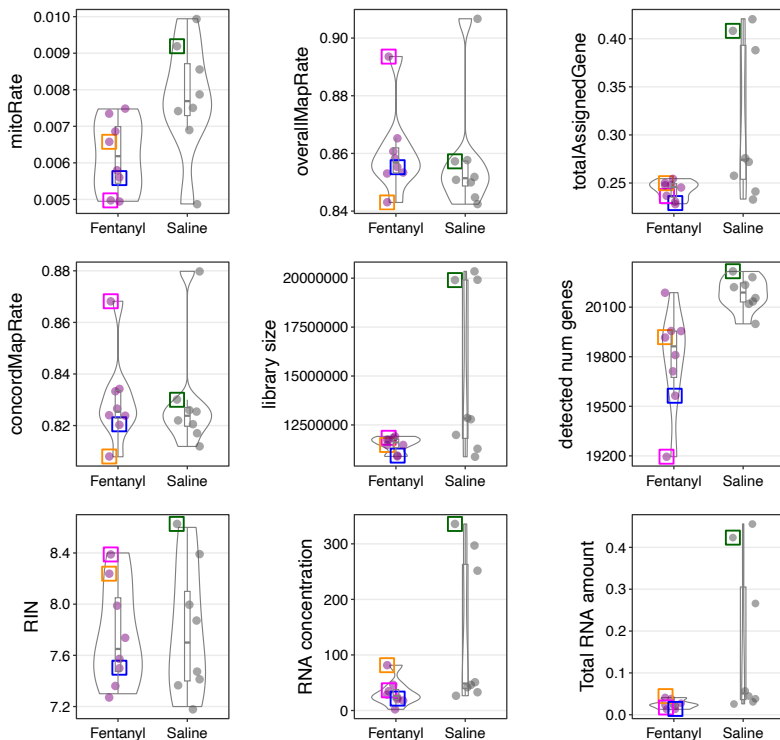

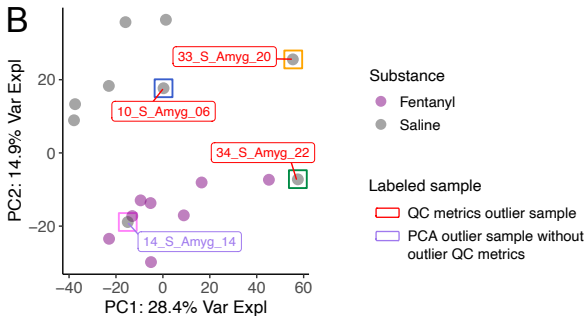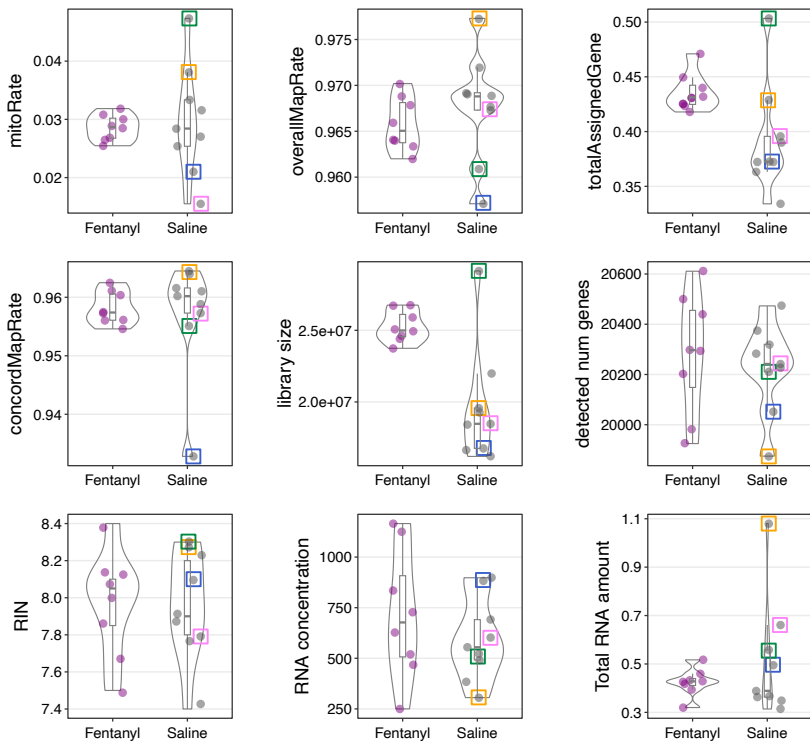

Supplement: Supplementary file 8 — Figure S7: Manual sample quality examination based on PCA. PCx versus PCy (top) for (A) Hb and (B) Amyg samples. QC metrics outlier samples are labelled in red (see Figure S5); samples segregated from the rest in each PC plot, as well as fentanyl and saline samples closer to samples from the other substance group were considered PCA outlier samples and are labelled in purple. The percentage of variance explained by each PC is shown on axis labels. For both, QC metrics and PCA outlier samples, all their QC metrics were re‐examined (bottom box plots); different coloured squares indicate the different outlier samples. Samples in all plots are coloured by substance. See Table S3 for the description of these QC metrics. [file ADB-31-e70179-s001.pdf]

A

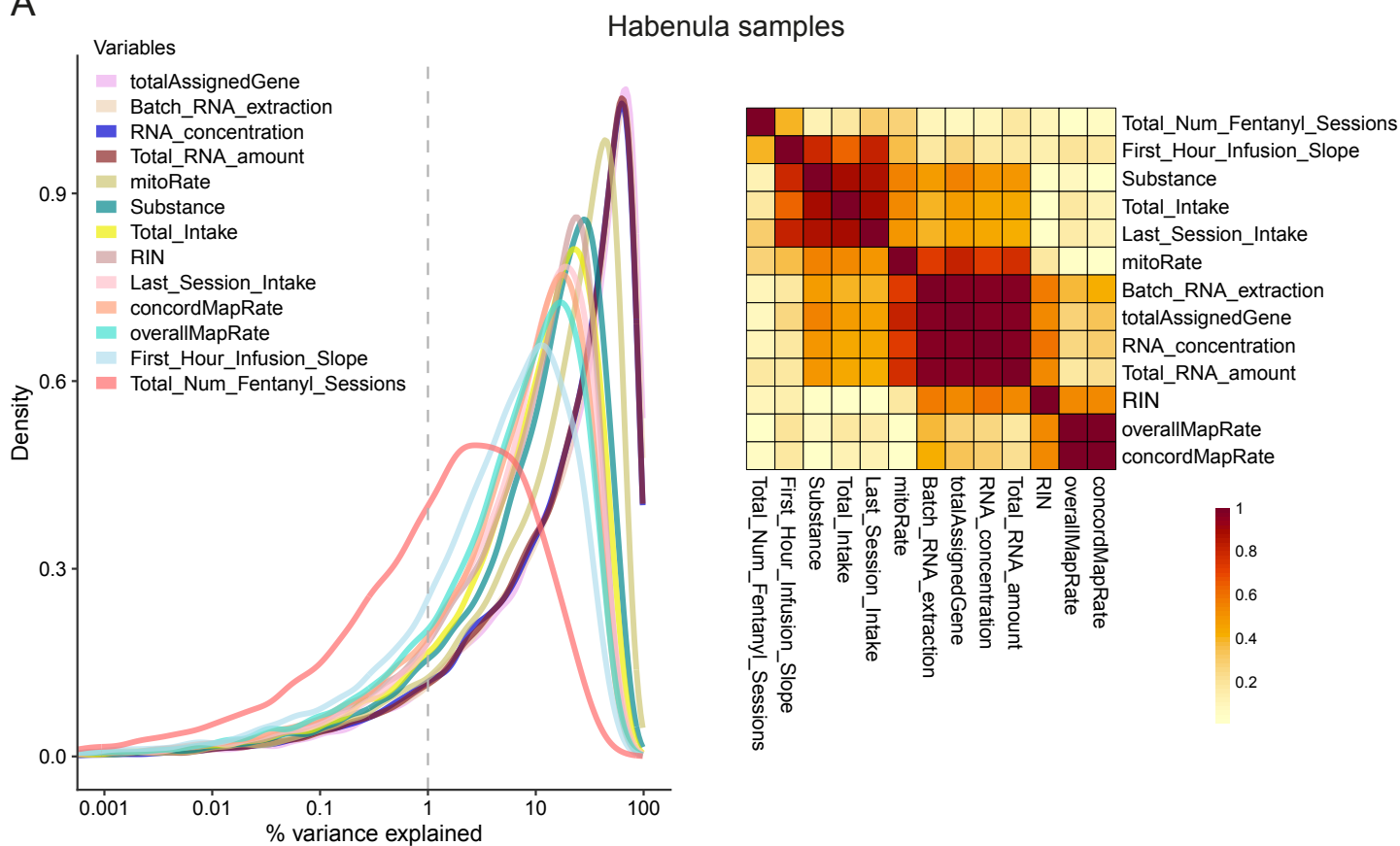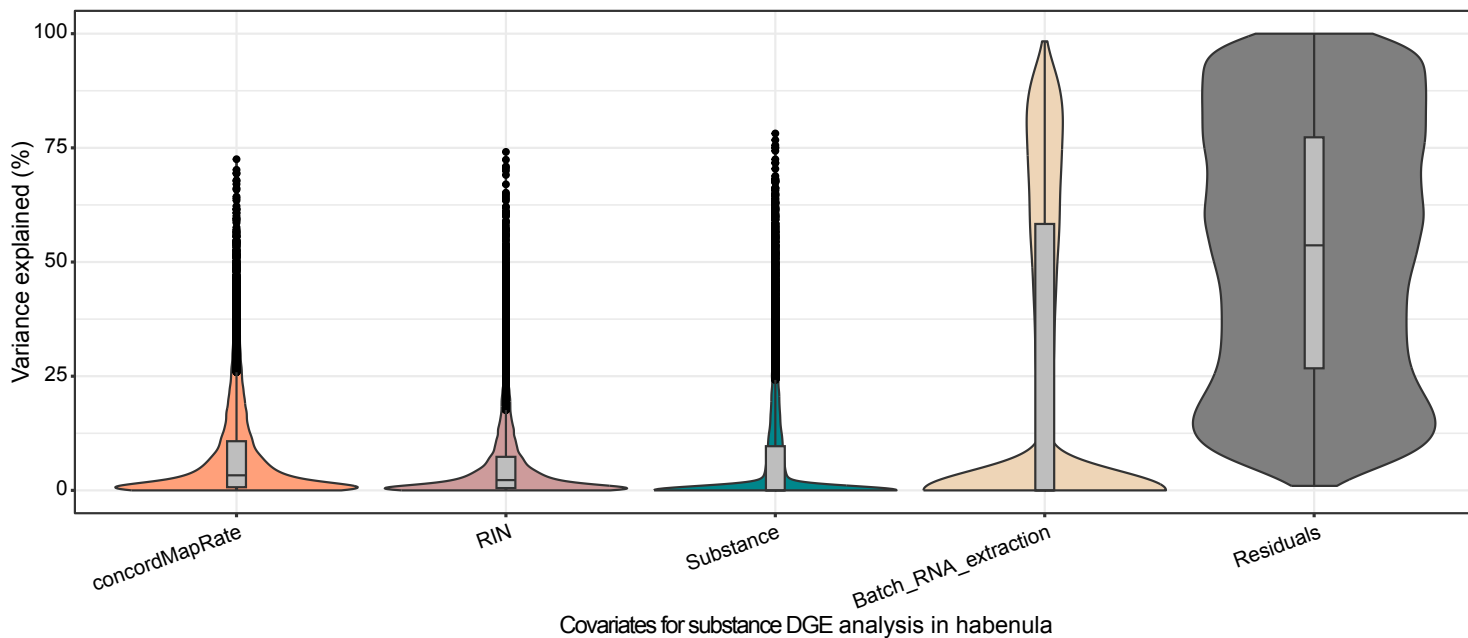

B

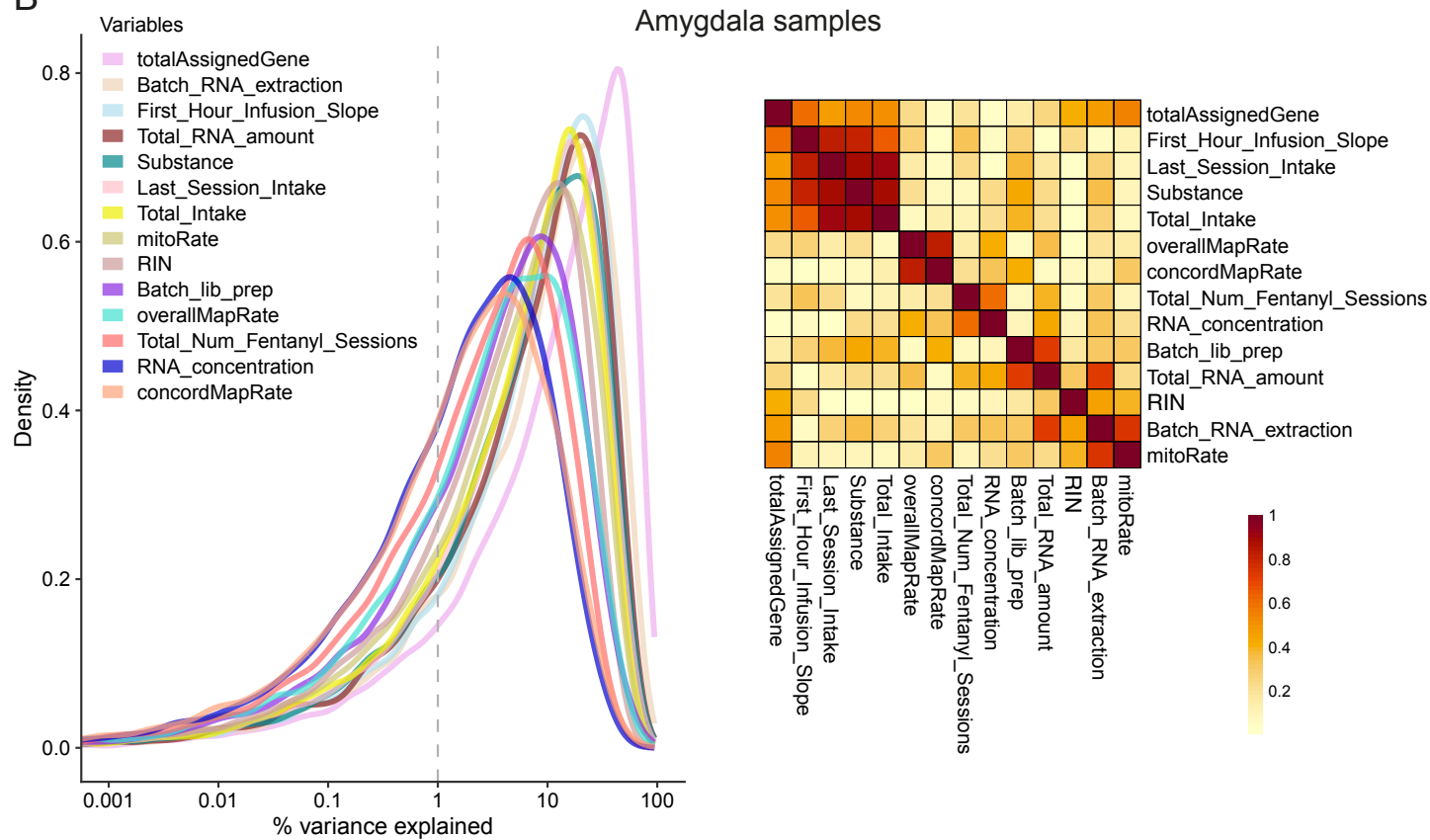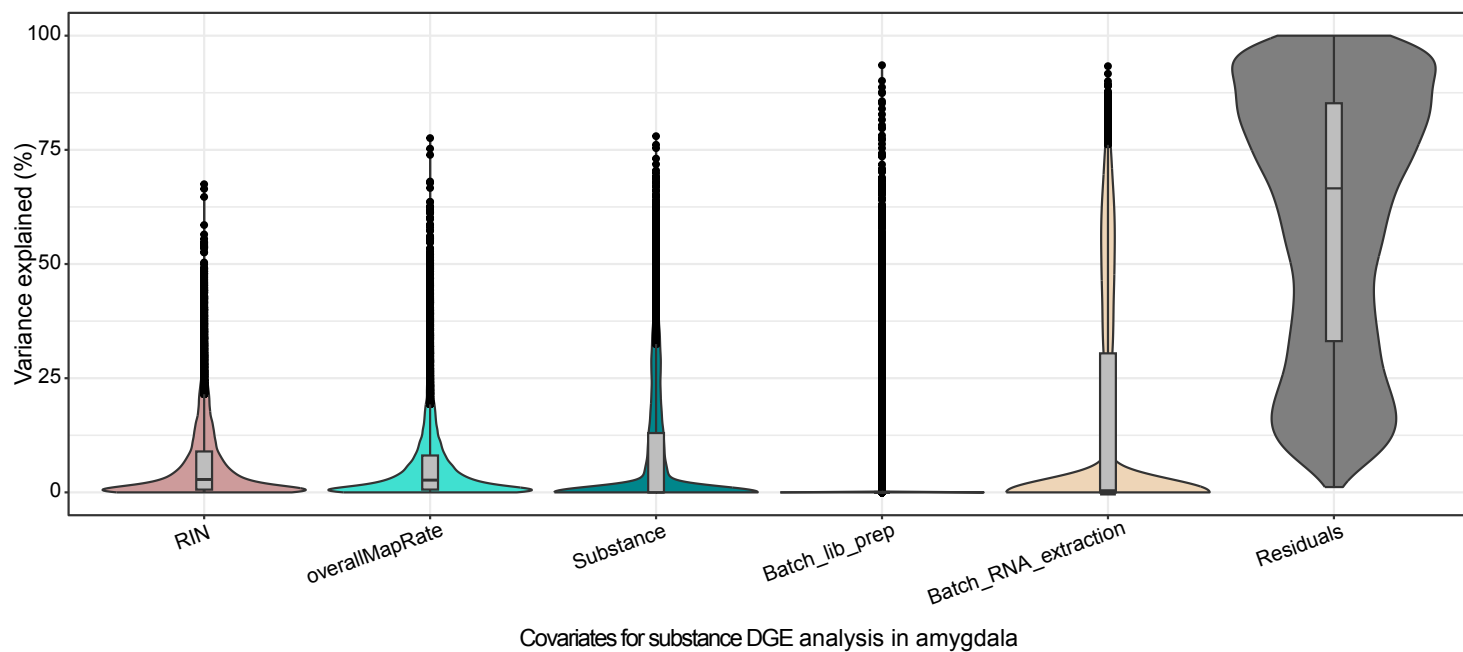

C

## Habenula fentanyl samples

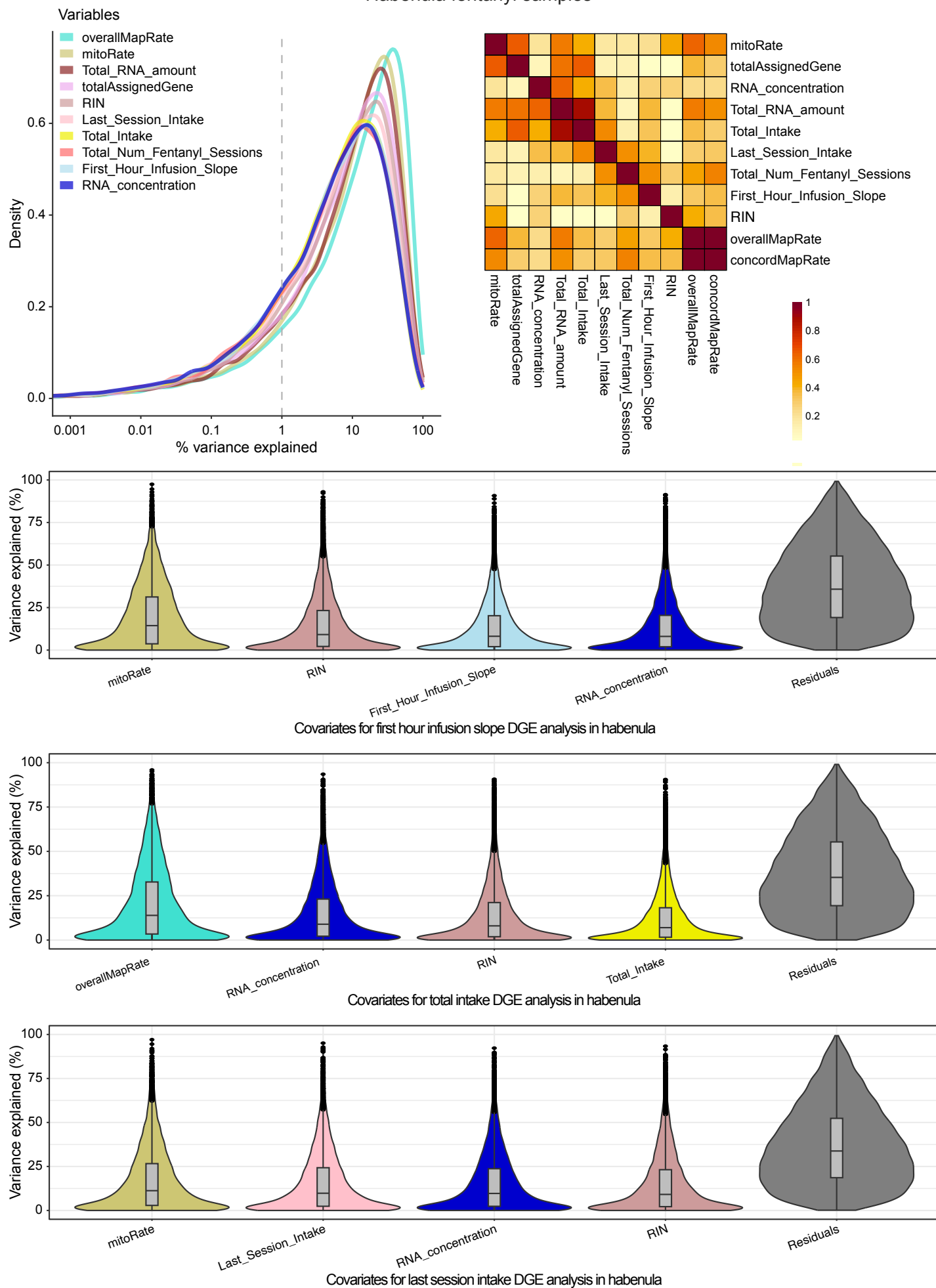

D

## Amygdala fentanyl samples

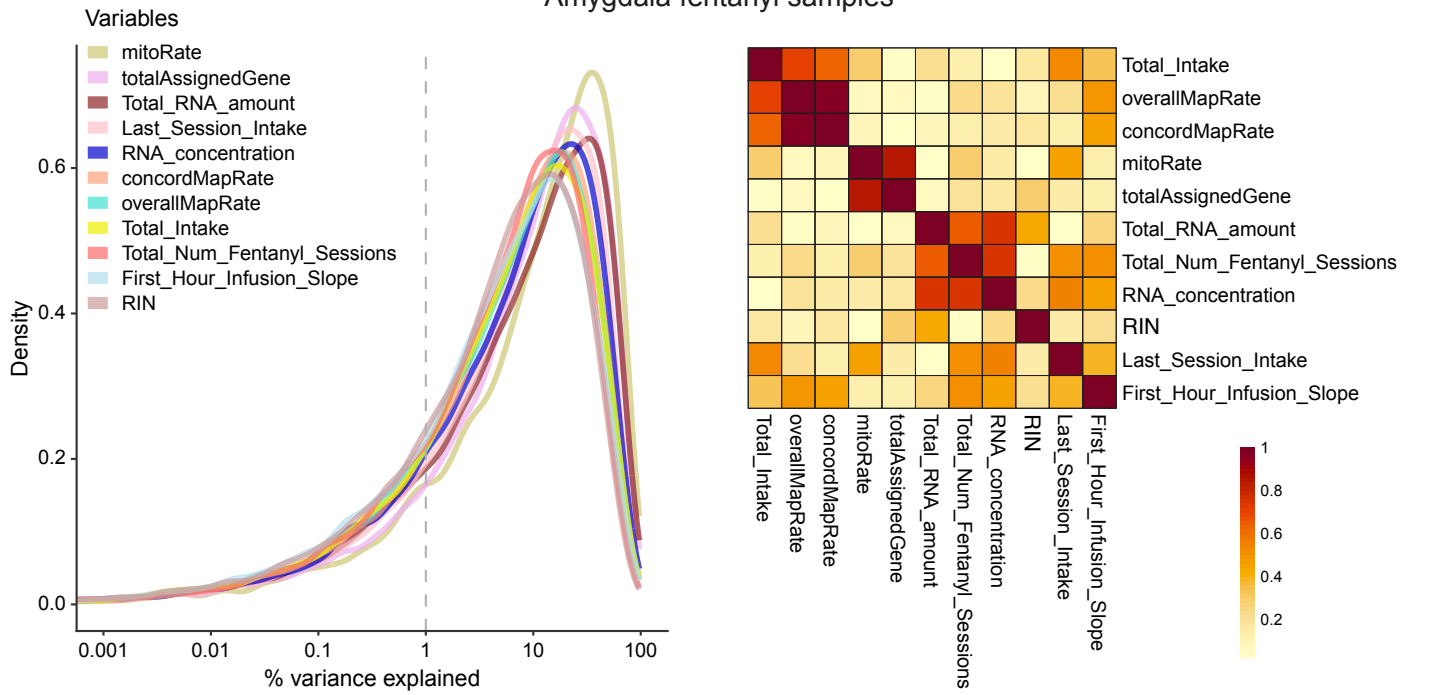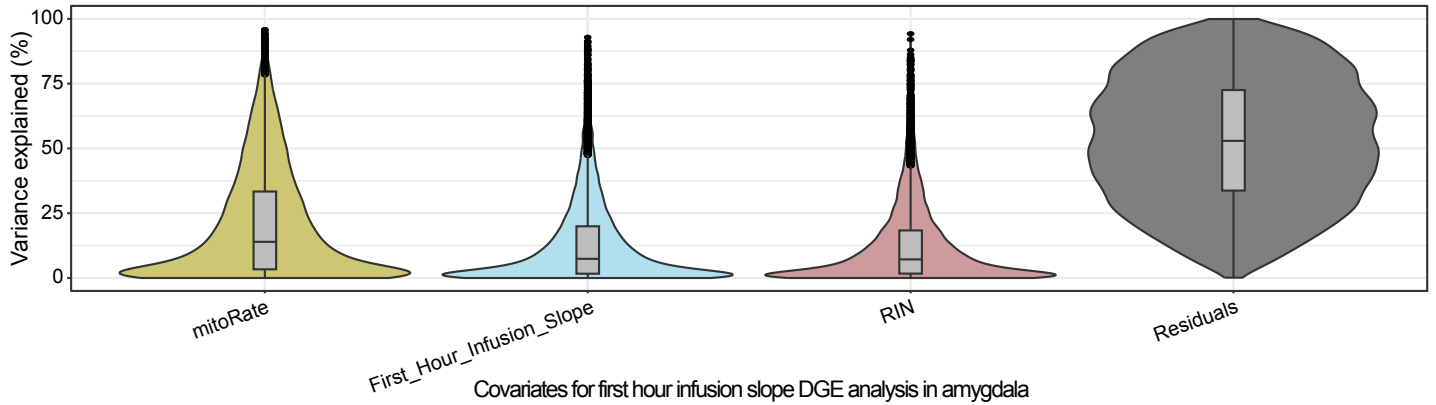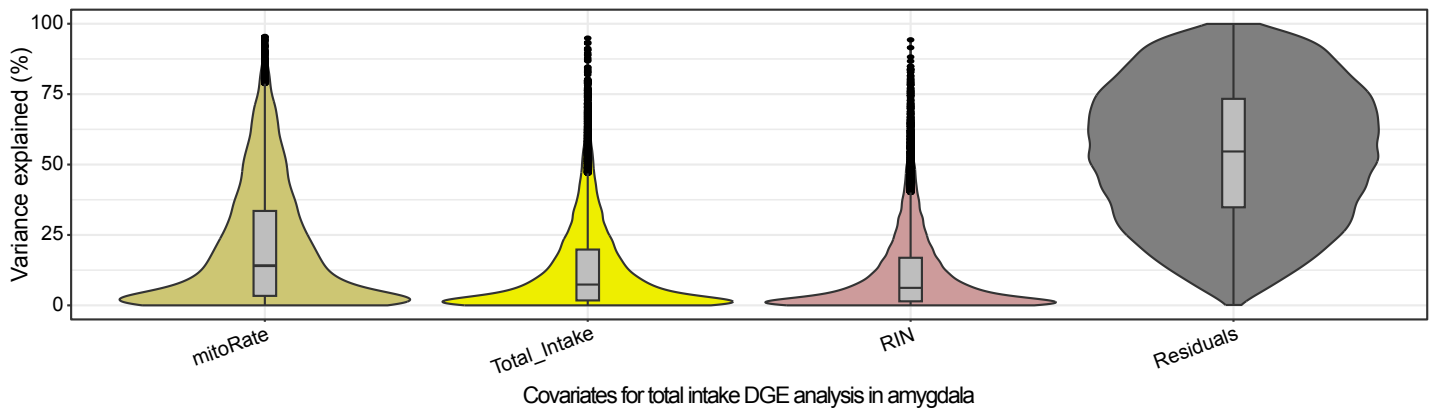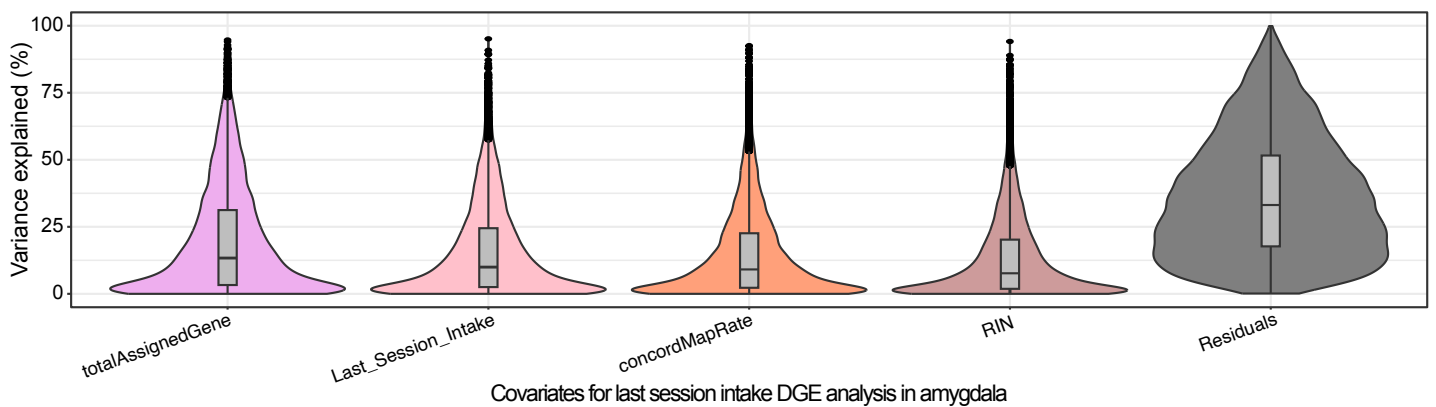

Supplement: Supplementary file 9 — Figure S8: Sample‐level covariate selection for DGE analysis. Gene expression variance partition analysis in (A) Hb (all rats), (B) Amyg (all rats), (C) Hb (fentanyl rats only) and (D) Amyg (fentanyl rats only). Top left: density plot for the percentages of variance explained in the expression of each gene by each sample‐level variable. Top right: canonical correlation between each pair of variables. Variables included in the models for DGE analyses (A–B for substance and C–D for rat behavioural traits) were selected based on their contributions to gene expression variance and correlations with other variables. Bottom: percentage of variance in the expression of each gene explained by each variable included in the DGE model, considering all other included variables in the model (x‐axis); variables are ordered by decreasing median percentage of variance explained. Related to Figure 2. See Table S3 for the description of these variables and QC metrics. [file ADB-31-e70179-s003.pdf]

A

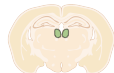

Hb

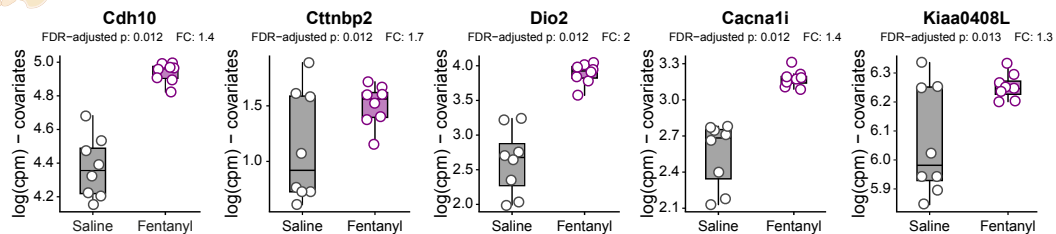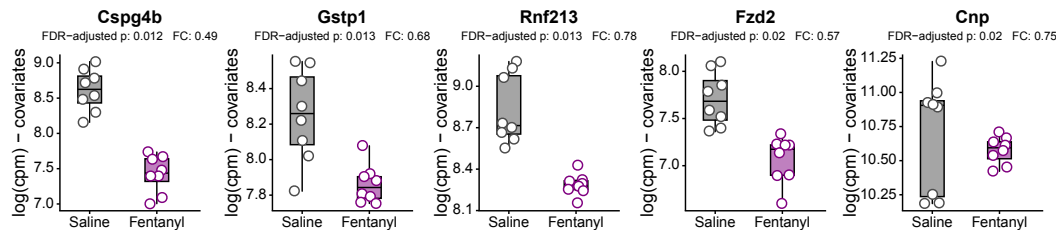

B

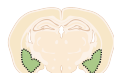

Amyg

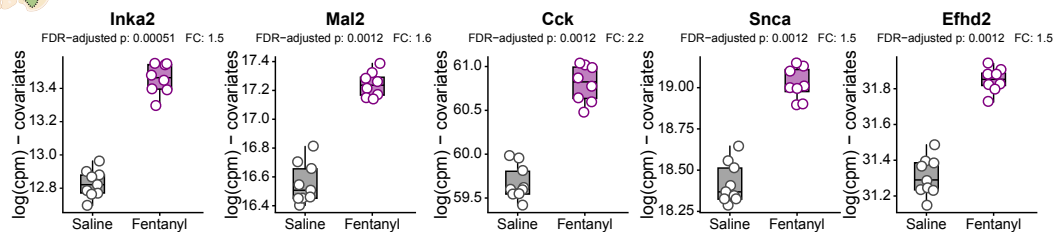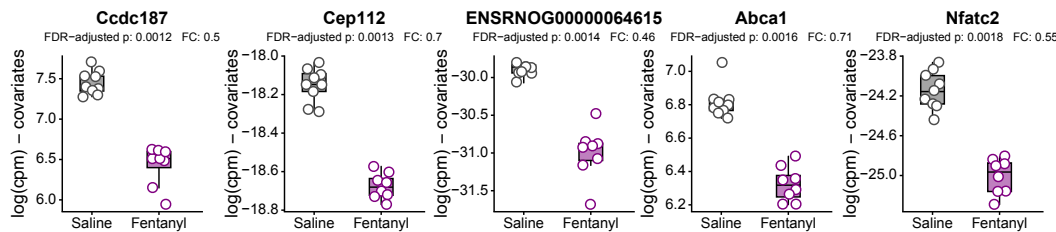

Supplement: Supplementary file 10 — Figure S9: Top 5 differentially expressed genes in Hb and Amyg following chronic LgA fentanyl self‐administration. (A–B) Box plots showing the expression of the top five up‐ and down‐regulated DEGs for fentanyl versus saline in Hb (A) and Amyg (B). Gene expression is given in log2(CPM) after regressing out covariates. FDR‐adjusted p‐value and fold change (FC) are shown for each gene. Boxes extend from the 25th to 75th percentiles; lines within the boxes represent the median; whiskers indicate the minimum and maximum values, superimposed with individual rat data points. Fentanyl Hb n = 8; saline Hb n = 8; fentanyl Amyg n = 8; saline Amyg n = 9. Related to Figure 2. [file ADB-31-e70179-s009.pdf]

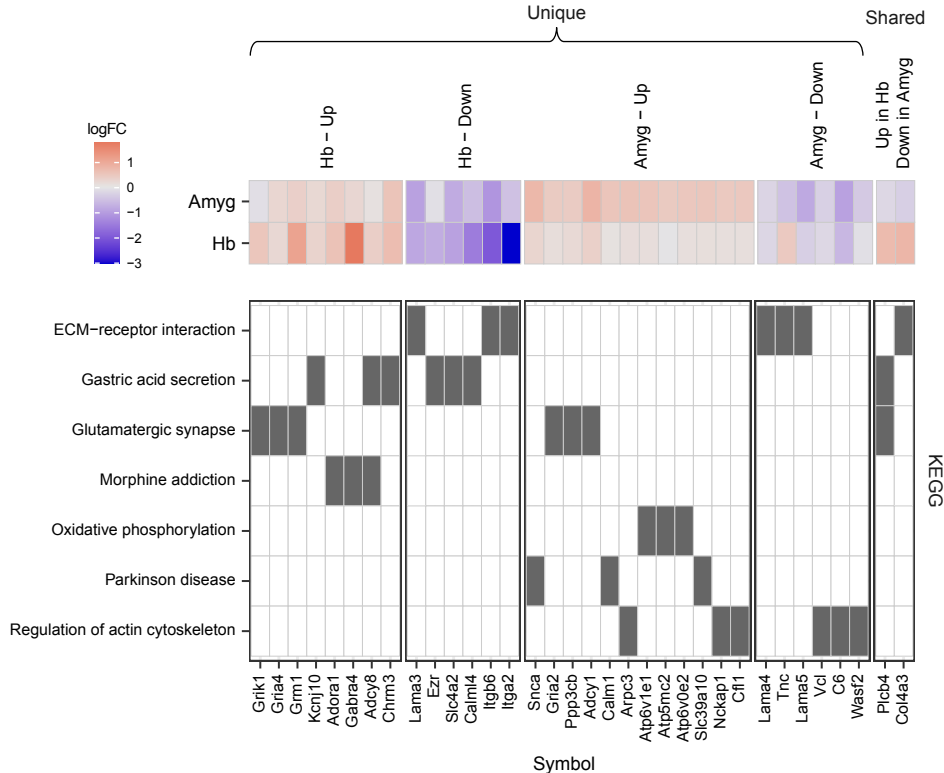

Supplement: Supplementary file 11 — Figure S10: Biological KEGG pathways dysregulated by chronic fentanyl self‐administration in Hb and Amyg. Tile plot displays DEG (x‐axis) membership to an enriched pathway as a filled tile. Key DEGs from each pathway are shown, categorized by their unique or shared up‐ and down‐regulation in Hb and Amyg. Top heatmap shows DEG mean‐centred log2FC in Hb and Amyg. Related to Figure 2 and Tables S8 and S9. [file ADB-31-e70179-s008.pdf]

**A**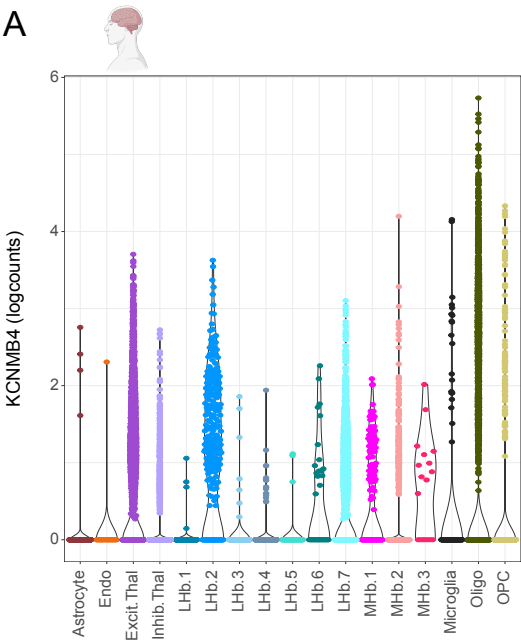**B**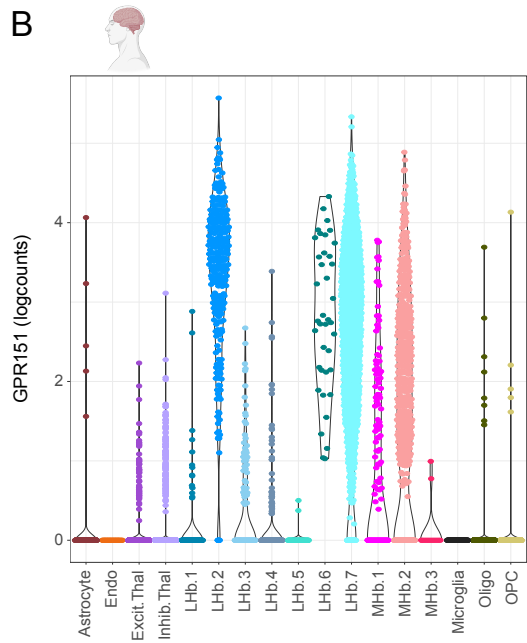**C**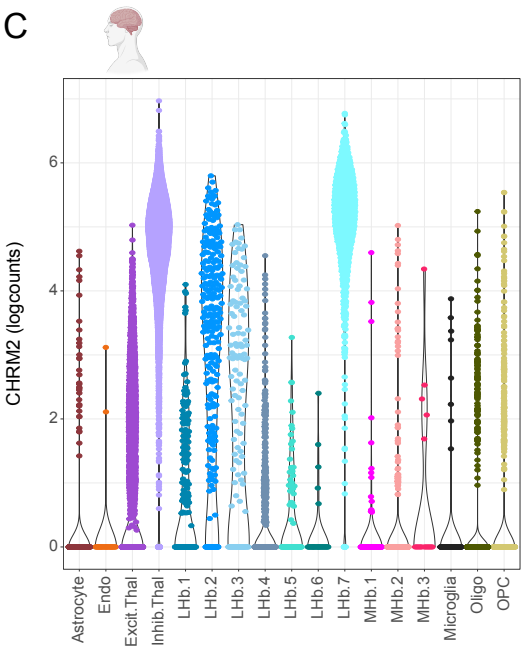**D**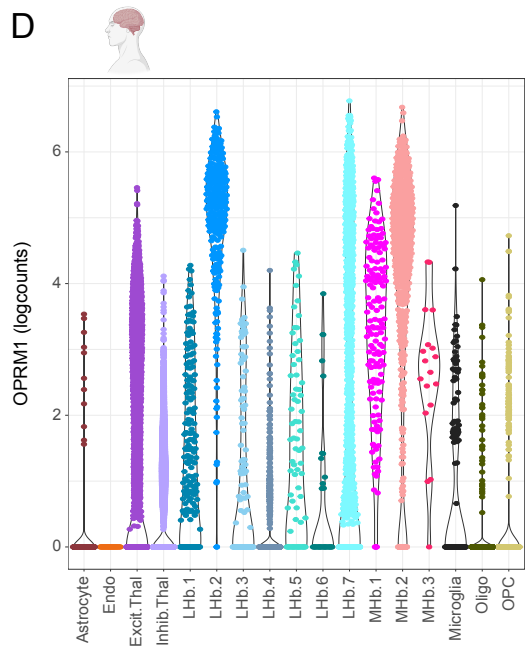

Supplement: Supplementary file 12 — Figure S11: Expression of KCNMB4, GPR151, CHRM2 and OPRM1 in human habenula cell types. (A–D) Violin plots showing expression of (A) KCNMB4, (B) GPR151, (C) CHRM2 and (D) OPRM1 in human Hb cell types from Yalcinbas et al. [49]. Kcnmb4, Gpr151 and Chrm2 mark the mouse LHb.6 subpopulation identified by Hashikawa et al. [50], which we found to be enriched in our rat upregulated Hb fentanyl DEGs. These genes are highly expressed in human LHb.2 and LHb.7 subpopulations, which also express OPRM1. This suggests that fentanyl‐sensitive mouse LHb.6 may be conserved with these OPRM1‐expressing human LHb.2 and LHb.7 neuronal populations. Related to Figure 3. [file ADB-31-e70179-s022.pdf]
