# Supplementary material for: Transcriptional Response to Chronic Long‐Access Fentanyl Self‐Administration in Rat Habenula and Amygdala
Source: Addict Biol. 2026 Jul 14;31(7):e70179. doi: 10.1111/adb.70179 (PMC13366401; doi:10.1111/adb.70179)
Supplement: Supplementary file 5 — Figure S4: Quality control metrics for samples across total number of self‐administration sessions. Comparison of QC metrics for (Hb and Amyg) samples from rats who had 22 and 24 total (fentanyl or saline) self‐administration sessions. See Table S3 for the description of these QC metrics. [file ADB-31-e70179-s025.pdf]

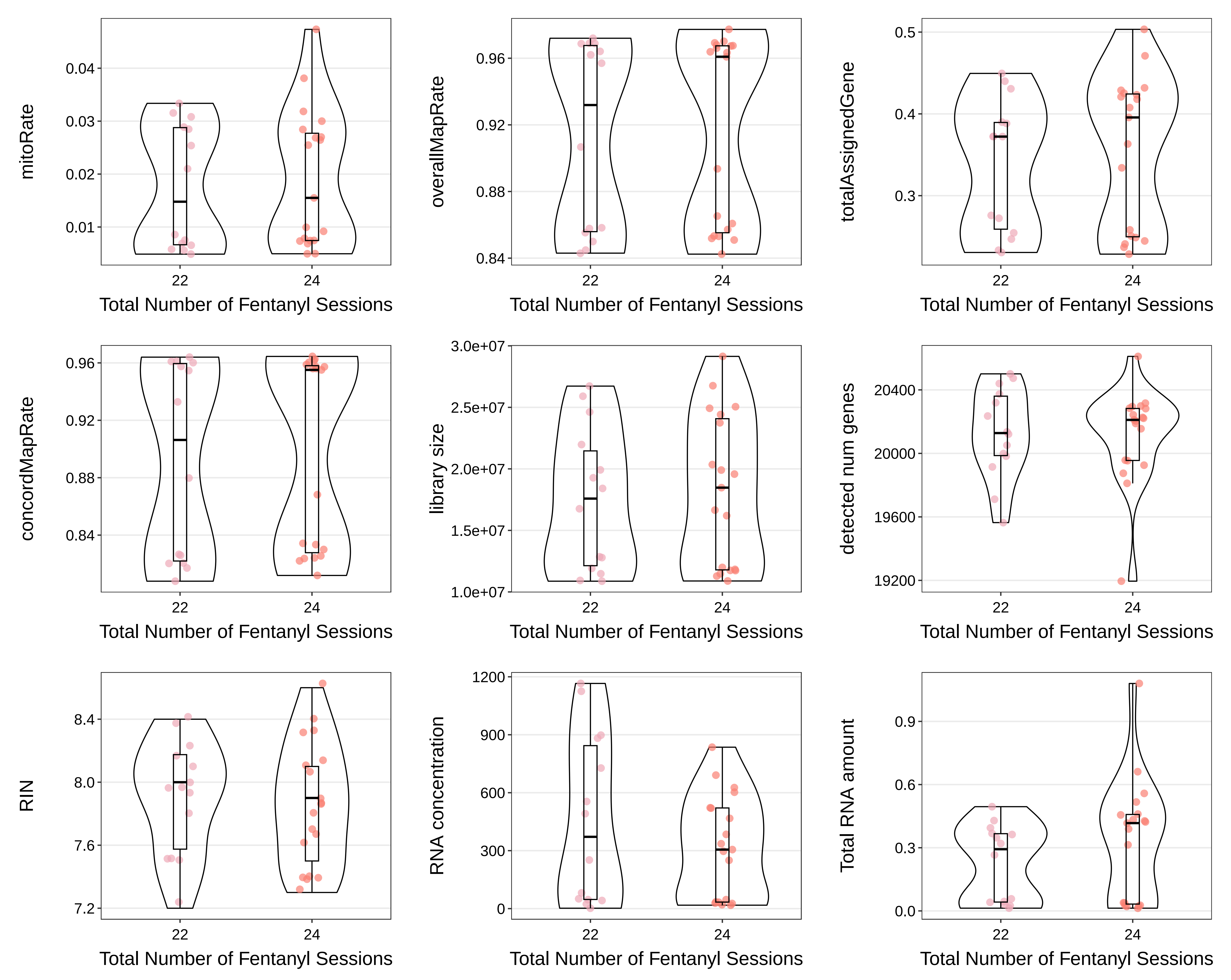

mitoRate
0.04
0.03
0.02
0.01
overallMapRate
0.96
0.92
0.88
0.84
totalAssignedGene
0.5
0.4
0.3
concordMapRate
0.96
0.92
0.88
0.84
library size
3.0e+07
2.5e+07
2.0e+07
1.5e+07
1.0e+07
detected num genes
20400
20000
19600
19200
RIN
8.4
8.0
7.6
7.2
RNA concentration
1200
900
600
300
0
Total RNA amount
0.9
0.6
0.3
0.0
22
24
Total Number of Fentanyl Sessions
